# Interpretable Deep Supramolecular Language Modeling for Cocrystal Design: PCA-Augmented *DeepCocrystal* Screening of Picolinamide, Nicotinamide, and Isonicotinamide Systems

**DOI:** 10.3390/molecules31183354

**Published:** 2026-09-21

**Authors:** Bahadır Bozkurt, Ayberk Yilmaz, Gulce Ogruc Ildiz, Rui Fausto

**Affiliations:** 1 Spectroscopy@IKU, IKU-SPECTRA Molecular Sciences and Spectroscopy Applied Research Center, Istanbul Kultur University, Atakoy Campus, Bakirkoy, Istanbul 34156, Türkiye; ayberk@istanbul.edu.tr (A.Y.); g.ogruc@iku.edu.tr (G.O.I.); rfausto@ci.uc.pt (R.F.); 2 Faculty of Science, Department of Physics, Istanbul University, Vezneciler, Fatih, Istanbul 34134, Türkiye; 3 Department of Physics, Faculty of Sciences and Letters, Istanbul Kultur University, Atakoy Campus, Bakirkoy, Istanbul 34156, Türkiye; 4 CQC-IMS, Department of Chemistry, University of Coimbra, 3004-535 Coimbra, Portugal

**Keywords:** cocrystallization, *DeepCocrystal*, ΔpK_a_ post-filtering rule, PCA interpretation, pyridinecarboxamides (picolinamide, nicotinamide, isonicotinamide)

## Abstract

Cocrystallization is an effective strategy for modifying the physicochemical properties of active pharmaceutical ingredients (APIs), yet rational coformer selection remains challenging. In this work, we propose an interpretable extension of *DeepCocrystal* by integrating Principal Component Analysis (PCA) with supramolecular language-based prediction. Picolinamide, nicotinamide, and isonicotinamide were selected as models for APIs due to their diverse hydrogen-bonding capabilities but structural similarity. A curated coformer library was screened computationally, and the molecular descriptor space was analyzed to identify dominant structural drivers of cocrystal compatibility. PCA revealed that cocrystal propensity in the studied systems is primarily governed by hydrogen-bond complementarity, polarity matching, and aromatic surface interactions. The integrated framework is intended to enhance mechanistic interpretability without altering the predictive output of *DeepCocrystal*. In this setting, the PCA layer functions as a post hoc, unsupervised readout of the descriptor space, so the original ranking and scoring of the predictions remain unchanged. This approach provides a scalable and chemically transparent methodology for rational coformer prioritization and supports data-driven crystal engineering strategies. In addition, a simpler post-filtering strategy based on pK_a_ values was also applied to the *DeepCocrystal* predictions to attempt a classification that can exclude salts. Following the pK_a_-based post-filtering rules, from the 137 API–coformer pairs classified by *DeepCocrystal* as positive or as uncertain, that is, all pairs not confidently excluded, 46 pairs retained their classification as high-probability cocrystal-compatible, 39 pairs were assigned the classification of uncertain but salt formation excluded, 11 pairs retained the classification of negative with large uncertainty, 34 pairs were reclassified as belonging to the salt/cocrystal “gray zone”, and 7 pairs were classified as salt-risk candidates.

## 1. Introduction

Cocrystallization has become a central strategy in pharmaceutical solid-state science because it enables the modification of key physicochemical properties of active pharmaceutical ingredients (APIs), including solubility, dissolution behavior, stability, hygroscopicity, and mechanical performance, without requiring covalent modification of the pharmacologically active molecule. Pharmaceutical cocrystals are therefore increasingly regarded as rationally designable multicomponent solids and have become an important part of modern preformulation and crystal engineering workflows, positioning them as a practical route toward improved medicines [1,2].

Despite this potential, the rational selection of suitable coformers remains a major challenge. Cocrystal formation depends on a complex balance of supramolecular recognition, molecular complementarity, lattice stabilization, conformational adaptability, and crystallization conditions. Traditional screening strategies often rely on empirical solvent-assisted grinding, slurry or slow evaporation, as well as melt crystallization, sublimation, and mechanochemistry-based approaches, which can be time-consuming when the possible API–coformer chemical space is large. Computational approaches for cocrystal screening have also been developed to prioritize promising molecular pairs before experimental screening, such as those based, for example, on hydrogen-bond propensity, molecular complementarity, pK_a_ differences, or molecular electrostatic potentials, as well as more sophisticated methods of crystal structure prediction relying on density functional theory (DFT, e.g., periodic DFT calculations [3,4,5,6,7]), or quantum-chemistry-based statistical thermodynamics (e.g., the COSMO-RS method [8]).

More recently, machine-learning-based approaches that use different types of descriptors have been proposed, highlighting the value of combining chemically interpretable descriptors with predictive screening [7,9,10]. *DeepCocrystal* is a deep-learning-based computational framework developed for the prediction of cocrystal formation between pairs of molecules that, unlike traditional approaches that rely on empirical rules, hydrogen-bond propensity, or thermodynamic descriptors, employs neural network architectures trained on large databases of known cocrystals to automatically learn structural and chemical features associated with successful cocrystallization [11]. It uses molecular SMILES representations as input data and outputs the probability that a given molecular pair will form a cocrystal. The model demonstrated superior predictive performance relative to previous machine-learning approaches and was successfully validated through the prospective discovery of new cocrystals [11]. *DeepCocrystal* modeling was reported to reach an accuracy of 78% under realistic external validation conditions [11]. However, the interpretation of AI-generated cocrystal predictions requires caution. Besides the general problems associated with AI-based methods, such as training-set bias, a binary “cocrystal/no-cocrystal” prediction does not, for example, distinguish between molecular cocrystals and other possible multicomponent crystalline outcomes (e.g., salts, eutectics, solvates). This distinction is especially important in acid-base systems, where the boundary between a salt and a cocrystal is not absolute. Consequently, easily applicable post-validation or filtering procedures that might reinforce the reliability of the AI-generated cocrystal predictions appear fundamental for increasing the practical utility of these methods.

**Figure 1:**
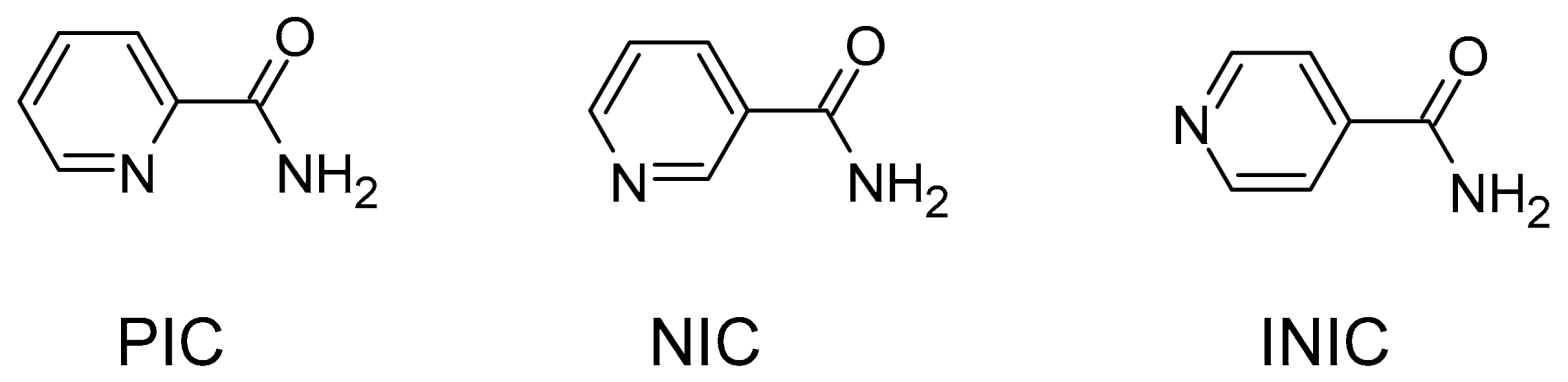
Structure of picolinamide (PIC), nicotinamide (NIC) and isonicotinamide (INIC).

In the present study, the *DeepCocrystal* prediction outputs [11] were further analyzed using two different approaches: (i) a PCA-based multivariate strategy for dimensionality reduction and improved interpretation of the predictions, and not for their re-scoring, and (ii) a simple post-filtering method based on pK_a_-derived rules. The methods are applied using pyridinecarboxamide positional isomers (picolinamide, nicotinamide, and isonicotinamide; Figure 1) as models for APIs. These compounds possess the same molecular formula and functional groups, but differ in the relative position of the pyridine nitrogen and the carboxamide group, which leads to changes in hydrogen-bond accessibility, electrostatic complementarity, and the geometry of supramolecular synthons. The three pyridinecarboxamide isomers appear to be particularly suitable for testing whether AI-assisted screening can distinguish subtle differences in supramolecular behavior among structurally similar APIs.

## 2. Methodology, Results and Discussion

**Figure 2:**
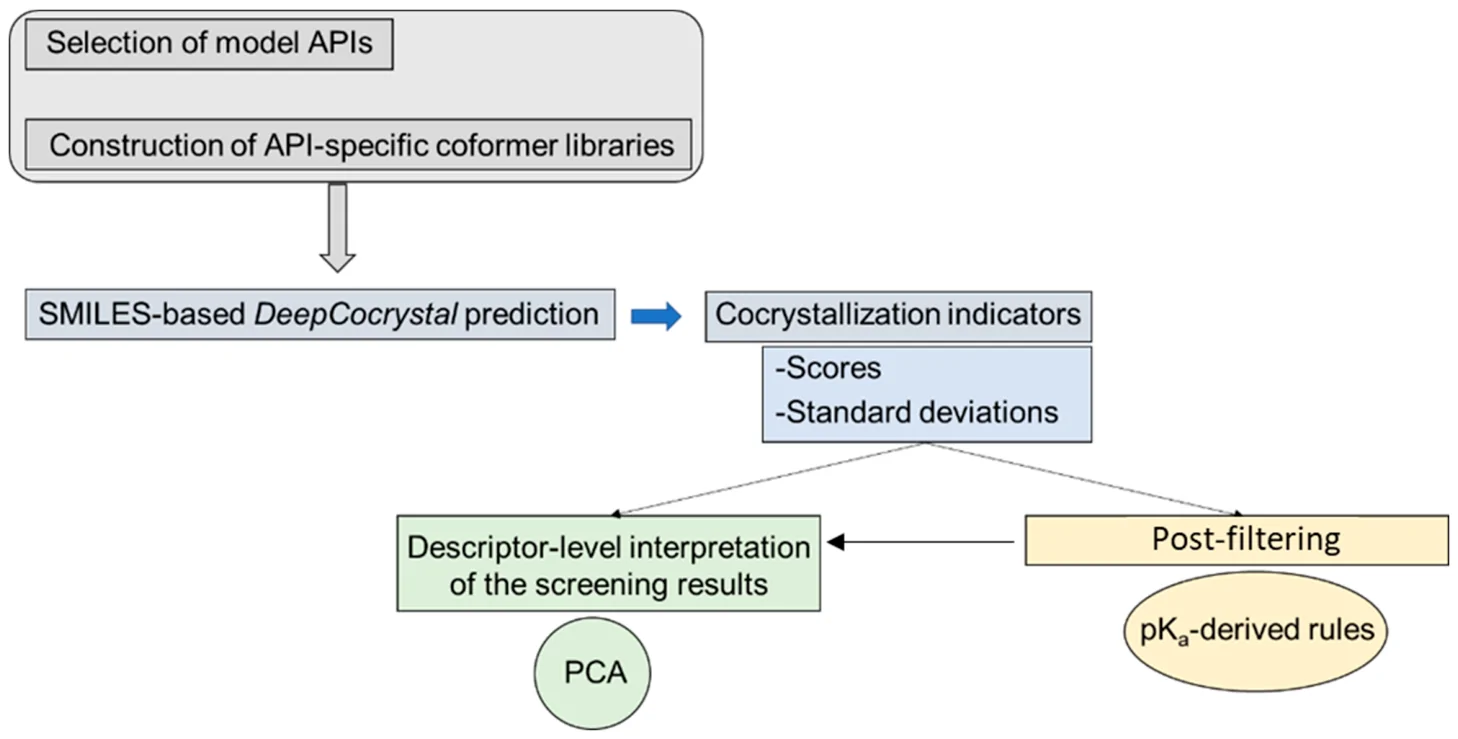
Workflow for *DeepCocrystal* descriptor-level interpretation of the screening results and post-filtering based on pK_a_-value methodology developed in the present study.

The developed methodology comprises a computational cocrystal screening workflow combining *DeepCocrystal* deep-learning-based prediction with (i) descriptor-level multivariate PCA interpretation and (ii) a simpler post-filtering based on pK_a_-derived rules. The overall workflow (Figure 2) consisted of five sequential stages: (1) selection of model APIs; (2) construction of API-specific coformer libraries; (3) SMILES-based *DeepCocrystal* prediction, providing prediction scores and standard deviations as AI-derived cocrystallization indicators; (4) PCA, for descriptor-level interpretation of the screening results; and (5) post-filtering based on pK_a_-derived rules. Because every stage of this workflow is computational and each stage is interpreted immediately in the light of the output of the preceding one, the methodological description and the corresponding results are presented together in this section rather than in separate sections. The five stages are therefore introduced in the order in which they are executed, and the outcome of each is discussed at the point where it is obtained; the reader can follow the screening from the coformer library to the final candidate list without moving between sections.

The three pyridinecarboxamide isomers (PIC, NIC, and INIC) were selected as model APIs. A library of 50 potential coformers (including PIC, NIC and INIC) was assembled to ensure both chemical diversity and supramolecular relevance, taking into account the existing knowledge regarding their ability to form cocrystals with the selected model compounds or structurally related molecules (Table 1). For each API model, the library was divided into four evidence-based subsets (Tables S1–S3, in the Supplementary Materials) for subsequent inter-group comparisons: subgroup A: molecules for which the corresponding API–coformer cocrystal has been reported (*directly reported API cocrystals* coformer subgroup); subgroup B: molecules that have been shown to form a cocrystal with an API that is structurally very similar to the API model compound—in the present case, the pyridinecarboxamide isomers of the considered API model (*analogue-supported API cocrystals* coformer subgroup); subgroup C: other compounds obeying known favorable cocrystal-engineering principles—in the present case, molecules such as those potentially able to form acid–pyridine heterosynthon, amide–amide dimer or chain motifs, exhibiting hydrogen-bond donor/acceptor complementarity, aromatic stacking potential, similar polarity to that of the API, or conformational adaptability (*rule-based-probable* coformer subgroup); and subgroup D: molecules with negative reporting or expected structural incompatibilities with the API—in the case of the pyridinecarboxamide API models, molecules lacking strong hydrogen-bond donor/acceptor sites, or showing excessive hydrophobicity, very high conformational flexibility, likely salt formation (large pK_a_ difference relative to the API), or eutectic-forming tendency (*rule-based-low-probability* coformer subgroup).

*DeepCocrystal* prediction scores for the different API–coformer pairs were then obtained. Prediction outputs were classified using a two-level decision scheme based on the prediction score and its associated uncertainty. First, the mean prediction score (*p*) was used to assign the API–coformer pair to a positive or negative class. Pairs with *p* ≥ 0.50 were considered positive *DeepCocrystal* predictions, indicating predicted cocrystal-forming propensity, whereas pairs with *p* < 0.50 were considered negative predictions. Second, the standard deviation (σ) obtained from repeated/augmented SMILES predictions was used as an uncertainty estimator. Predictions with σ ≤ 0.10 were considered high-certainty predictions, while those with σ > 0.10 were considered uncertain. Accordingly, pairs with *p* ≥ 0.50 and σ ≤ 0.10 were classified as high-priority candidates (HPC), pairs with *p* ≥ 0.50 and σ > 0.10 as low-priority candidates type 1 (LPC1), pairs with *p* < 0.50 and σ ≤ 0.10 as reliable negative candidates (NC), and pairs with *p* < 0.50 and σ > 0.10 as low-priority candidates type 2 (LPC2). These results are shown in Table 2, in which the font color of each label repeats the class: green, HPC; black, LPC1; blue, LPC2; red, NC. The used thresholds were directly extracted from the original *DeepCocrystal* work [11], in which restricting predictions to this standard-deviation range improved external-set performance. Therefore, they are treated in the present investigation as literature-derived operational criteria rather than as parameters optimized for the present dataset.

According to the defined criteria, 75 pairs were classified as high-certainty positive predictions, while 62 were classified as uncertain (48 LPC1 and 14 LPC2) in the present dataset, with 10 pairs being classified as non-compatible (NC). The largest number of *DeepCocrystal*-positive predictions (*p* ≥ 0.50; HPC and LPC1) was found for INIC, with 44 positive pairs out of 49, with 38 being HPC group members. This suggests that the INIC scaffold should be considered the most favorable among the three pyridinecarboxamide isomers in terms of supramolecular complementarity with the considered set of coformers.

**Figure 3:**
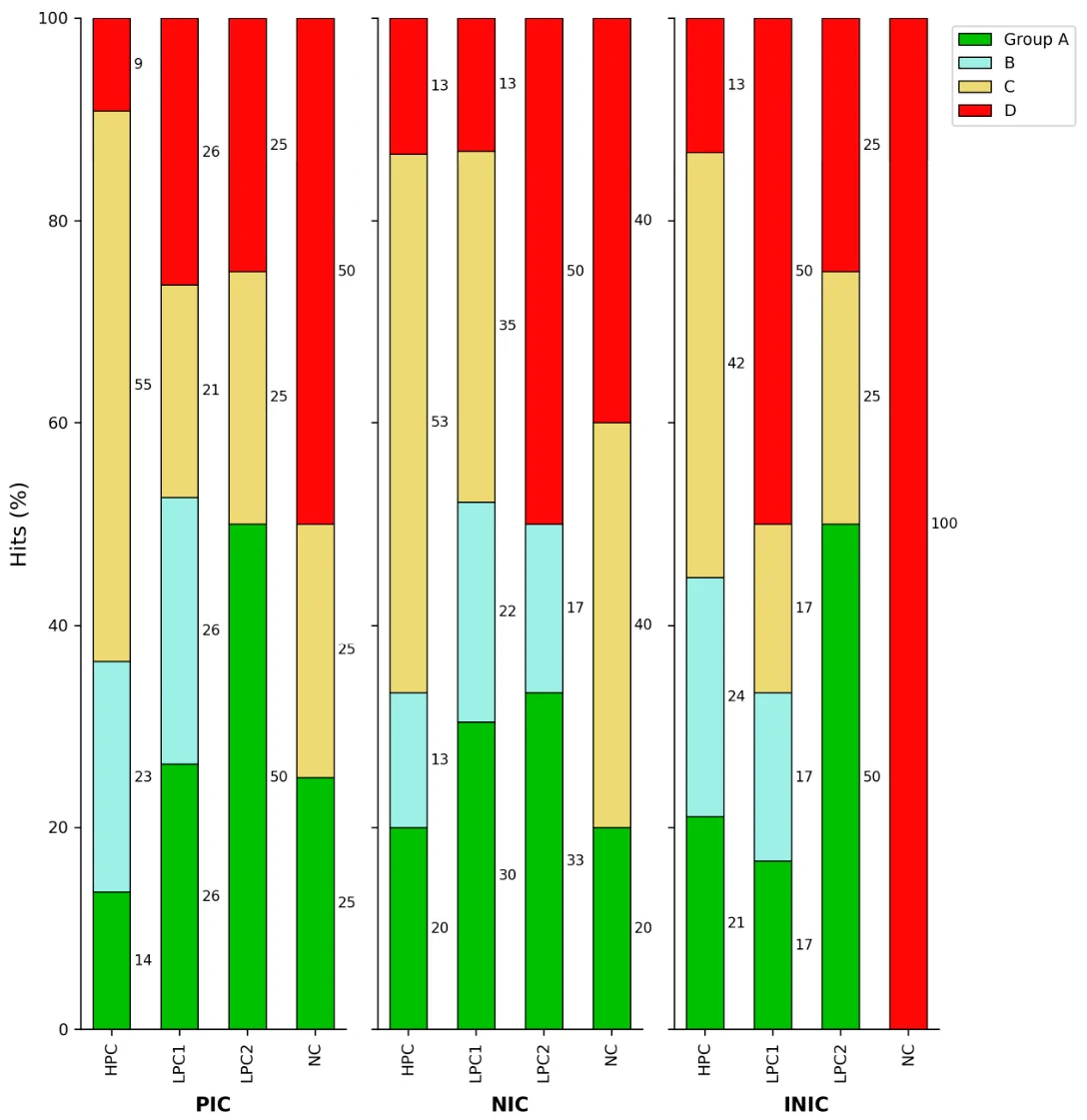
*DeepCocrystal* classifications for PIC, NIC and INIC across the literature-evidence subgroups, expressed as the percentage composition of each certainty class. Group A, directly reported API–coformer cocrystal; group B, analogue-supported pyridinecarboxamide cocrystal; group C, rule-based probable candidate; group D, rule-based low-probability candidate.

Figure 3 presents the distribution of the different *DeepCocrystal* classifications for each API by evidence-based groups, showing that the two sets of data are in good general qualitative agreement, in particular for PIC and INIC, where coformers assigned evidence-based groups A and B were mostly classified as HPC or LPC1, and those assigned evidence-group D as LPC2 and NC. Coformers assigned group C were classified broadly as HPC, LPC1 and LPC2 for the three APIs.

A post-filtering of the *DeepCocrystal*-generated results was then performed using empirical rules based on the pK_a_ values [12,13], which were applied as a post-classification filter to evaluate an API–coformer pair not excluded by *DeepCocrystal* (those belonging to the HPC, LPC1 and LPC2 groups) is more consistent with cocrystal formation, or whether salt formation should be considered. The used pK_a_ values were extracted from the literature [14], except for that of 2,6-dihydroxybenzoic acid, for which the value redetermined by Lowe and Smith (1.229 ± 0.045 by e.m.f. and 1.230 ± 0.016 by conductance in water at 25 °C) was adopted [15] (Table S4). In the case of non-ionizable coformers, the ΔpK_a_ rule is not applicable, and in these cases, the *DeepCocrystal* prediction was retained.

For each API–coformer, ΔpK_a_ = pK_a_(API-H^+^) − pK_a_(coformer) was calculated, where pK_a_(API-H^+^) corresponds to the conjugate-acid pK_a_ of the API, and pK_a_(coformer) corresponds to the pK_a_ associated with the most acidic proton of the coformer. The *DeepCocrystal* classification was then reviewed according to the following empirical rules: (i) when ΔpK_a_ < 0, neutral cocrystal formation is considered more likely than salt formation, because proton transfer from the coformer to the API is not expected and the *DeepCocrystal* classification is retained; (ii) when 0 ≤ ΔpK_a_ ≤ 3, the pair is assigned to the salt/cocrystal gray zone (GZ). In this region, proton transfer may be partial, environment-dependent, or influenced by specific crystal packing, solvent, and temperature [12,13]. These candidates were reclassified as ambiguous and requiring experimental validation; (iii) when ΔpK_a_ > 3, salt formation is considered more likely, and these *DeepCocrystal* classified positive pairs were reclassified as salt-risk (SR) systems.

To make the pK_a_-based post-filtering outcome fully reproducible and to remove any ambiguity in how the summary counts quoted below and in Table 3 and Table 4 were obtained, the outcome categories are defined precisely as follows. The pK_a_ rule is applied only to the 137 API–coformer pairs that *DeepCocrystal* classified as HPC, LPC1 or LPC2; the remaining 10 pairs classified as NC are outside the scope of the pK_a_-based re-evaluation (Methods, above) and are trivially reported as “unchanged” in Table 4. Within the 137-pair pool, a pair is labeled “unchanged” if its ΔpK_a_ value places it in the cocrystal-compatible regime (ΔpK_a_ < 0), so its original *DeepCocrystal* label (HPC, LPC1 or LPC2) is retained; a pair is labeled “reclassified” if ΔpK_a_ places it in the gray zone (GZ, 0 ≤ ΔpK_a_ ≤ 3) or the salt-risk zone (SR, ΔpK_a_ > 3), which overrides the original *DeepCocrystal* label with GZ or SR, respectively; and a pair is reported under “pK_a_ not applied” whenever the coformer is non-ionizable and no ΔpK_a_ value can be defined, in which case the original *DeepCocrystal* label is retained by default, exactly as for the “unchanged” pairs, but tabulated separately for transparency. The 20 pairs counted under “pK_a_ not applied” in Table 4 are those non-ionizable pairs that fall inside the 137-pair pool; the remaining four non-ionizable pairs received an NC prediction and are counted as out of scope, which is why 24 pairs in total lie outside the scope of the rule. The HPC/LPC1/LPC2 breakdown quoted in the text for the 96 pairs retained overall (46 HPC, 39 LPC1 and 11 LPC2) is obtained by cross-tabulating this unchanged/pK_a_-not-applied outcome against the original *DeepCocrystal* class, and the equivalent per-API breakdowns discussed for PIC, NIC and INIC follow the same procedure, restricted to each API’s 49 scored pairs.

A “false negative” (Table 4) is defined strictly as a pair for which the coformer belongs to literature-evidence group A, that is an API–coformer cocrystal directly reported in the literature (Supplementary Materials, Tables S1–S3), but for which *DeepCocrystal* nonetheless returns a negative (NC) prediction. Pairs belonging to evidence groups B, C or D that happen to receive an NC prediction are, by this definition, not false negatives, since no cocrystal has actually been reported for those pairs for the prediction to contradict. The 6 LPC2 and 5 GZ cases discussed in the text are obtained analogously by cross-tabulating evidence group A against the original *DeepCocrystal* class and the final DC-pK_a_ label, respectively.

The prediction results obtained after application of the pK_a_-based post-filtering are compared with those resulting solely from the *DeepCocrystal* analysis in Table 3 and Table 4. Following the pK_a_-based post-filtering rules, from the 137 API–coformer pairs classified by *DeepCocrystal* as HPC, LPC1 and LPC2, 46 pairs retained their classification as high-probability cocrystal-compatible (HPC), 39 pairs were assigned the classification of uncertain but salt formation excluded (LPC1), 11 pairs retained the LPC2 classification, 34 pairs were reclassified as belonging to the salt/cocrystal gray zone (GZ), and 7 pairs were classified as salt-risk candidates (SR).

As highlighted above, isonicotinamide was, among the three investigated API model compounds, the one for which *DeepCocrystal* predicted the largest number of positive cocrystal coformers. However, after application of the pK_a_-based rules, INIC also showed the largest number of gray-zone candidates. Twenty-one INIC pairs (18 HPC, 2 LPC1 and 1 LPC2) were shifted from *DeepCocrystal p*-positive interpretation to the salt/cocrystal continuum (GZ and SR groups). This suggests that the comparatively high *DeepCocrystal* scores for INIC are partly associated with a stronger acid–base coformer-API complementarity for INIC, which may increase cocrystal propensity but may also increase the risk of proton-transfer (salt formation) ambiguity.

On the other hand, picolinamide showed the most stable behavior after pK_a_ correction. Forty-one pairs were initially predicted by *DeepCocrystal* with positive scores (22 HPC and 19 LPC1), and 37 of these were retained after pK_a_ filtering, while only three pairs were assigned to the gray zone and one was classified as a salt-risk candidate. This indicates that *DeepCocrystal* predictions are less affected by acid–base proton-transfer ambiguity and are more consistently compatible with molecular cocrystal interpretation in the case of PIC, where the ring nitrogen atom is less exposed.

Nicotinamide showed intermediate behavior. Although it has a number of *DeepCocrystal*-positive predictions comparable to that of PIC, the pK_a_ post-filtering retained only 7 pairs as highly cocrystal-compatible (HPC) candidates and 17 pairs as uncertain, but salt formation was excluded (LPC1), while 11 pairs were assigned to the gray zone and 3 were excluded as salt-risk systems. In addition, one LPC2 pair was assigned gray-zone status. This indicates that NIC has broad *DeepCocrystal*-predicted compatibility, but a large fraction of its positive-scoring candidates requires careful experimental validation to evaluate the cocrystal/salt ambiguity.

Overall, the pK_a_-based post-filtering significantly refined the *DeepCocrystal* output. The most relevant comparative result is that INIC shows the strongest *DeepCocrystal*-based cocrystal propensity, whereas PIC shows the most robust cocrystal profile after pK_a_ correction. Therefore, the combined *DeepCocrystal*–pK_a_ strategy suggests that INIC is the most broadly compatible scaffold, while PIC appears as the most conservative and chemically reliable compound among the three API models investigated. These conclusions highlight the usefulness of pK_a_-based post-filtering of *DeepCocrystal* results, as proposed here, as a simple but chemically meaningful filter for reducing false-positive cocrystal interpretation arising from potential salt formation.

In addition to the pK_a_-guided post-filtering of *DeepCocrystal* predictions discussed above, the evidence-based A–D classification was also used to check possible incorrect outputs. This additional verification is important because it is a test of the robustness of the approach used. Although evidence-based verification cannot be applied to the cases where no API–coformer cocrystal has been reported to judge the predictions, a negative prediction for a previously reported API–coformer cocrystal would have to be considered a false negative. Applying this definition identifies two false negatives, PIC–nicotinamide and NIC–picolinamide (both *p* = 0.017, σ = 0.040), among the 35 group A (directly reported) pairs. Both belong to the subset of six pairs in which a pyridinecarboxamide is offered as the coformer of another pyridinecarboxamide, and DeepCocrystal returns a negative score for all six of them (*p* = 0.017 for PIC–nicotinamide and NIC–picolinamide, 0.046 for NIC–isonicotinamide and INIC–nicotinamide, and 0.142 for PIC–isonicotinamide and INIC–picolinamide), although cocrystals of all three isomer combinations are reported [16]. The behavior is systematic, and it has both a representational and a chemical origin. The two components of such a pair are constitutional isomers of the same molecular formula whose SMILES strings differ only in the ring position of one substituent, so the model receives an input close to a self-pair, and self-pairs are negative instances in any cocrystal training set. The scores order accordingly: the closer the two partners are in structure, the more confident the negative. Both components also carry a primary carboxamide, and the amide–amide dimer and catemer are among the most reliable homosynthons in the Cambridge Structural Database, so a model trained largely on pairs in which the amide appears on only one component will associate amide-on-amide inputs with homomolecular association. What stabilizes these cocrystals, however, is the directional N(pyridine)···H–N(amide) heterosynthon between the two isomers, which is viable because the basicity and the steric accessibility of the pyridine nitrogen differ between the ortho, meta and para scaffolds (conjugate-acid pK_a_ 1.50, 3.35 and 3.61). That information is carried by the API-specific descriptors D16–D19, which define PC2, and it is not available to a model that reads only the two SMILES strings; in the descriptor map the six pairs fall in the region associated with viable heterosynthon formation. The two false negatives therefore mark the limit of the model within the pyridinecarboxamide family and indicate that homofunctional isomer pairs are under-represented in its training domain. Apart from these two pairs, every other previously reported API–coformer cocrystal received a *DeepCocrystal*-positive (HPC, LPC1 or LPC2) prediction. The NIC–riboflavin pair, which might appear to be a false-negative candidate because *DeepCocrystal* assigns it an NC prediction, is in fact not one: riboflavin belongs to evidence group D (rule-based-low-probability) for all three APIs, not group A, so a negative prediction for this pair is the expected, correct outcome rather than an error. Only in a few cases did the predictions assign cocrystal systems observed experimentally to the LPC2 class (negative prediction but large uncertainty; 6 cases) or GZ (gray zone for possible salt formation; 5 cases: PIC–picolinic acid, NIC–fumaric acid, NIC–picolinic acid, INIC–picolinic acid and INIC–2-fluorobenzoic acid) (see Table 3).

In order to properly contextualize the findings, we first define the scope of the PCA. Here, PCA is used only as an unsupervised, post hoc interpretability layer applied to the descriptors of pairs already scored by *DeepCocrystal*. It operates downstream and does not feed back into the neural network, re-rank the predictions, or affect the baseline metrics of accuracy, sensitivity, and false-positive rate. Its purpose is to reduce twenty-four correlated descriptors to a few orthogonal, chemically intuitive axes, so the molecular features behind a given *DeepCocrystal* score can be read in terms of familiar properties such as polarity, hydrogen-bonding capacity, aromatic surface, and heterosynthon geometry. PCA therefore improves interpretability without changing performance, and both the PCA and the pK_a_ rule act as confirmatory, diagnostic tools rather than parallel predictors.

To interpret the structural and physicochemical basis of the predicted cocrystal compatibility, PCA was performed. Molecular descriptors were selected to represent both general cocrystal formation principles and features specific to the pyridinecarboxamide systems studied here. The whole descriptor set included 15 general molecular descriptors (e.g., hydrogen-bond donor count, hydrogen-bond acceptor count, rotatable bond count, aromatic ring count, etc.) and 9 pyridinecarboxamide-specific descriptors (Table 5). Prior to PCA, descriptor values were autoscaled to unit variance: each column of the descriptor matrix was centered to zero mean and scaled to unit standard deviation (z-scores), which is equivalent to performing the PCA on the correlation matrix and ensures that no descriptor dominates the analysis merely because of its numerical scale. The standardized 147 × 24 matrix was then decomposed by full singular-value decomposition (SVD). The exact definition and numerical encoding of each descriptor, including the RDKit-computed general descriptors and the API-specific descriptors, is provided in Table 5 and, for the derived descriptors that are not read directly from RDKit, in Table S6 of the Supplementary Materials. The computed values of the general descriptors are tabulated in Table S5 and the loadings of all twenty-four on the first five components in the loading heat map presented below.

PCA was applied to the standardized descriptor matrix to reduce dimensionality and to identify the principal variables responsible for the variance across the API–coformer screening space. The analysis was designed to achieve three main objectives: (i) visualization of the candidate distribution, by mapping the API–coformer pairs in the principal-component space to determine whether high-score and low-score candidates occupy distinct regions; (ii) comparison of API-specific behavior, by examining whether PIC, NIC and INIC show different descriptor-prediction relationships; and (iii) interpretation of descriptor contributions, by inspecting the PCA loadings to determine which molecular features contribute most strongly to the leading principal components.

**Figure 4:**
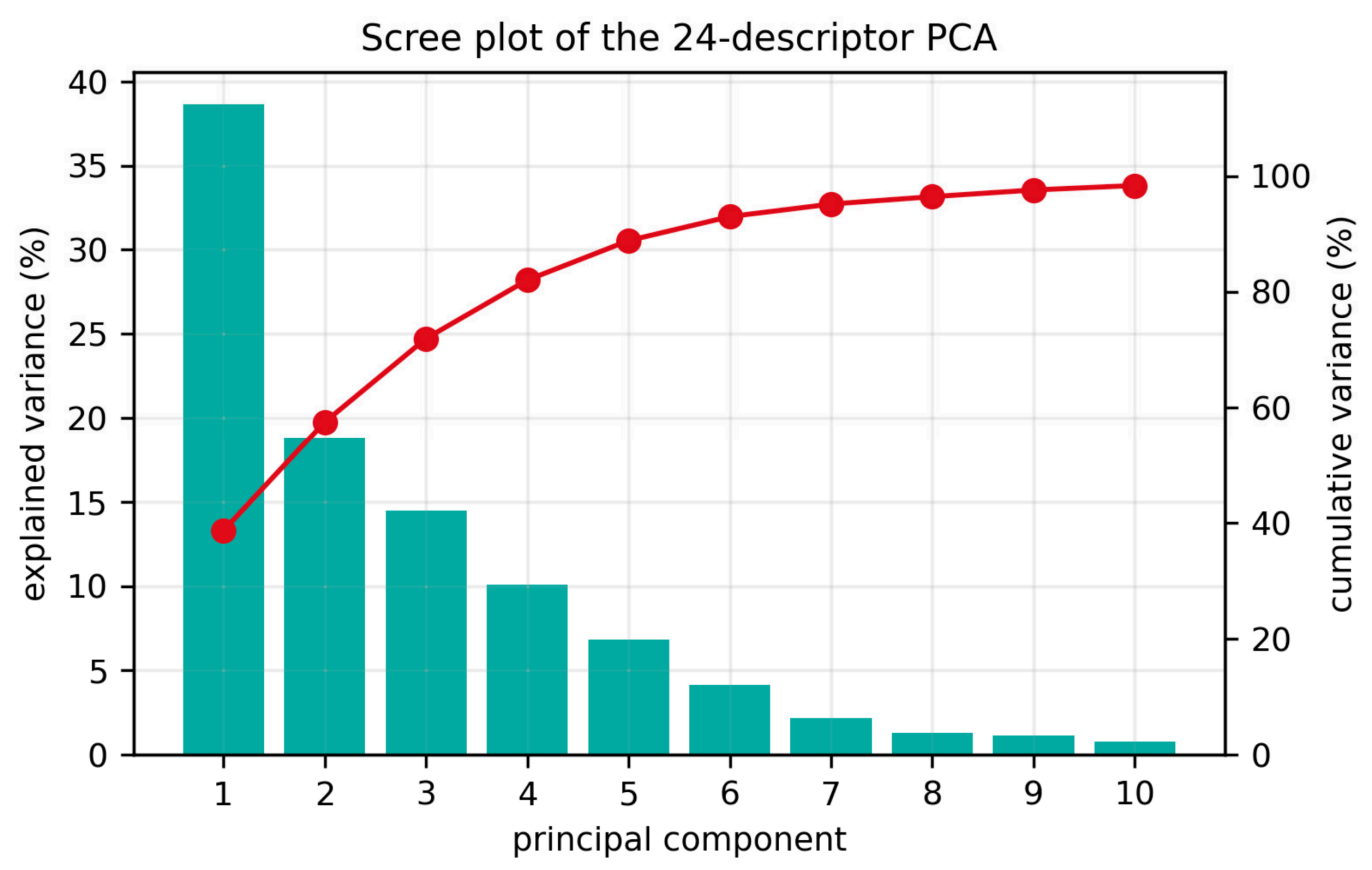
Scree plot (bars, left axis) and cumulative explained variance (line, right axis) of the 24-descriptor PCA. The first two components account for 57.4% and the first three for 71.9% of the total variance; five components satisfy the Kaiser eigenvalue > 1 criterion.

Singular-value decomposition of the autoscaled 147 × 24 descriptor matrix yielded the variance structure summarized in Table 6 and Figure 4. The first principal component accounts for 38.6% of the total variance, the second for 18.8% and the third for 14.5%, so the two-dimensional projection retains 57.4% of the variance and the three-dimensional projection 71.9%. Based on the Kaiser criterion, five components carry an eigenvalue greater than one and are individually informative, while the scree curve bends most visibly after the third component; the two- and three-dimensional projections used throughout this work are therefore well justified as faithful low-dimensional summaries.

All computations described above and in the sections that follow were carried out with a single, deterministic, fully open-source computational pipeline, so the exact software and packages used to perform the PCA can be stated precisely. The 24 molecular descriptors were computed directly from the SMILES representations with RDKit (v.2026.03); autoscaling of the descriptor matrix was performed with scikit-learn’s StandardScaler and the principal component analysis itself with scikit-learn’s PCA implementation (sklearn.decomposition.PCA, v.1.8.0) using the deterministic full-SVD solver (svd_solver = ‘full’), so no random initialization, subsampling or iterative approximation was involved and the decomposition is exactly and identically reproducible across runs and across machines. NumPy (v.2.4) and pandas (v.3.0) were used for the underlying numerical operations and data handling, and Matplotlib (v.3.10) was used to render the figures. The entire analysis (from raw SMILES input to final PCA output) can be reproduced independently using only publicly available, open-source software.

### 2.1. Determination of the Number of Significant Principal Components

The Kaiser eigenvalue-greater-than-one criterion and the visual location of the scree elbow are both informal, and neither on its own establishes that a component carries genuine, non-chance covariance structure. A third, statistically grounded retention criterion, Horn’s parallel analysis, was therefore added. The columns of the autoscaled 147 × 24 descriptor matrix were independently permuted 1000 times, which destroys the descriptor covariance structure while preserving each descriptor’s own marginal distribution. A principal component analysis was refitted to each permuted matrix, yielding, for every component, the null distribution of eigenvalues expected in the absence of any real covariance structure. A component is retained if its observed eigenvalue exceeds the 95th percentile of this null distribution.

As shown in Table 7, the observed eigenvalue exceeds the 95th-percentile permutation-null threshold for five components (PC1–PC5), matching the Kaiser eigenvalue > 1 count and confirming that the leading components carry genuine covariance structure rather than sampling noise. It should be emphasized that the restriction of detailed chemical interpretation to PC1–PC3 in the sections that follow is a deliberate editorial choice, not a claim that PC4 and PC5 are statistically meaningless: PC1–PC3 already account for 71.9% of the total variance and correspond to three cleanly separable, chemically nameable axes (polarity and hydrogen-bonding capacity, molecular size and aromatic surface, and ionization together with synthon competition). The fourth and fifth components, although statistically above the permutation baseline, mix smaller and more specific contrasts among the correlated descriptor blocks identified below and do not add a distinct, independently nameable chemical axis of comparable clarity. The full ten-component spectrum is reported in Table 6, so readers wishing to examine the higher components have the complete numerical basis to do so.

**Figure 5:**
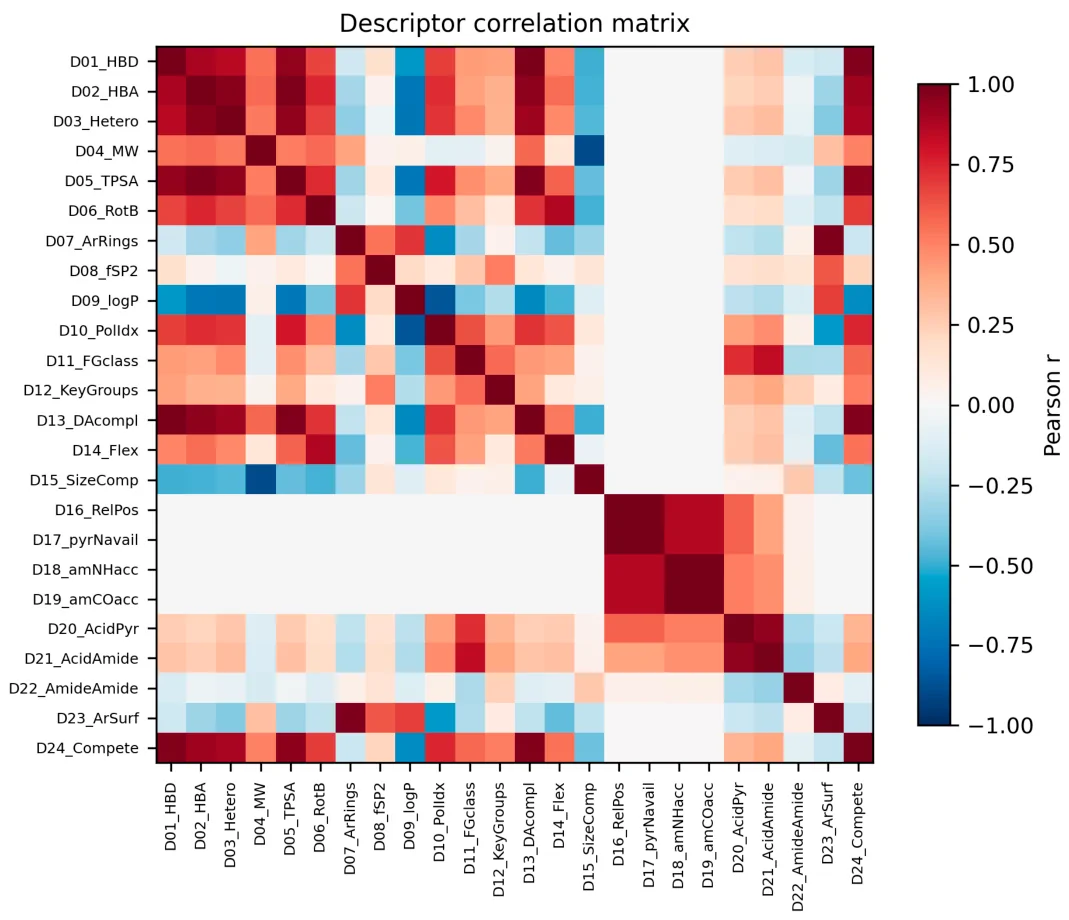
Pearson correlation matrix of the 24 descriptors over the 147 API–coformer pairs. Blocks of strongly correlated descriptors (acid-/donor-related and polarity-related groups) justify dimensionality reduction by PCA. The color scale encodes the Pearson correlation coefficient, r (dimensionless, −1 to +1).

The descriptor correlation structure underlying the PCA is shown in Figure 5. Blocks of mutually correlated descriptors are apparent: the size/polarity family (molecular weight, polar surface area, heteroatom and acceptor counts) forms one coherent block, while the API-specific geometry descriptors form a second, near-collinear block because they take only three distinct values across the dataset. This redundancy is what allows a small number of components to capture the majority of the variance. Here, r denotes the Pearson product–moment correlation coefficient between each pair of descriptors (dimensionless, ranging from −1 to +1), shown as the color scale of the map.

### 2.2. Descriptor Redundancy and Multicollinearity Assessment

The 24-descriptor set was assembled to provide a chemically complete representation of size, polarity, hydrogen-bonding capacity, aromaticity and API-specific geometry rather than to constitute a mutually independent basis, and several descriptors are consequently correlated by construction (for example, molecular weight, topological polar surface area, hydrogen-bond acceptor count and heteroatom count all grow together as a coformer becomes larger and more polar). The extent of this redundancy was quantified along two complementary lines. First, the full Pearson correlation matrix of the 24 descriptors was computed over the 147 API–coformer pairs, and all pairs with |r| ≥ 0.80 were listed (Table 8): 27 of the 276 possible descriptor pairs meet this threshold, and 12 of them exceed |r| = 0.94, confirming the correlated-block structure visible in Figure 5. Second, the variance inflation factor (VIF), computed as the diagonal of the Moore–Penrose pseudo-inverse of the correlation matrix of the autoscaled 147 × 24 matrix, which is exactly singular because the four API-scaffold descriptors form two exactly collinear pairs (r = 1.000 for D16_RelPos with D17_pyrNavail and for D18_amNHacc with D19_amCOacc, the two pairs themselves correlating at r = 0.866), was calculated for every descriptor (Table 9): all 24 descriptors exceed the conservative VIF > 5 screening threshold, and 21 exceed the conventional VIF > 10 threshold for severe multicollinearity, with the polarity/hydrogen-bonding block (D05_TPSA, D02_HBA, D01_HBD) reaching VIFs in the hundreds. Together, these two diagnostics show that the descriptor set is far from an independent basis; the redundancy is chemically expected and, as discussed below, is precisely what allows a small number of principal components to capture most of the total variance.

Despite this pronounced redundancy, no descriptors were removed prior to the PCA, for three principled reasons. First, PCA is specifically designed to absorb correlated inputs: rather than inflating the retained dimensionality, redundant descriptors load jointly onto the same component, which is precisely the mechanism that produces the compact, chemically interpretable loadings discussed in the sections that follow (the size/polarity block and the API-geometry block each collapse onto a single dominant direction). Second, any descriptor pre-selection would require an external, and inevitably supervised, redundancy criterion, whereas the aim of the present analysis is an unsupervised, purely descriptor-driven map that is then compared post hoc with the *DeepCocrystal* prediction, the literature evidence and the pK_a_-based post-filtering rule; pre-selection would risk discarding a descriptor that is redundant on average but locally informative for one of those external labels. Third, the 24-descriptor scheme is fixed by the parent methodology for direct comparability across systems, and altering it would forfeit that correspondence. The practical consequence of the redundancy is instead absorbed by the eigenvalue spectrum itself: the correlated blocks quantified in Table 8 and Table 9 are what allow five components (parallel analysis, above) and, in particular, only three chemically interpretable axes (below) to capture the substantive variance of the system. Consequently, the per-descriptor loadings reported later should be read as the joint signature of a correlated block rather than as independent evidence for each individual descriptor.

The PCA score plot revealed a partial separation between candidates predicted as cocrystal-likely and those classified as no-cocrystal. For clarity, the 2D score plots color-coded by the binary *DeepCocrystal* outcome and by the four certainty classes have been placed in the Supplementary Materials (Figures S1 and S2), while only the key numerical centroids that directly support our interpretation are reported in the main text (Table 10 and Table 11).

Cocrystal-likely systems are defined here as all pairs with a mean *DeepCocrystal* prediction score *p* ≥ 0.50 (123 of the 147 scored pairs); the remaining 24 pairs (*p* < 0.50) are classified as no-cocrystal. Most cocrystal-likely systems were concentrated near the central region of the descriptor space, particularly around moderate PC1 and PC2 values, indicating API–coformer compatibility resulting from balanced molecular size, polarity, hydrogen-bonding capacity and aromatic character. In contrast, most of the no-cocrystal candidates were displaced toward more extreme PC1 or PC2 values, suggesting that descriptor imbalance, such as excessive hydrophobicity, limited hydrogen-bonding complementarity, high molecular size or strong deviation in polarity, reduces predicted compatibility.

The centroid positions further supported this interpretation (Table 10 and Table 11). The cocrystal-likely centroid lies at (PC1 = +0.33, PC2 = +0.14, PC3 = −0.09), whereas the no-cocrystal centroid is displaced to (PC1 = −1.69, PC2 = −0.74, PC3 = +0.45), confirming the systematic shift toward lower polarity and reduced donor–acceptor complementarity–. Applying a 2-standard-deviation criterion (in PC1–PC3) to each group identified 30 cocrystal-likely and 3 no-cocrystal pairs as named exceptions, listed with their dominant deviating descriptor in the Supplementary Materials (Table S7). The most prominent cocrystal-likely outliers are the three API–phloretin pairs (high molecular weight), the PIC and INIC pairs with the apolar aromatic coformers naphthalene, biphenyl, fluorene and hexamethylbenzene, together with NIC–hexamethylbenzene (functional-group class; these coformers carry no donor or acceptor group at all), the API–1,4-dioxane pairs (very low sp^2^ fraction) and the API–citric acid pairs (high rotatable-bond count). Among the no-cocrystal group, all three API–riboflavin pairs stand out, driven by the unusually high hydrogen-bond donor count of riboflavin. Overall, the cocrystal-likely coformers were well grouped around their centroid, whereas the no-cocrystal coformers define a shifted centroid, particularly along PC1 and PC2, indicating that both components capture, in complementary ways, descriptor contributions associated with unfavorable or less balanced API–coformer combinations. PCA can thus distinguish, at least partially, between predicted compatible and incompatible systems on the basis of the chosen molecular descriptor patterns.

**Figure 6:**
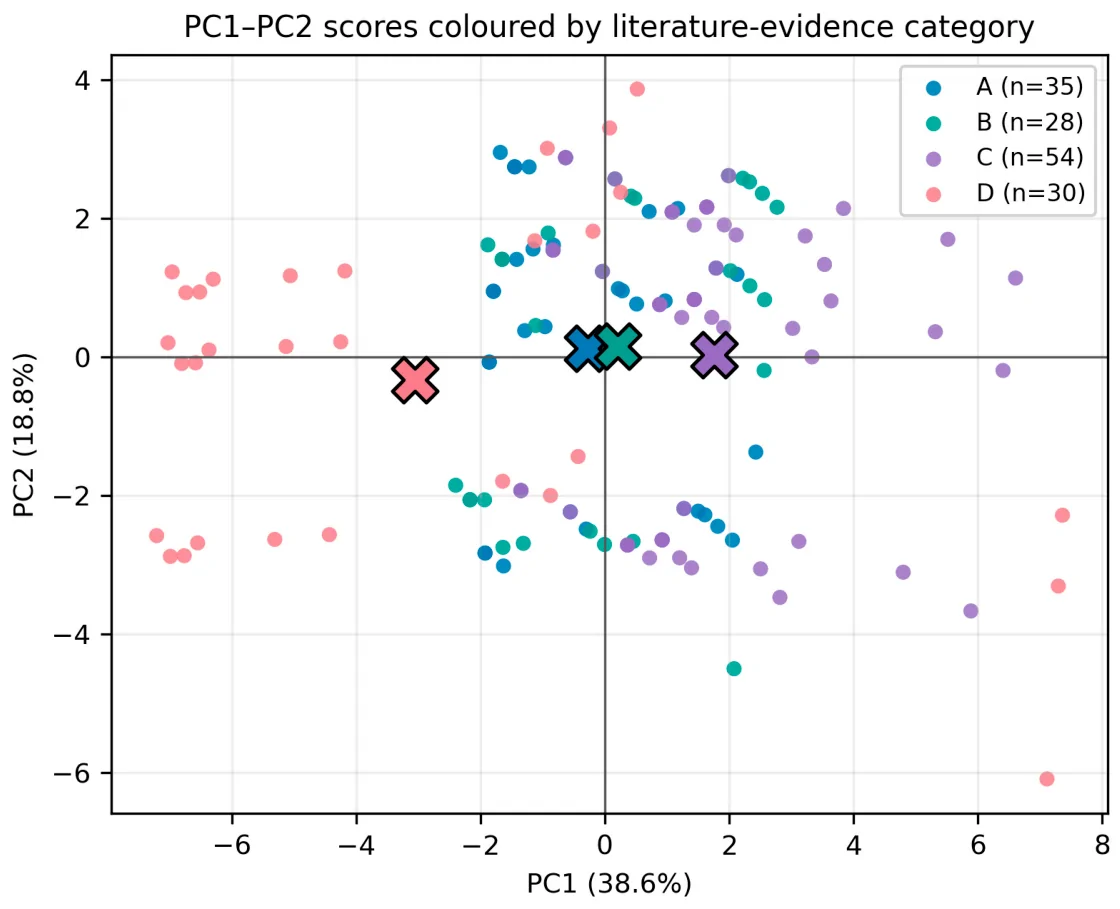
PC1–PC2 score plot colored by literature/rule evidence category. Groups A and B lie close to the origin (centroids at PC1 = −0.27 and +0.21), group C is displaced toward the positive end of PC1 (+1.76) and group D toward the negative end (−3.06), so the evidence categories are ordered along the polarity and complementarity axis.

When the same PCA space is colored according to evidence category (Figure 6), candidates belonging to groups A and B (directly reported and analogue-supported coformers, respectively) lie closest to the origin of the score plot (mean distances from the origin in the PC1–PC2 plane of 2.3 and 2.7 units, the two lowest of the four categories), indicating that experimentally known and literature-supported cocrystals occupy the region associated with balanced API–coformer properties. Candidates belonging to group C (rule-based probable coformers) are displaced toward the positive end of PC1 (centroid +1.76 against −0.27 and +0.21 for groups A and B), but their distributions overlap those of groups A and B over most of their range, so the rule-based selection strategy identifies coformers of the same general character, shifted toward higher polarity and hydrogen-bonding capacity (see also Table 12). In contrast, the low-probability candidates (group D) are the most widely scattered of the four categories (mean distance from the origin 5.4 units, standard deviation along PC1 of 4.4) and include several outliers at extreme PC1 and PC2 values, consistent with poor hydrogen-bonding capacity, excessive hydrophobicity, excessive molecular size or strong acid–base behavior that may favor salt formation rather than neutral cocrystallization.

**Figure 7:**
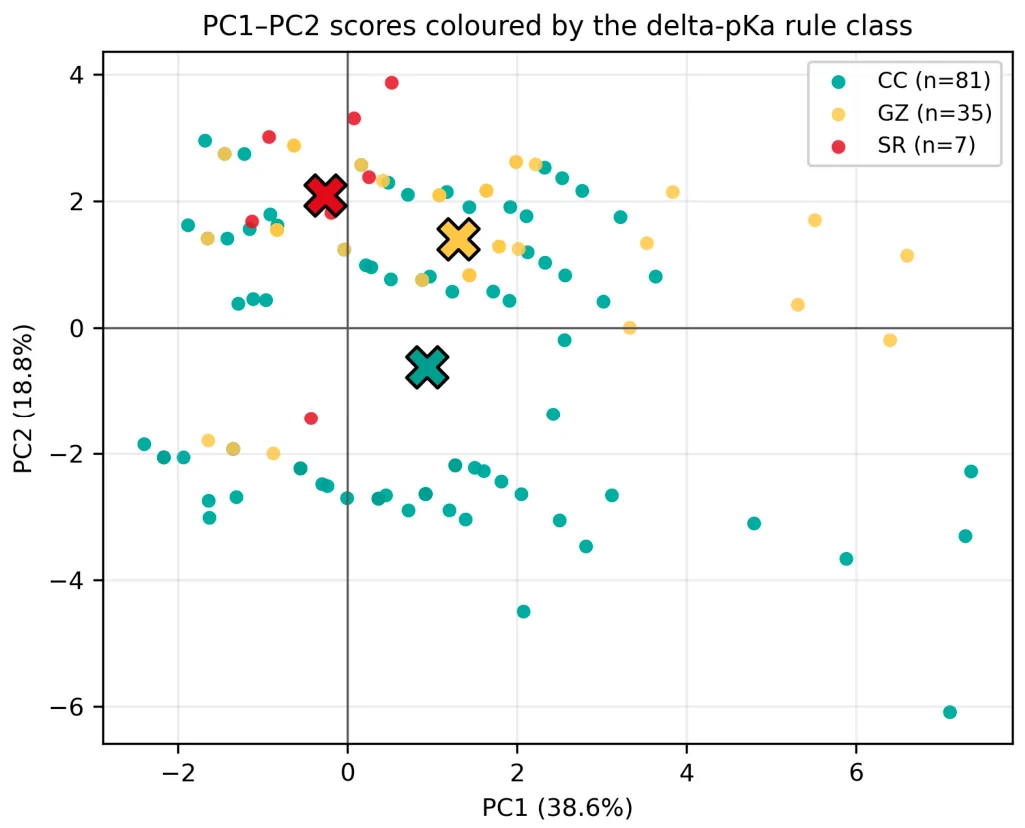
PC1–PC2 score plot colored by the pK_a_-rule post-filtering class (CC, cocrystal-compatible; GZ, gray zone; SR, salt risk; n.a., not applicable). Only the 123 pairs to which the rule applies are shown, the 24 pairs with a non-ionizable coformer being omitted; this set coincides in size with, but is not the same as, the 123 pairs predicted cocrystal-likely in Table 10. Salt-risk pairs (strong acids) and gray-zone pairs populate characteristic descriptor regions.

Re-coloring the same projection by the pK_a_-rule post-filtering class (Figure 7) provides a further external check. The salt-favored (SR) pairs, which involve the strongest acids (sulfonic acids and trifluoroacetic acid), are pushed toward elevated PC2 and toward the negative end of PC3 rather than along PC1, exactly where the acid–pyridine heterosynthon descriptors are largest, whereas the cocrystal-favored (CC) and gray-zone (GZ) pairs populate the central region. The corresponding centroids are listed in Table 13.

How cleanly each labeling separates along the leading components is quantified in Table 14, which reports the fraction of variance along each component that lies between groups. The API labeling separates almost entirely along PC2, which carries the API-specific geometry terms (83.7% between-group variance), whereas the prediction, class and evidence labelings are expressed mainly along PC1 (up to 33.1% for the evidence category). This confirms that the polarity/complementarity axis is the one the deep model effectively tracks.

### 2.3. Quantitative Assessment of Group Separation

The between-group variance shares reported in Table 14 are computed one principal component at a time and therefore quantify only how well a labeling is aligned with each individual axis; they do not, by themselves, establish that the groups form compact, non-overlapping clusters in the joint score space. To place any claim of “meaningful separation” on a quantitative footing, two standard cluster-validity indices were computed on the joint PC1–PC3 embedding of the 147 pairs, using each of the five labelings as the candidate partition: the silhouette score (sklearn.metrics.silhouette_score, mean over all pairs; range −1 to +1, with values near zero indicating overlapping groups and values approaching +1 indicating compact, well-separated groups) and the Davies–Bouldin index (sklearn.metrics.davies_bouldin_score; lower values indicate better-separated groups, with 0 as the theoretical minimum). Groups of fewer than two members are excluded from these calculations, since neither index is defined for singleton groups. The resulting values are collected in Table 15.

**Figure 8:**
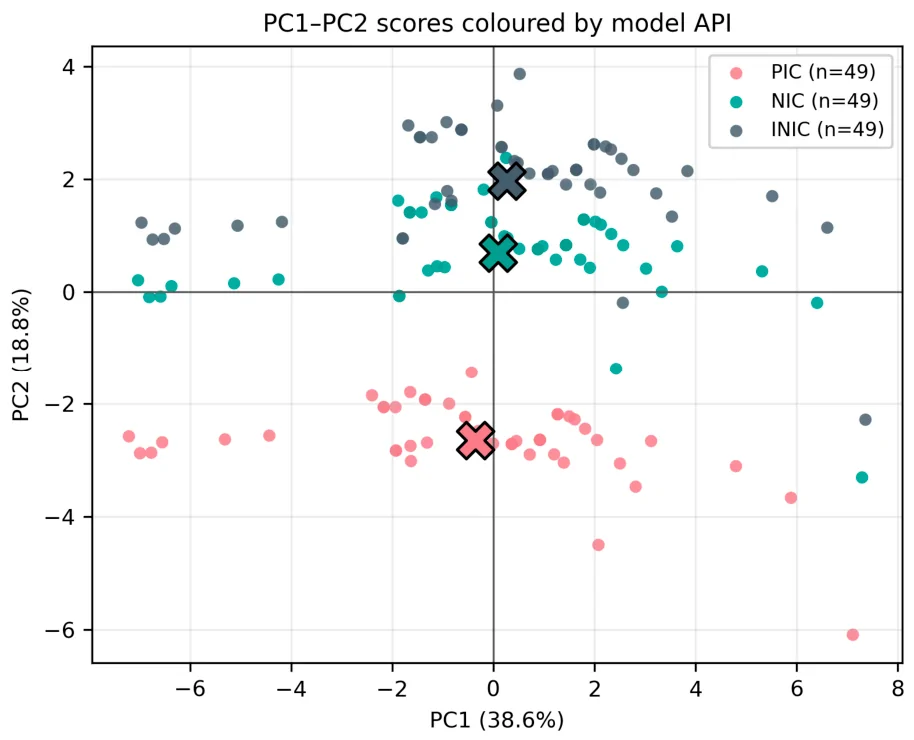
PC1–PC2 score plot colored by model API. Circles are individual API–coformer pairs and the bold crosses are the class centroids. The three scaffolds occupy largely overlapping regions because the descriptor variance is dominated by coformer chemistry; the systematic offset of the centroids along PC2 reflects the API-specific geometry/accessibility descriptors.

None of the labelings reaches the silhouette values (typically above 0.5) conventionally associated with strong, well-separated clustering, which is the expected outcome: the coformer library was not designed to form discrete clusters, and the score plots in the Supplementary Materials (Figures S1–S6) already showed substantial overlap between the groups. Of the five labelings tested, the binary *DeepCocrystal* prediction attains the most favorable combination (the highest silhouette score of 0.145 and the second-lowest (second-best) Davies–Bouldin index of 3.378), and the API-scaffold labeling is second on the silhouette score and third on the Davies–Bouldin index, consistent with the systematic, if modest, scaffold offset documented in Table 16, Figure 8 and Figure S5. The pK_a_-rule labeling attains the lowest (best) Davies–Bouldin index of all (2.678) but combines it with the lowest silhouette score (−0.020), a pattern that indicates groups that are internally tight relative to their spread but not well isolated from one another; the four-level *DeepCocrystal*-class and literature-evidence labelings are the least separated on both measures. Because the silhouette and Davies–Bouldin indices evaluate the joint PC1–PC3 embedding rather than the single best-aligned axis interrogated in Table 14, a labeling that separates cleanly along one component (as, for example, the *DeepCocrystal* prediction and class do along PC1) can still show modest values here when the other retained components add within-group scatter. These indices should therefore be read as confirming that the descriptor space organizes the pairs along interpretable chemical gradients rather than into sharply delineated clusters; they complement, rather than replace, the centroid and between-group variance evidence presented above.

Figure 8 shows the distribution of PIC, NIC and INIC cocrystal coformer candidates across the descriptor space (see also Table 16). The API-based PCA distribution showed that the three coformer clouds occupy largely overlapping regions, which is expected because the model APIs are closely related structurally; nevertheless, subtle systematic differences in their distribution are observed, reflecting the influence of pyridine–nitrogen position and amide orientation on supramolecular recognition.

For PIC, PCA separation appears to be strongly influenced by coformers capable of providing multiple hydrogen-bonding contacts, particularly hydroxy acids, phenolic compounds and dicarboxylic acids. Consistently, PIC occupies the most negative PC2 position of the three APIs (centroid PC2 = −2.65), because its API-specific descriptors take the lowest values: the pyridine nitrogen is the least accessible, and both amide donor and acceptor have the lowest accessibility scores, lowering the acid–pyridine and acid–amide heterosynthon propensities. The *ortho* relationship between the pyridine nitrogen and the carboxamide group restricts the simultaneous accessibility of both groups, making multipoint coformer interactions especially important for sufficient supramolecular reinforcement.

For NIC, the PCA distribution seems to reflect a balance between hydrogen-bonding capacity and aromatic/polarity descriptors, with candidates distributed across both central and moderately displaced regions, suggesting broad but descriptor-sensitive compatibility through acid–pyridine, acid–amide and amide–amide motifs. The NIC centroid occupies an intermediate PC2 position (+0.69), between PIC (−2.65) and INIC (+1.96), a direct consequence of the intermediate *meta* geometry, which provides intermediate pyridine–nitrogen accessibility and heterosynthon propensity.

For INIC, PCA suggests that geometric compatibility and acid–pyridine complementarity dominate, with candidates clustering within regions associated with dicarboxylic acids, aromatic acids and hydrogen-bond-rich coformers, in agreement with the most positive PC2 centroid (+1.96). This supports the conclusion that INIC is the most consistently favorable scaffold among the three APIs, because the *para* relationship between the pyridine nitrogen and carboxamide group maximizes the accessibility of both the pyridine–nitrogen acceptor and the amide donor/acceptor set, yielding the highest heterosynthon propensities of the three isomers. Importantly, the three APIs share one and the same intrinsic coformer descriptor structure: when the API-specific descriptors are removed, the three independent per-API PCA models are numerically identical (PC1 = 47.2%, PC2 = 18.4%, PC3 = 13.8%, with coincident scree curves; see Section 2.5). The apparent PIC/NIC/INIC separation in Figure 8 is therefore not a difference in which coformer descriptors drive each scaffold, but a difference in where each scaffold sits along the API-specific geometry directions of the single common global model.

**Figure 9:**
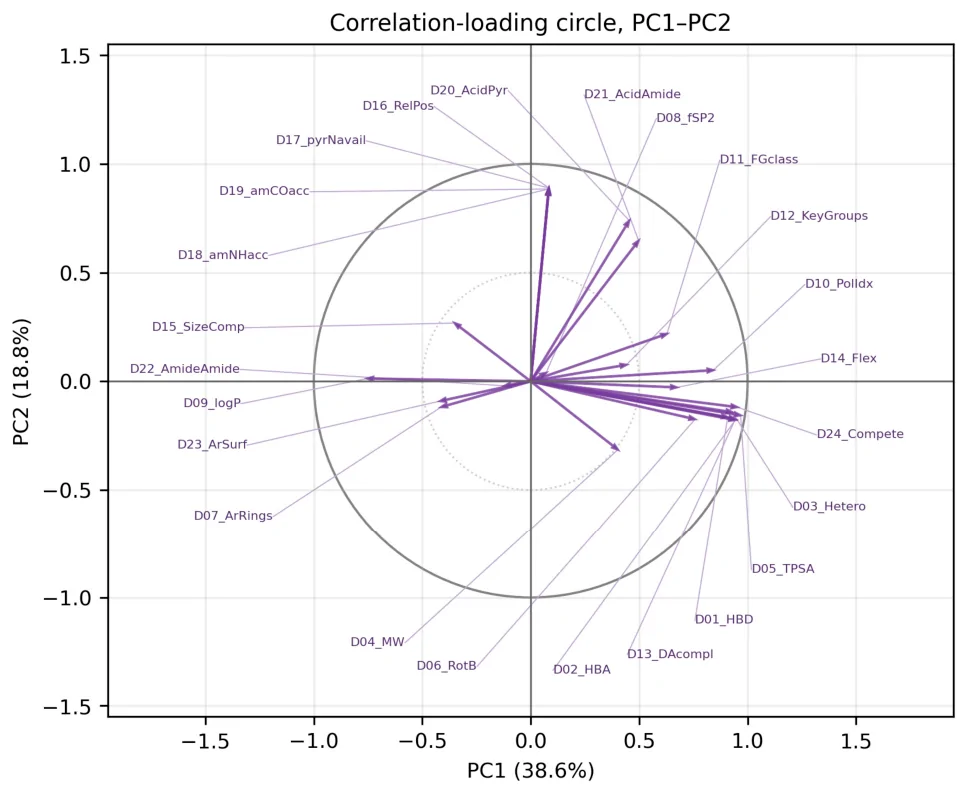
Correlation-loading circle for PC1–PC2. The position of each descriptor vector shows its correlation with the two components; small angles between vectors indicate mutually correlated descriptors.

**Figure 10:**
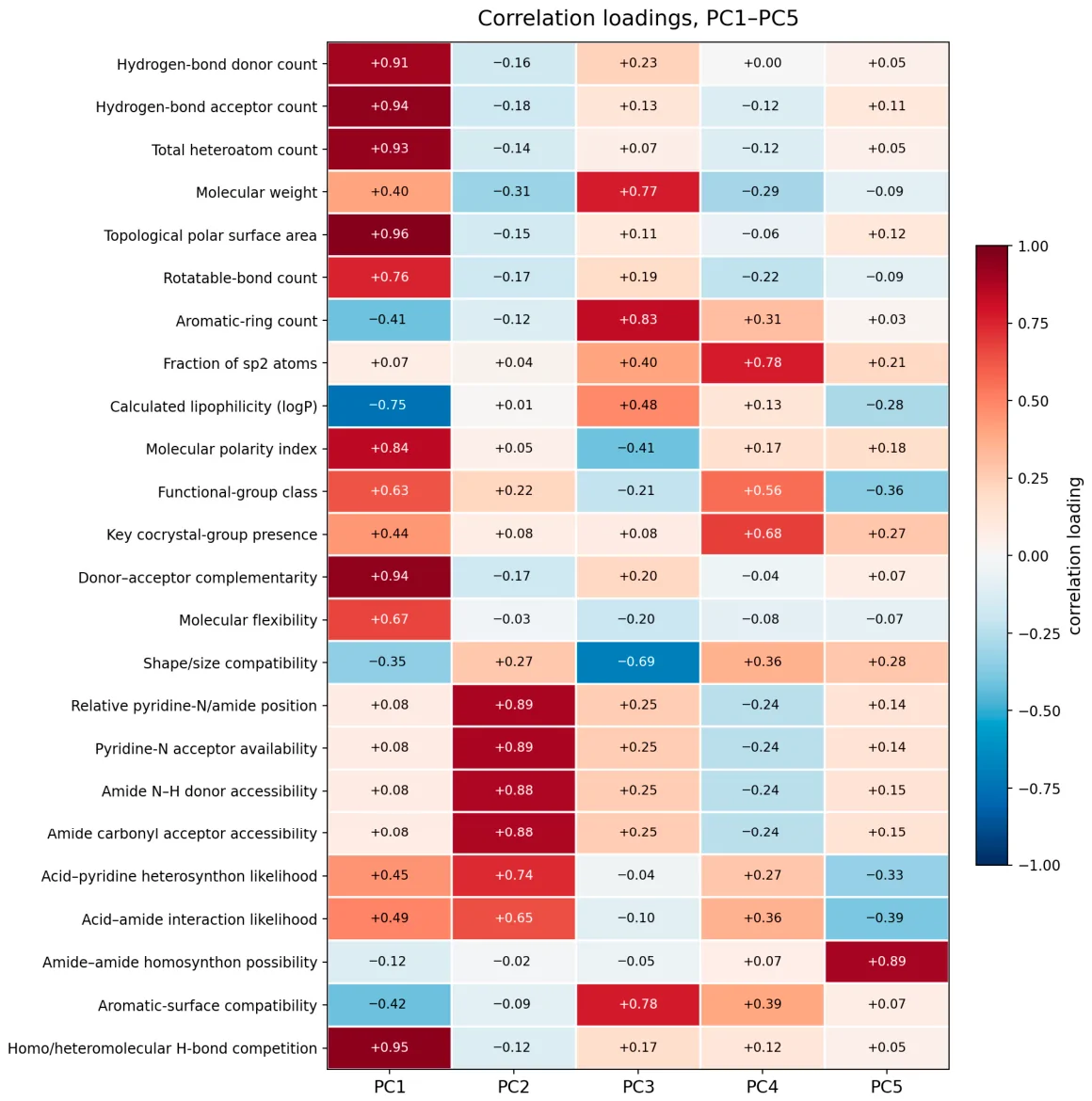
Heat map of the correlation loadings of all 24 descriptors on the first five principal components, giving the sign and magnitude with which each descriptor contributes to every component.

**Figure 11:**
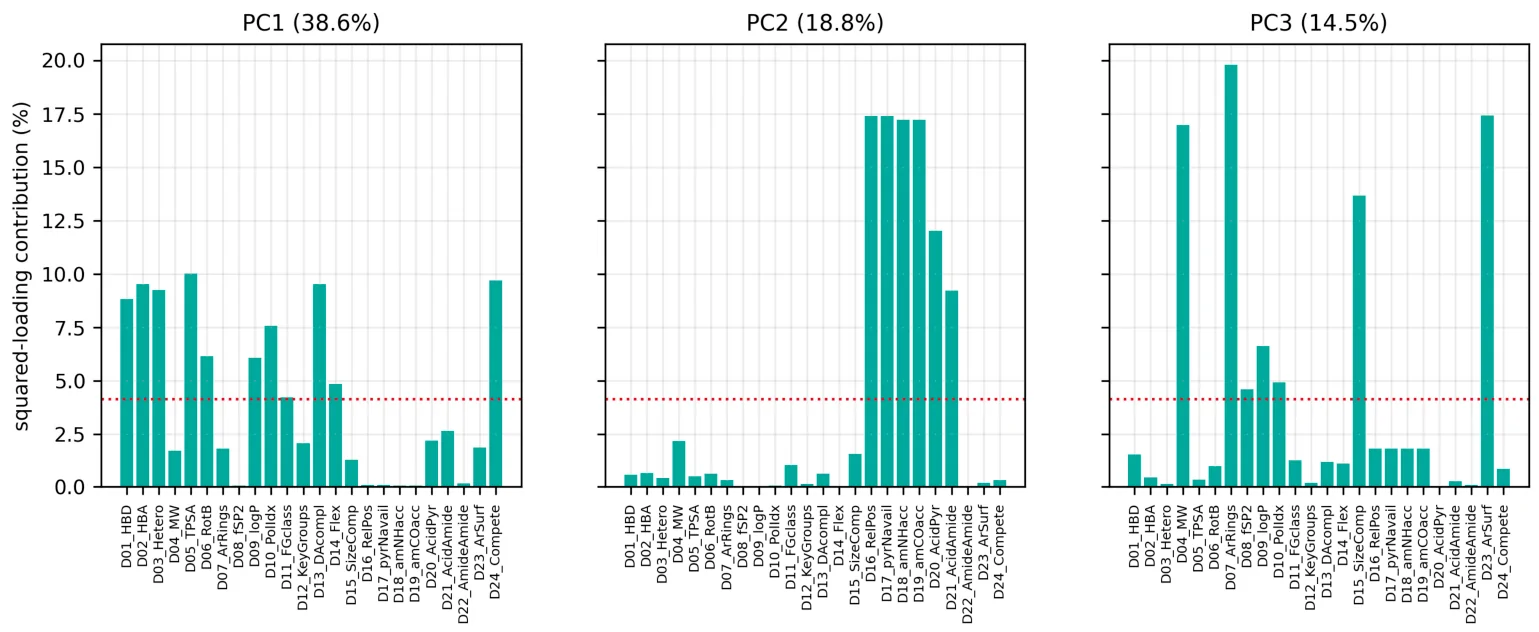
Squared-loading contributions (%) of each descriptor to PC1, PC2 and PC3. Descriptors above the dotted 1/24 reference line drive the corresponding component.

The interpretation of every score map rests on the loadings, which state how strongly each descriptor correlates with each component (Table 17, Figure 9, Figure 10, Figure 11 and Figure S3). The first principal component (PC1, 38.6% of the variance) is a polarity and hydrogen-bonding-capacity axis. Its leading positive contributors are topological polar surface area (+0.965), homo/heteromolecular hydrogen-bond competition (+0.949), donor–acceptor complementarity (+0.941), hydrogen-bond acceptor count (+0.940), total heteroatom count (+0.926), hydrogen-bond donor count (+0.905) and the molecular polarity index (+0.839), opposed by calculated lipophilicity (logP, −0.750) and aromatic-ring count (−0.412). The second component (PC2, 18.8%) is an API-geometry and heterosynthon-propensity axis, dominated by the four API-specific descriptors (pyridine-N availability, relative N/amide position, amide N–H donor and carbonyl acceptor accessibility, all near +0.89) together with the acid–pyridine (+0.736) and acid–amide (+0.645) heterosynthon terms. The third component (PC3, 14.5%) is an aromatic-surface and size axis, driven by aromatic-ring count (+0.829), aromatic-surface compatibility (+0.778) and molecular weight (+0.767), and opposed by shape/size compatibility with the API scaffold (−0.689).

### 2.4. Three-Dimensional Principal Component Analysis

Retaining the third component recovers an additional 14.5% of the variance and resolves structure that overlaps in the planar projection. The three-dimensional score plots are presented in the Supplementary Materials (Figures S4–S6). As static 3D representations cannot fully resolve the depth of individual points on a two-dimensional page, our quantitative interpretation is founded upon the centroid tables and 2D projections rather than upon direct visual inspection of the 3D distributions. The third axis principally spreads the pairs along the ionization/synthon-competition direction, so the salt-favored strong-acid coformers and the amide-bearing self-associating coformers, which can project close together on PC1–PC2, are pulled apart along PC3. The three-dimensional API view makes the scaffold displacement fully explicit: the para isomer (INIC) forms the most compact and most favorably placed cloud, the meta isomer (NIC) is intermediate, and the ortho isomer (PIC) is the most displaced toward the hindered, lower-complementarity region, mirroring the relative breadth of cocrystal compatibility reported for the three APIs.

### 2.5. Per-API Analysis

**Figure 12:**
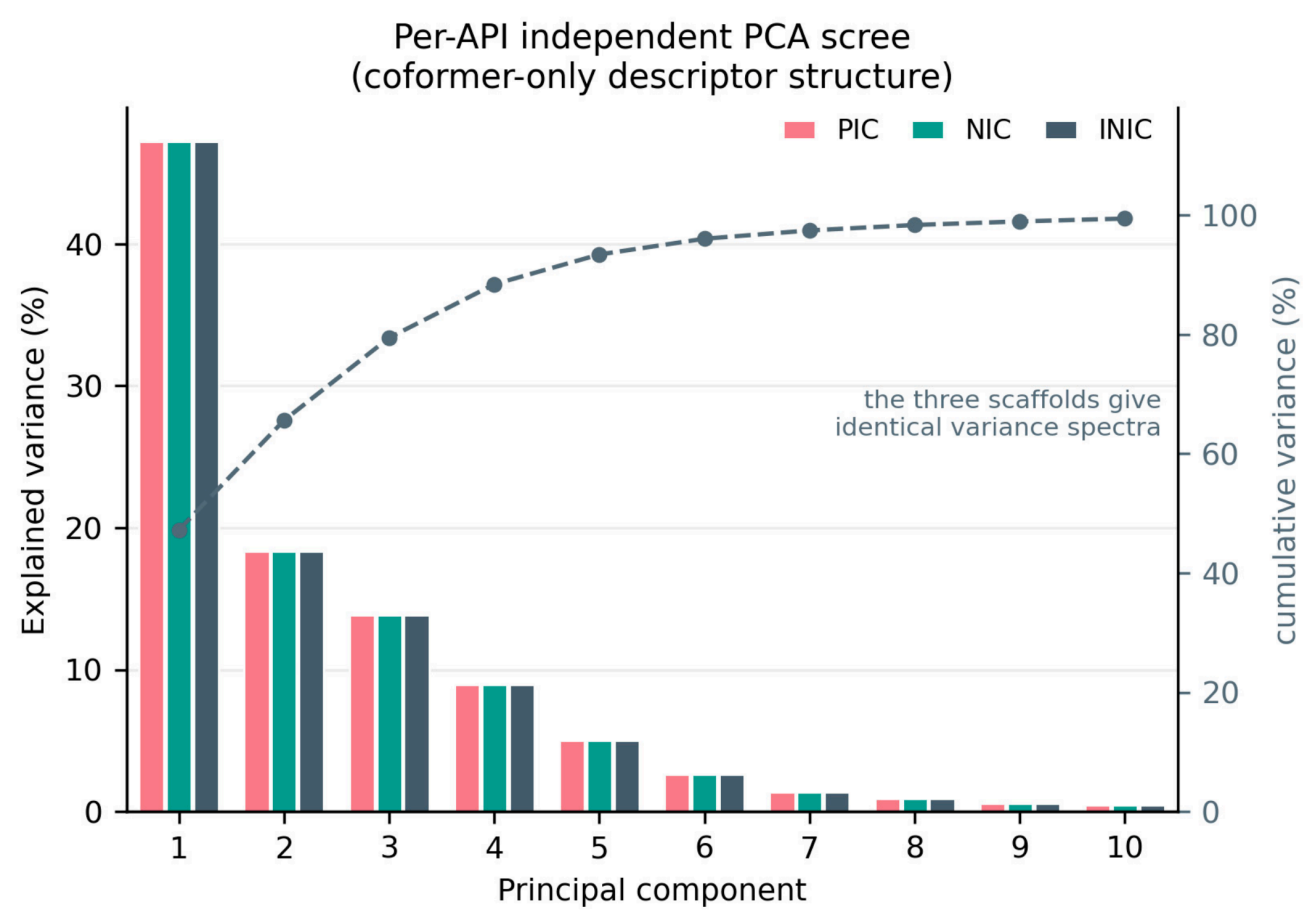
Explained variance of the three independent per-API PCA models, shown as grouped bars for each component, with the shared cumulative variance on the right axis. The bar heights coincide exactly: once the API-constant descriptors are removed, the residual variance is governed solely by coformer chemistry, so the three scaffolds share an identical intrinsic descriptor structure.

To establish whether the three APIs possess different intrinsic descriptor structures or merely occupy different regions of a common space, an independent PCA was computed for each scaffold after discarding the descriptors that are constant within that scaffold (the API-specific geometry terms), leaving the 20 coformer-driven descriptors. The result (Table 18, Figure S7 and Figure 12) is unambiguous: the three per-API models are numerically identical, each placing 47.2% of the variance on PC1, 18.4% on PC2 and 13.8% on PC3, with coincident scree curves. Within any single API, the API-specific descriptors are constant and contribute nothing to the within-API variance; the residual structure is governed entirely by coformer chemistry, which is the same library in all three cases. The three scaffolds therefore share one and the same intrinsic descriptor manifold, and what distinguishes them is not the shape of this manifold but their position along the API-specific directions of the global model. This resolves the earlier difficulty in seeing per-API trends in the global score plots: those trends are positional contrasts encoded by descriptors D16–D19, not scaffold-dependent differences in which descriptors drive the chemistry.

### 2.6. Chemical Insight from the Descriptor Map

A key question is what the descriptor map reveals beyond the established principles of hydrogen-bond complementarity and polarity matching in cocrystal formation. Using the picolinamide, nicotinamide, and isonicotinamide series as a case study, we identify three family-specific insights that qualitative synthon reasoning cannot provide.

First, the isomer ranking is geometrically determined. Once the four API-specific geometry descriptors (D16 to D19) are excluded, the three isomers share a numerically identical coformer manifold (Table 18, Figure S7 and Figure 12). Along the pyridine–nitrogen/amide-accessibility axis (PC2) the three scaffolds are ordered INIC (centroid +1.96) > NIC (+0.69) > PIC (−2.65) (Table 16), so what separates them is a rigid translation of the same coformer chemistry along that axis rather than any difference in preferred coformer features. The number of DeepCocrystal-positive pairs orders the isomers differently, INIC (44) > PIC (41) > NIC (38): the descriptor map and the model agree that isonicotinamide is the most favorable scaffold, but they interchange the other two. The accessibility axis therefore accounts for the position of the para isomer relative to the ortho isomer and not, by itself, for the model’s ranking of the meta isomer, whose three-pair margin over PIC out of forty-nine scored pairs is in any case too small to carry interpretation. The para isomer outperforms the ortho isomer because of steric accessibility: in PIC, the ortho nitrogen is shielded by the adjacent carboxamide, so the effect is steric rather than electronic. This distinction is left implicit by the raw *DeepCocrystal* scores.

Second, PC3 separates two failure modes that the 2D map conflates. Strong-acid coformers, which favor salt formation, and amide coformers, which promote homosynthon competition, project close together on PC1–PC2 but are resolved along PC3. The risk of failing to form a neutral cocrystal is thus not unidirectional: proton transfer and homosynthon competition are encoded on independent axes, so a coformer can pose one risk without the other.

Third, the seven pK_a_-flagged salt-risk pairs cluster at the high-heterosynthon-propensity end of PC2 (Table 13, Figure 7), overlapping with the highest *DeepCocrystal* scores. The descriptors that maximize cocrystal probability, namely dense hydrogen bonding, high polarity, and strong acid–pyridine complementarity, are inherently tied to proton-transfer risk. This explains why high scores alone cannot separate cocrystals from salts and justifies the pK_a_ filter as a necessary, non-redundant check. The two tools diverge precisely where their disagreement is most chemically informative. This coupling between score-maximizing and salt-risk descriptor vectors is reported here for the first time for pyridinecarboxamides, and it illustrates the value of mapping predictions rather than relying on raw scores.

## 3. Conclusions

The present study demonstrates that the combination of *DeepCocrystal* prediction, descriptor-based principal component analysis, and pK_a_-guided post-filtering provides a coherent and chemically interpretable framework for the computational screening of cocrystal systems. While *DeepCocrystal* efficiently identifies molecular pairs with potential cocrystallization propensity, the additional PCA analysis enables interpretation of the underlying descriptor space and reveals the structural factors most strongly associated with the predicted outcomes. It is important to emphasize that this descriptor-based layer serves only as an interpretive aid. Although it clarifies the rationale behind the scores assigned by *DeepCocrystal*, it is neither intended to improve, nor does it improve, the predictive accuracy, candidate ranking, or false-positive rate of the core model.

The PCA results showed that, for the case API model systems described in this study and the chosen set of coformers, the descriptor variance is dominated by three principal dimensions associated with (i) hydrogen-bonding capacity and molecular polarity, (ii) API-specific geometric accessibility and heterosynthon propensity, and (iii) aromatic surface and molecular-size effects. The analysis further demonstrated that predicted cocrystal-compatible and non-compatible systems occupy distinguishable regions of the descriptor space, supporting the physical and chemical relevance of the descriptors employed. In addition, the separation observed among the three pyridinecarboxamide isomers was shown to arise primarily from differences in the accessibility of the pyridine nitrogen and amide functionalities rather than from fundamental differences in the intrinsic coformer descriptor landscape. The descriptor map highlights a critical overlap between the score-maximizing trajectory and the salt-risk vector at the high-heterosynthon-propensity end of PC2. This structural convergence clarifies why a high *DeepCocrystal* score alone remains incapable of discriminating a cocrystal from a salt in such acid–base systems. The comparison with the literature evidence also delimits the applicability of the model: the six pairs in which one pyridinecarboxamide is offered as the coformer of another all receive negative scores, two of them with high confidence, although cocrystals of all three isomer combinations are reported. Homofunctional amide-on-amide pairs are therefore under-represented in the training domain of DeepCocrystal, and for this family the descriptor map, which places the same pairs in the region of viable heterosynthon formation, is the more reliable guide.

The pK_a_-based post-filtering procedure proved useful as a complementary filter to *DeepCocrystal* predictions, enabling the identification of systems where proton transfer may compete with neutral cocrystal formation. The results highlight the importance of considering acid–base relationships when interpreting AI-generated cocrystal predictions and demonstrate that a simple chemically grounded post-processing step can significantly improve the practical value of screening results.

More generally, the work illustrates how descriptor-level interpretation can be combined with modern deep-learning approaches to bridge predictive performance and chemical understanding. The methodology is readily transferable to other classes of pharmaceutical compounds and may contribute to the development of more transparent and rational strategies for coformer selection. Future developments will focus on the implementation of an automated screening platform integrating the present methodology with additional descriptors related to crystallization conditions and experimental outcomes, aiming at a more comprehensive and practically oriented prediction of multicomponent solid forms.

## Figures and Tables

**Table 1:** Selected set of coformers.

N	Coformer		N	Coformer	
1	Salicylic acid	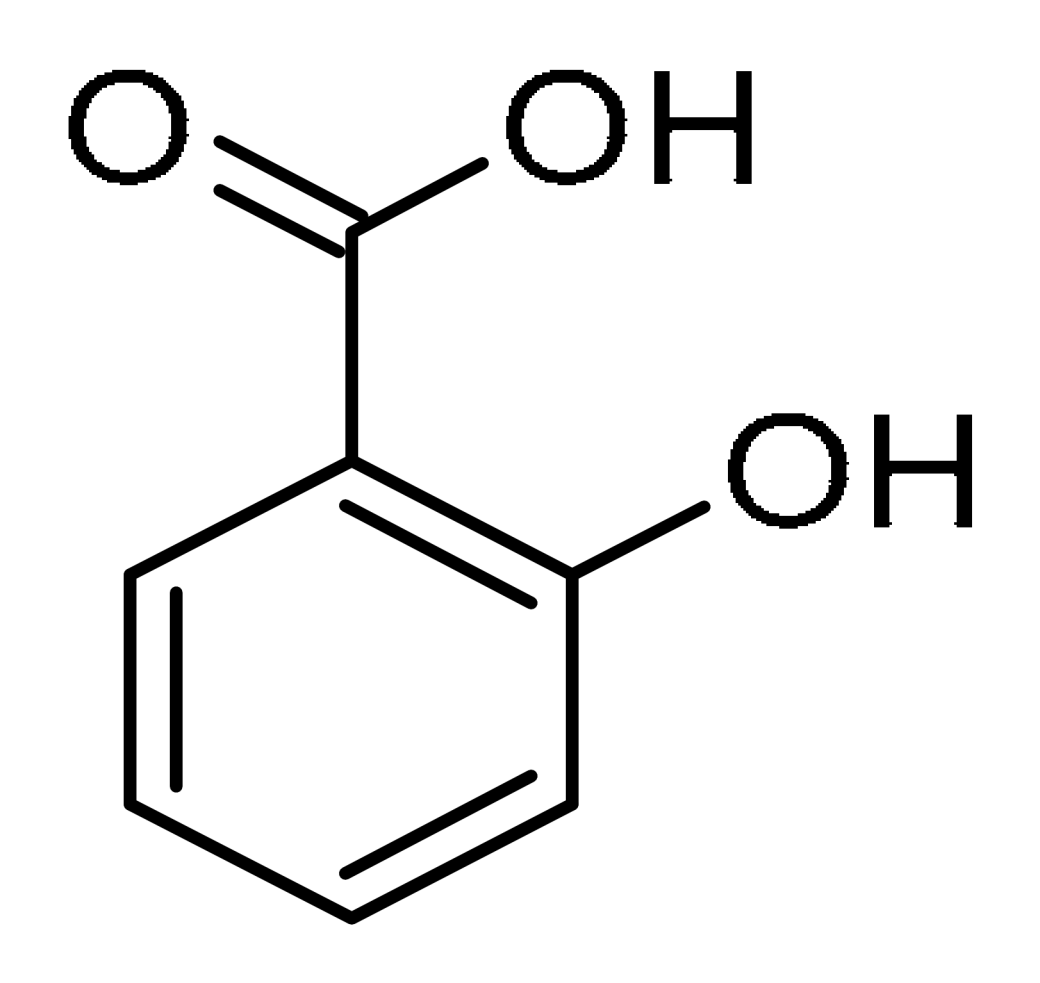	27	Ferulic acid	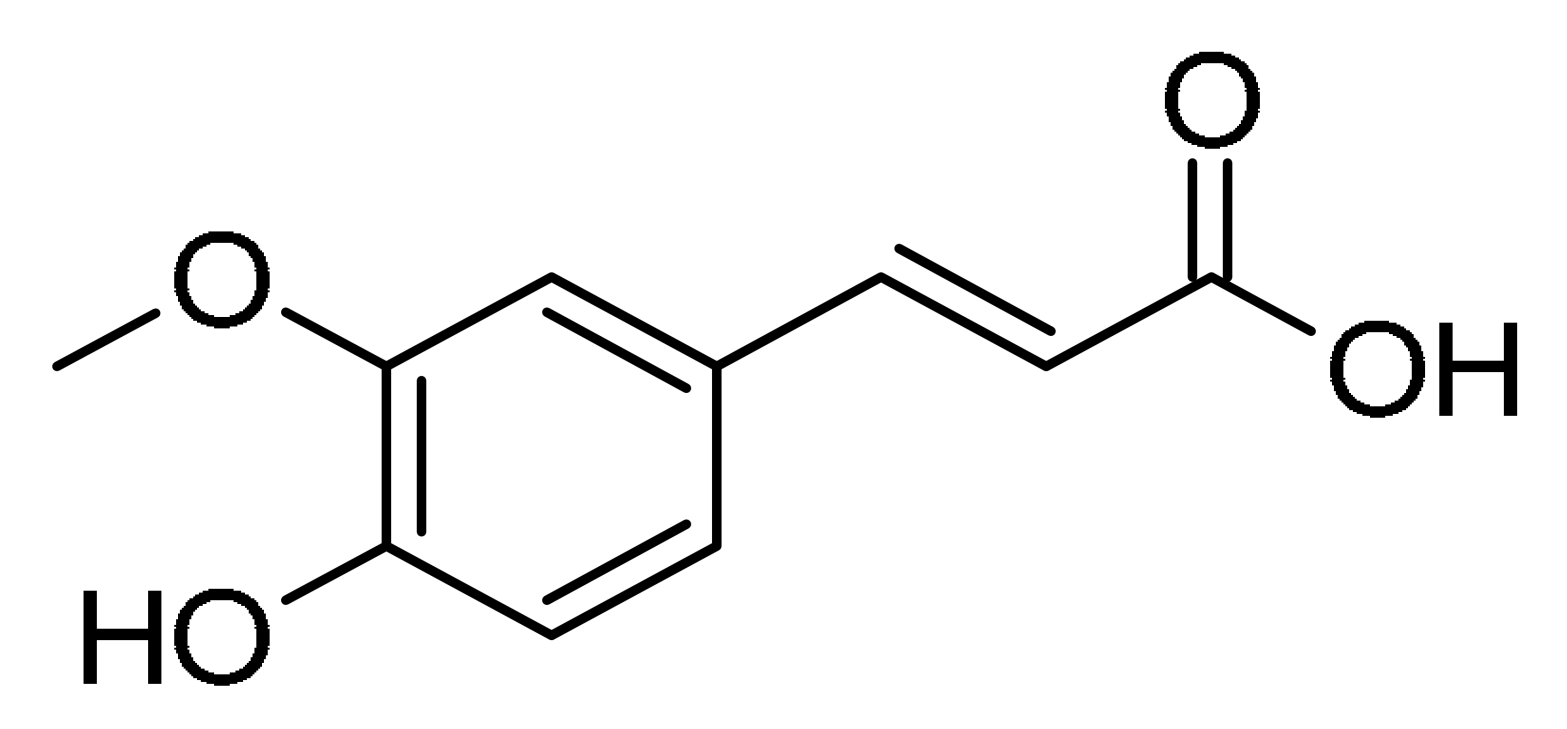
2	(S)-Mandelic acid	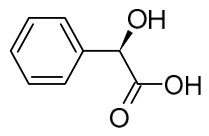	28	Syringic acid	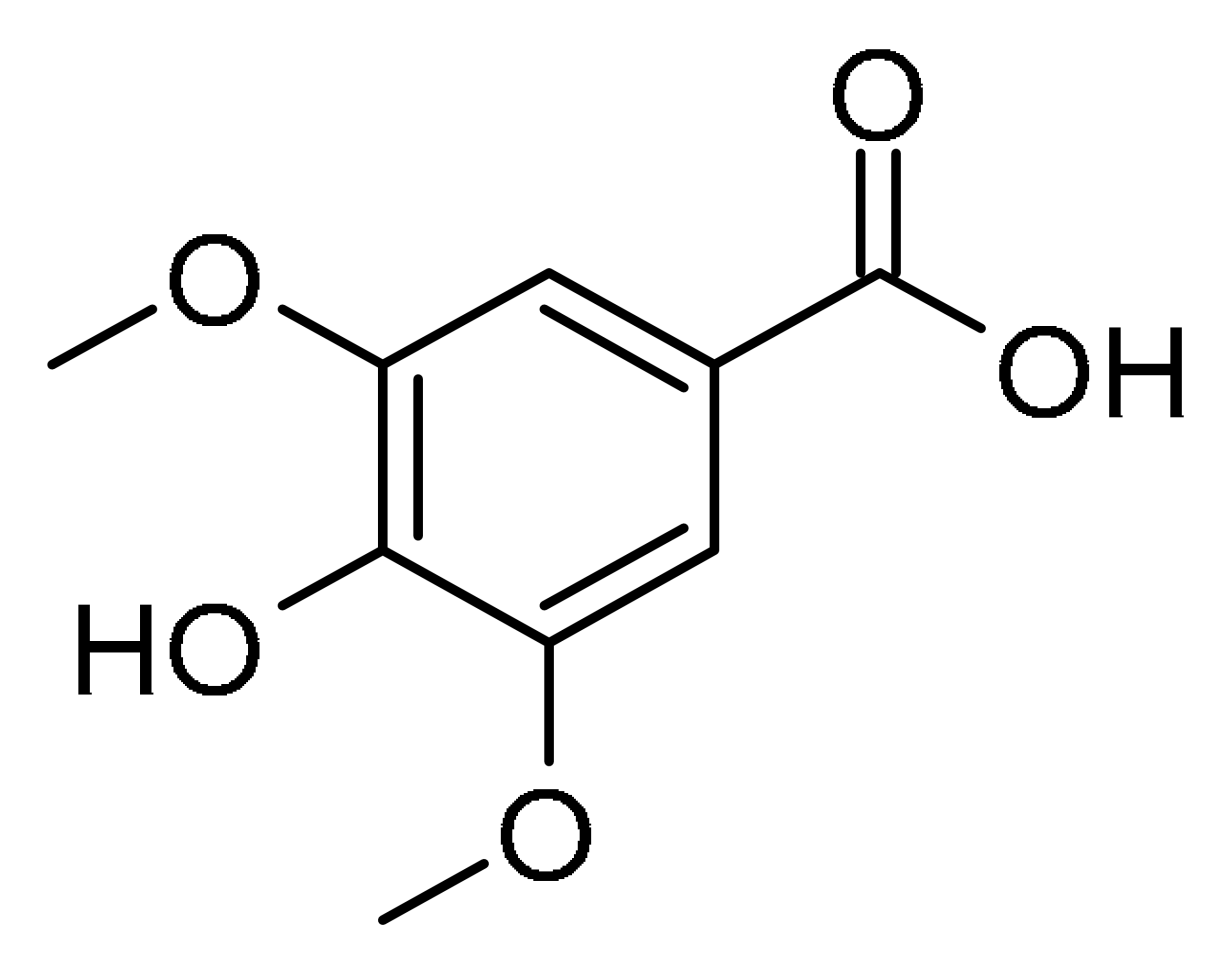
3	Malonic acid	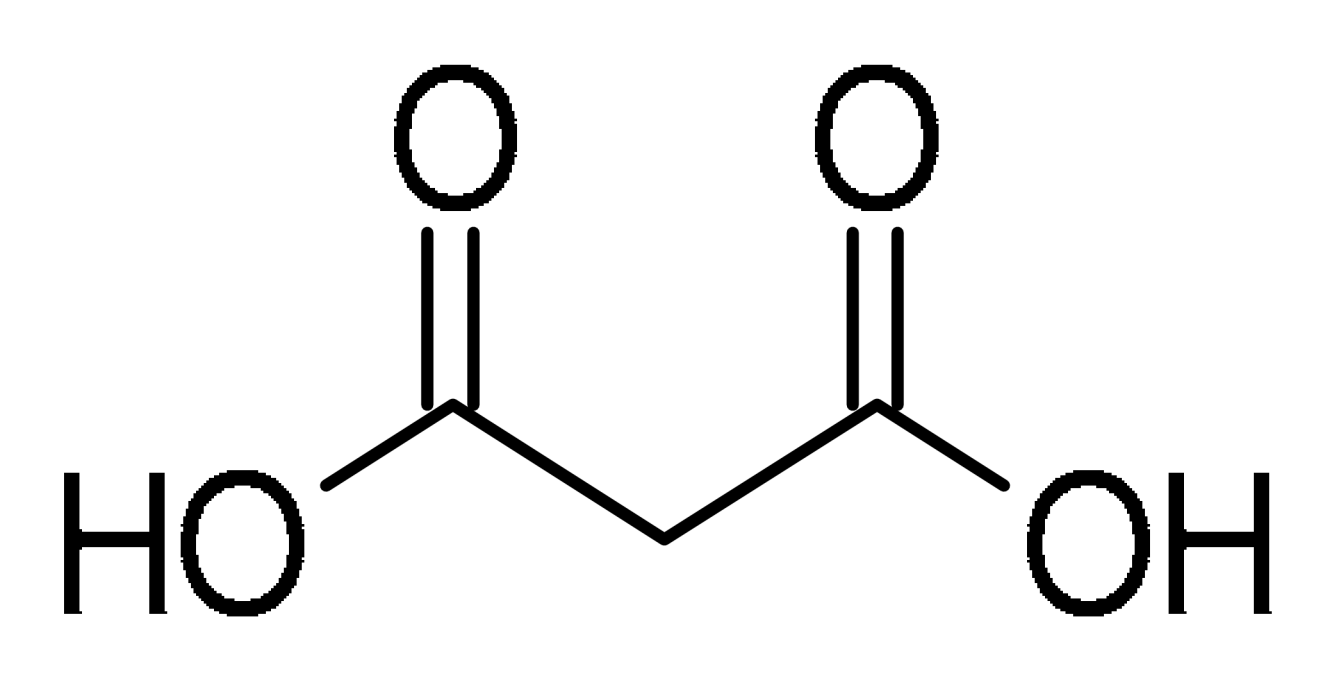	29	Protocatechuic acid	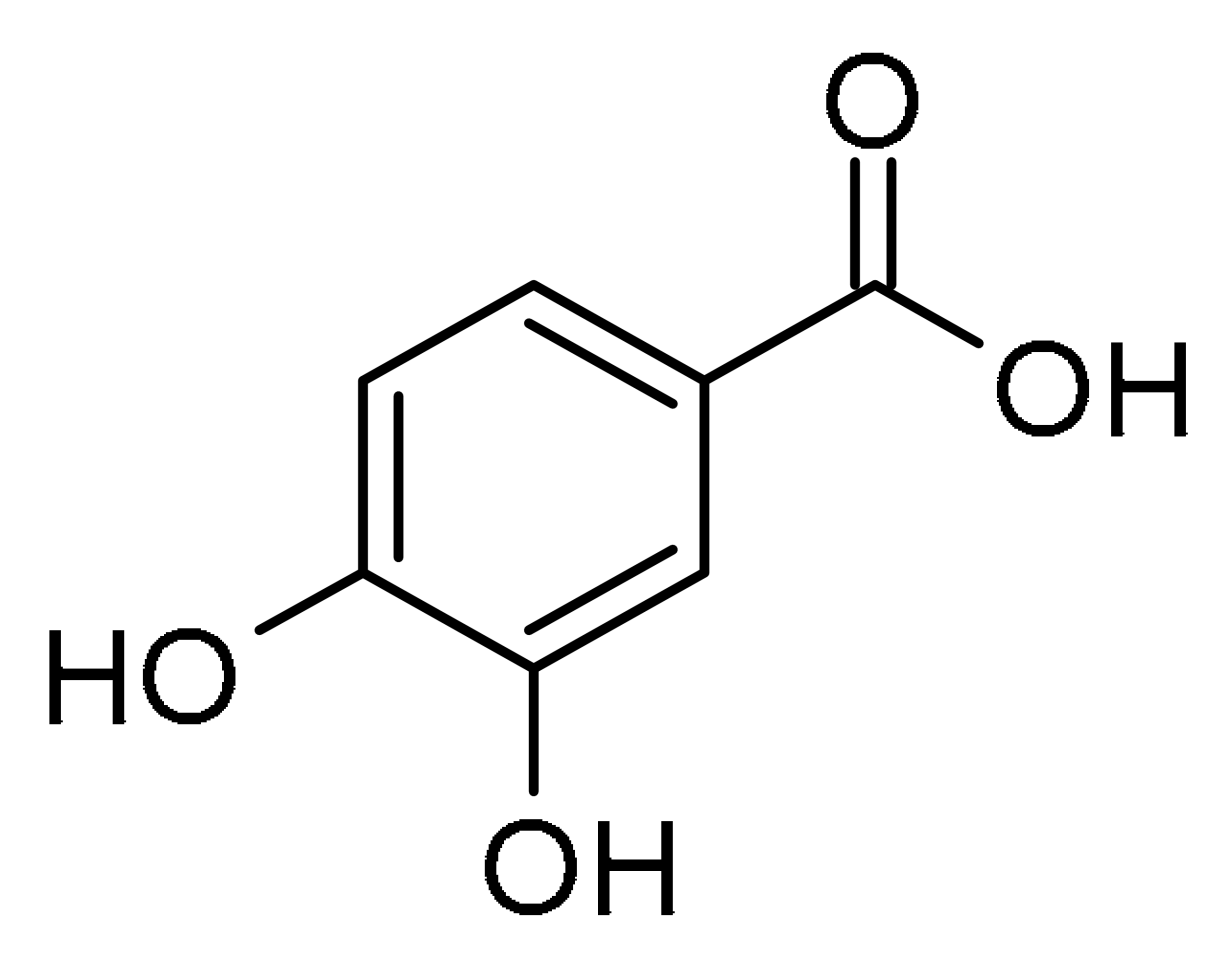
4	Succinic acid	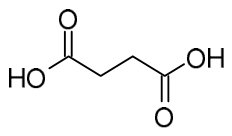	30	2,5-Dihydroxybenzoic acid	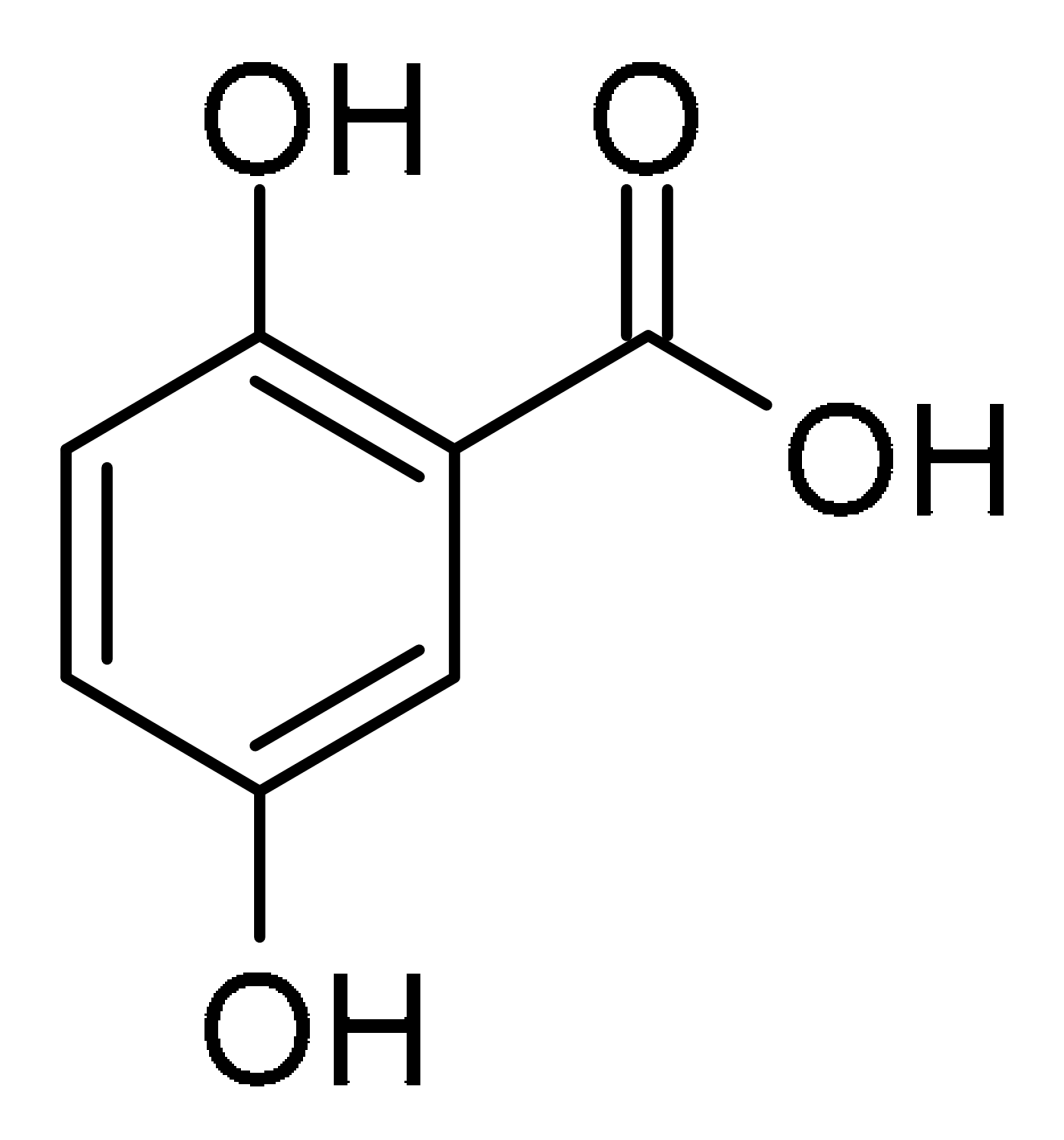
5	Fumaric acid	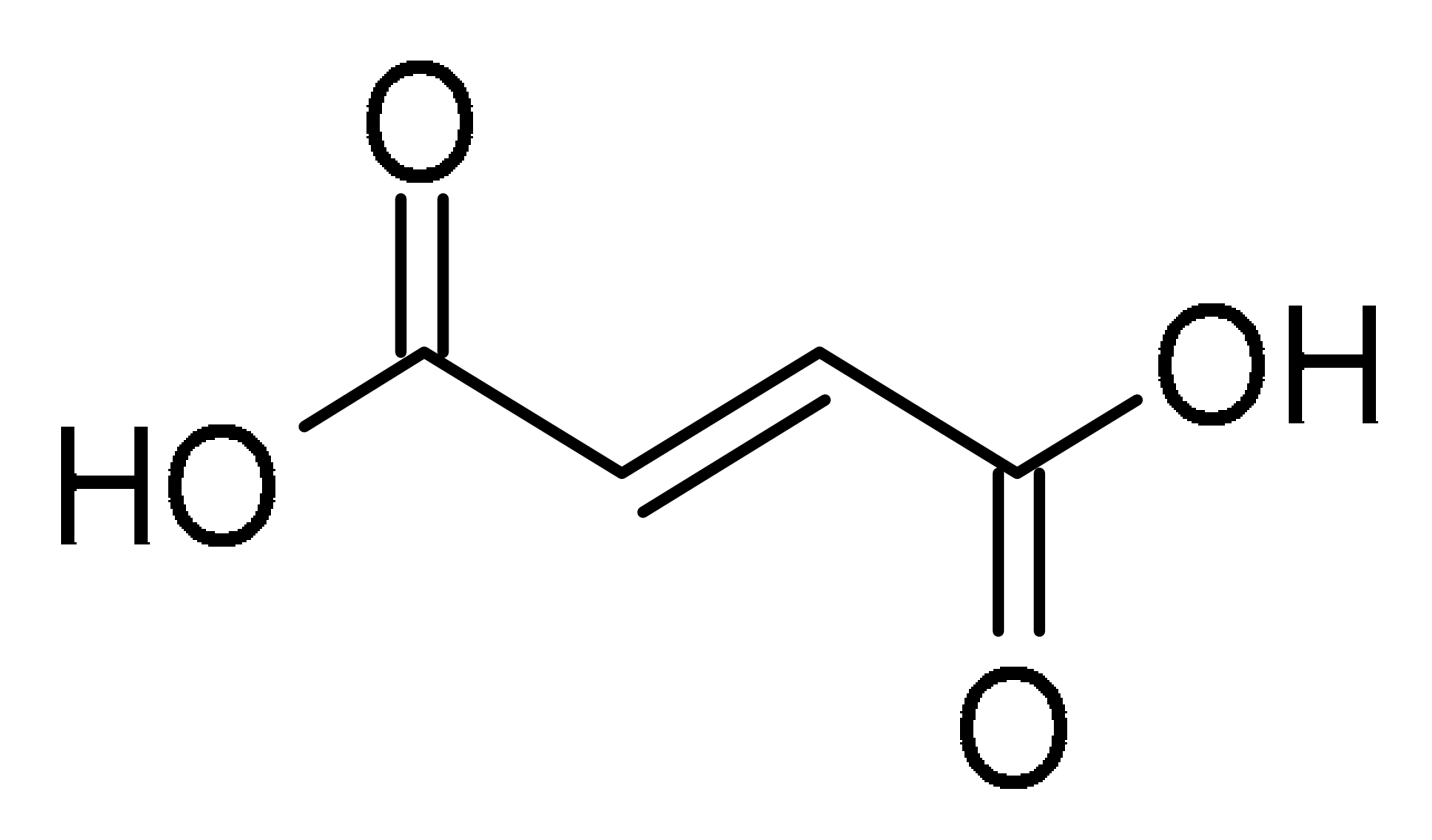	31	2,6-Dihydroxybenzoic acid	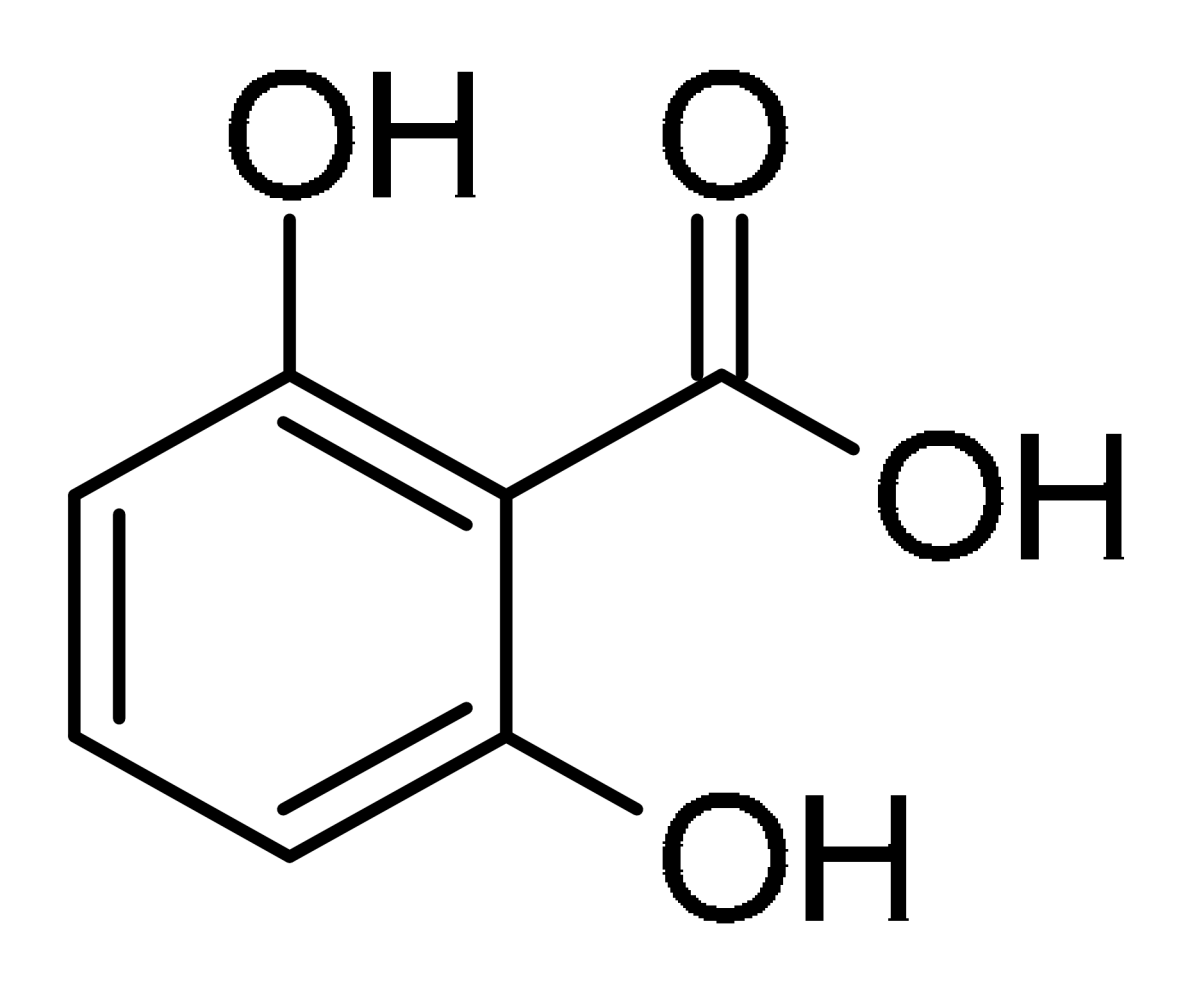
6	Glutaric acid	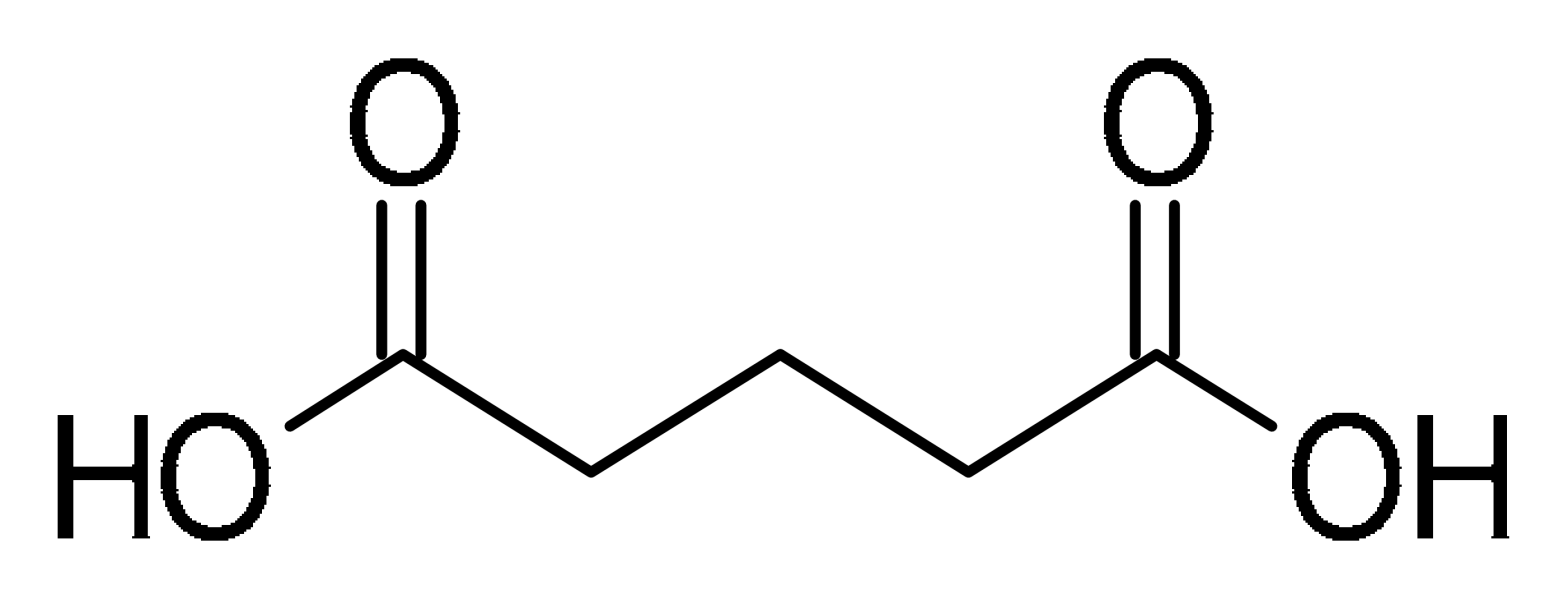	32	4-Hydroxybenzoic acid	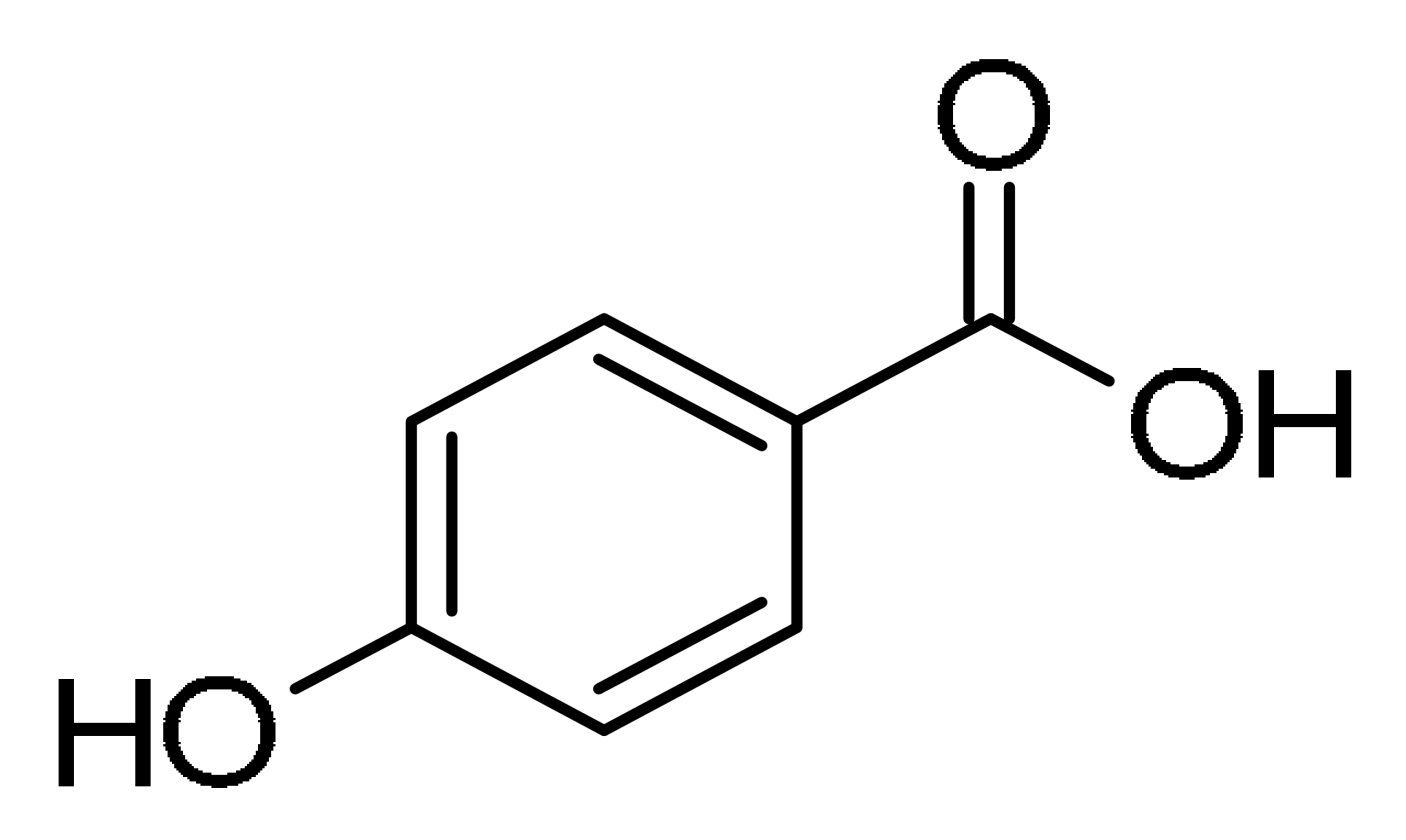
7	Adipic acid	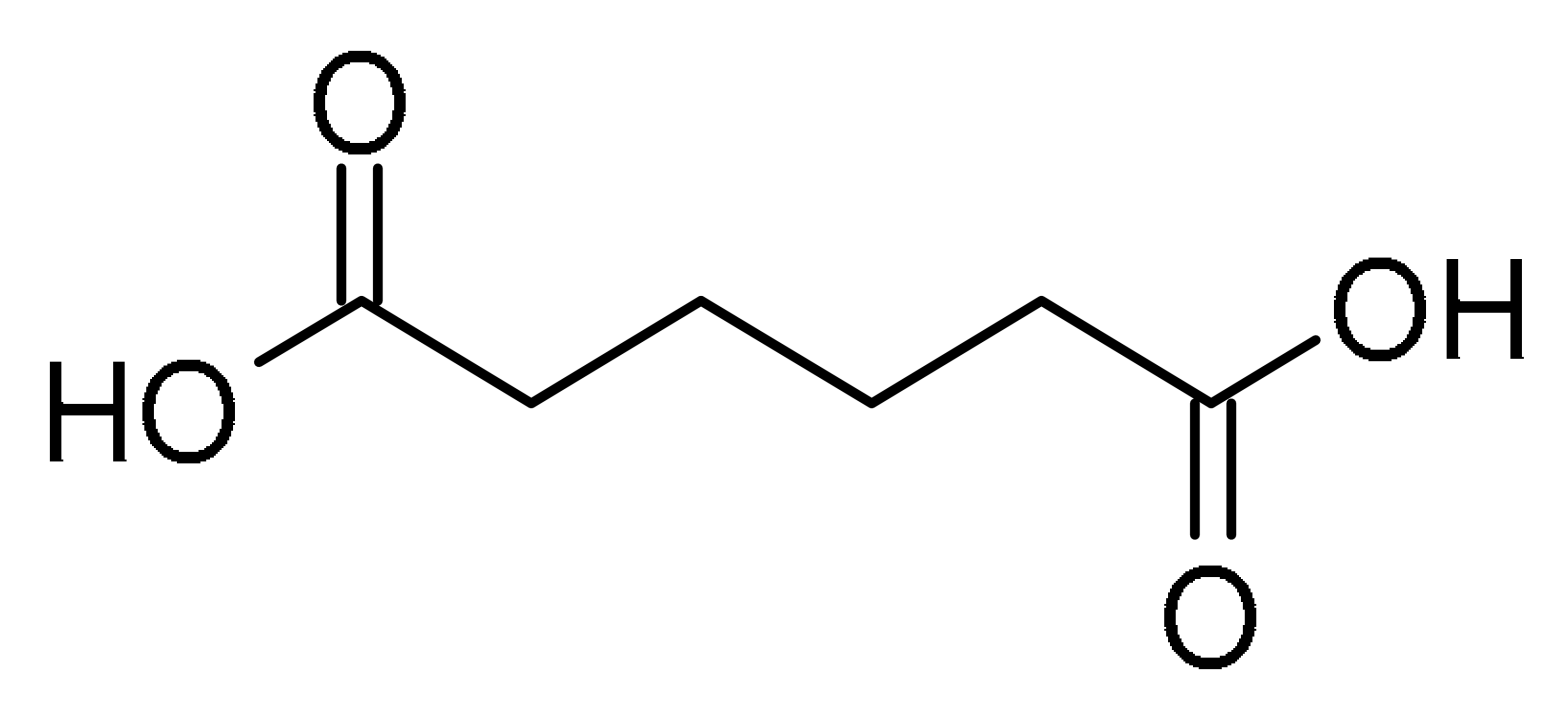	33	Nicotinic acid	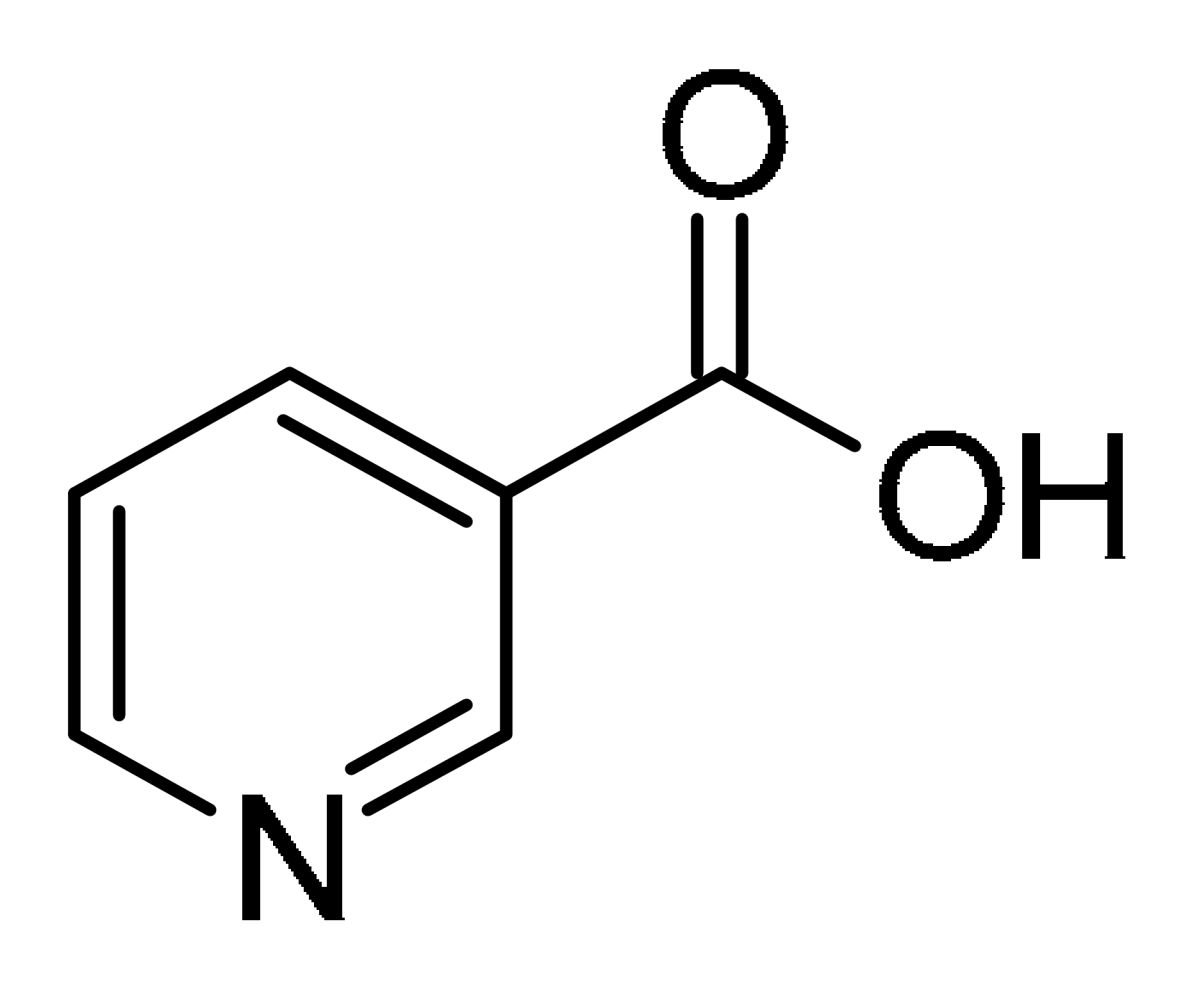
8	Picolinic acid	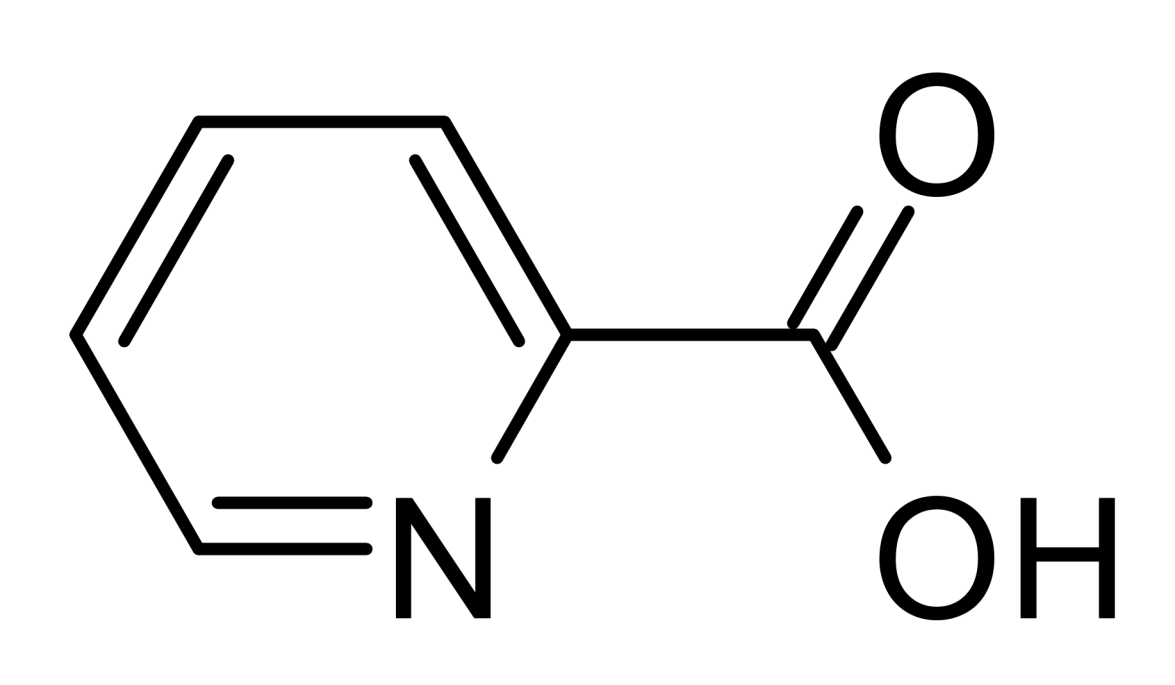	34	Isonicotinic acid	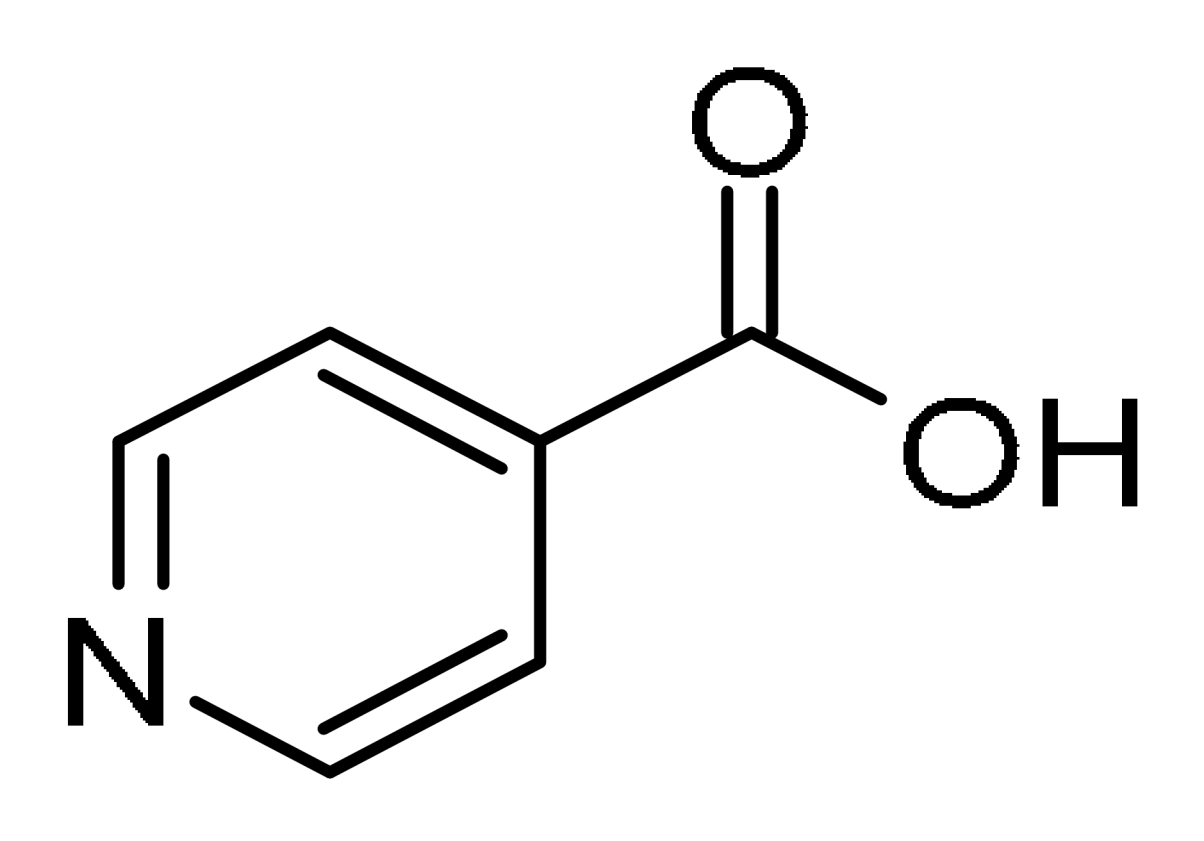
9	Naproxen	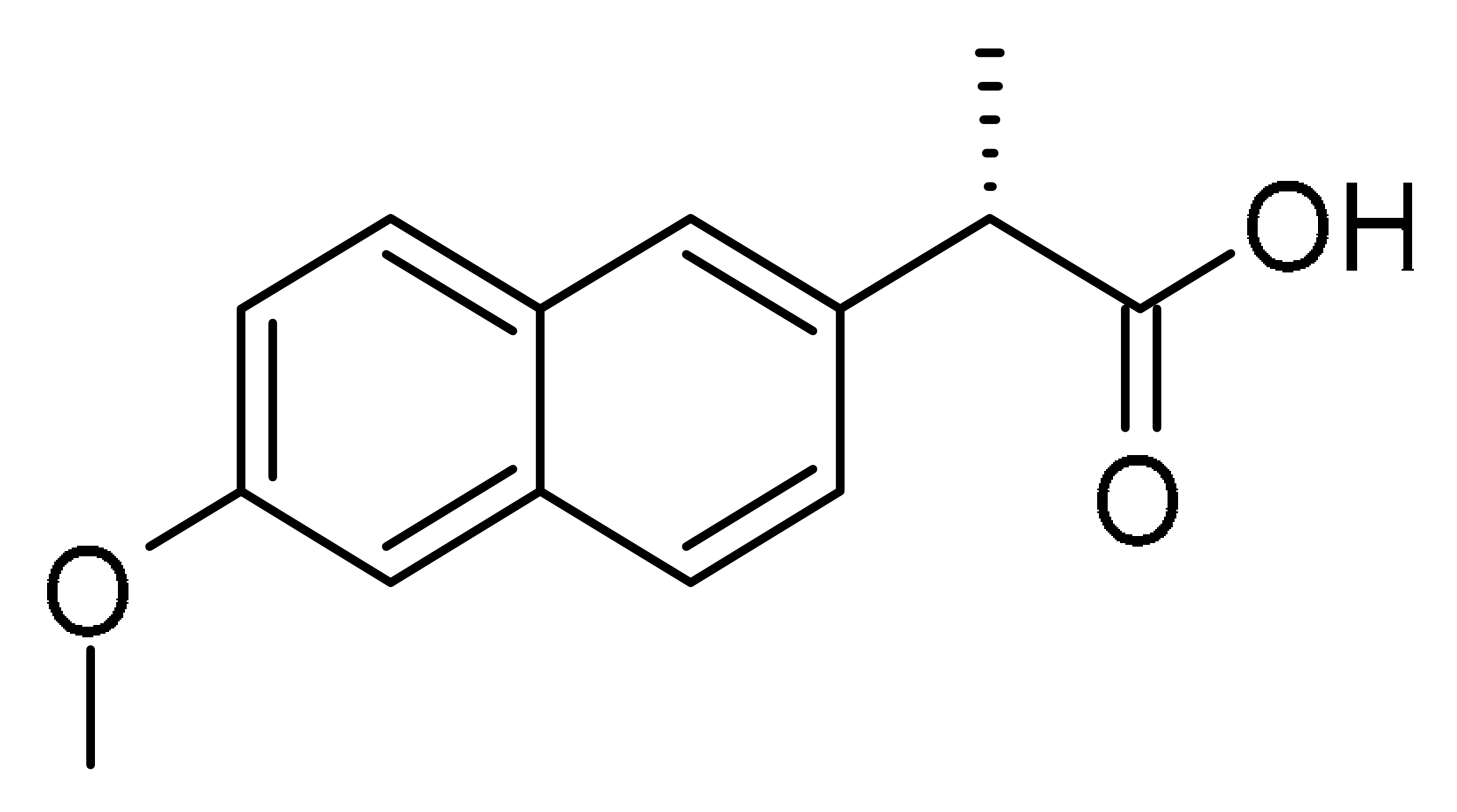	35	Maleic acid	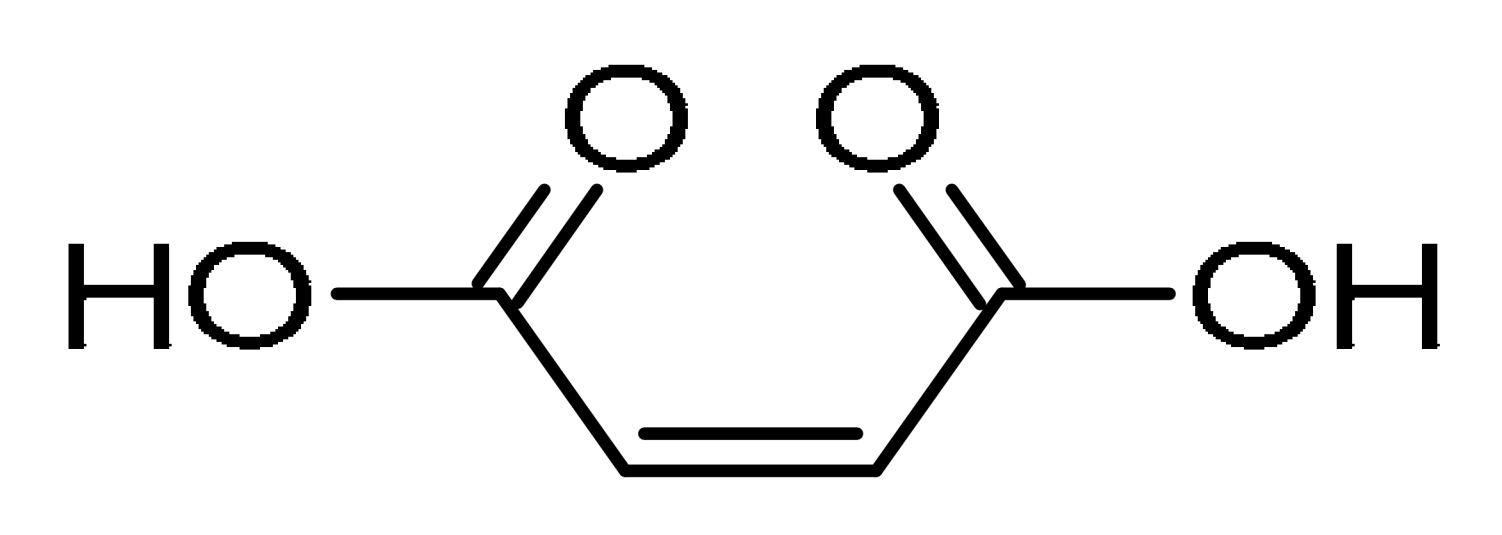
10	Vanillin	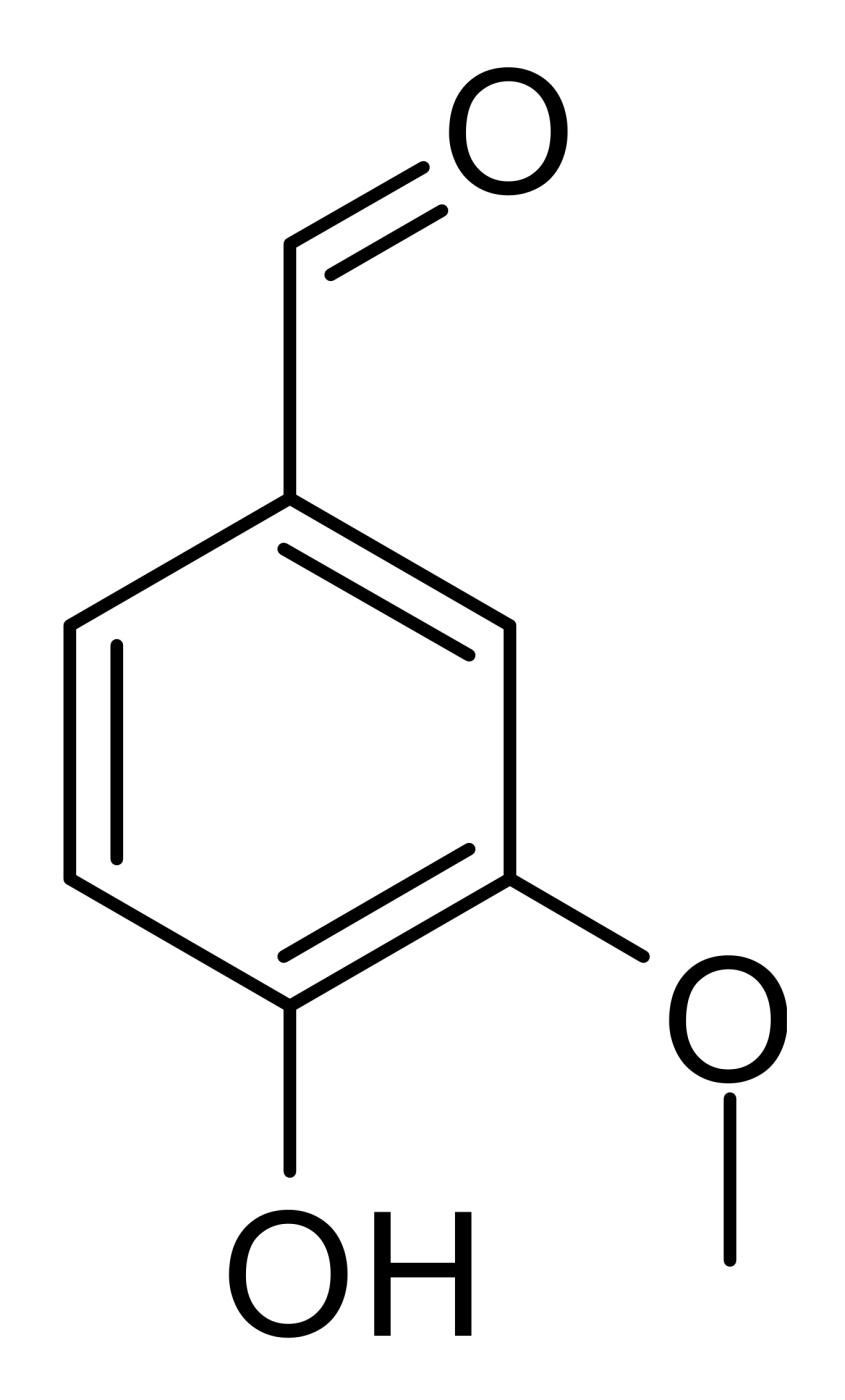	36	Malic acid	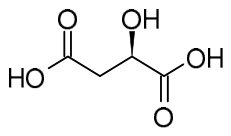
11	Vanillic acid	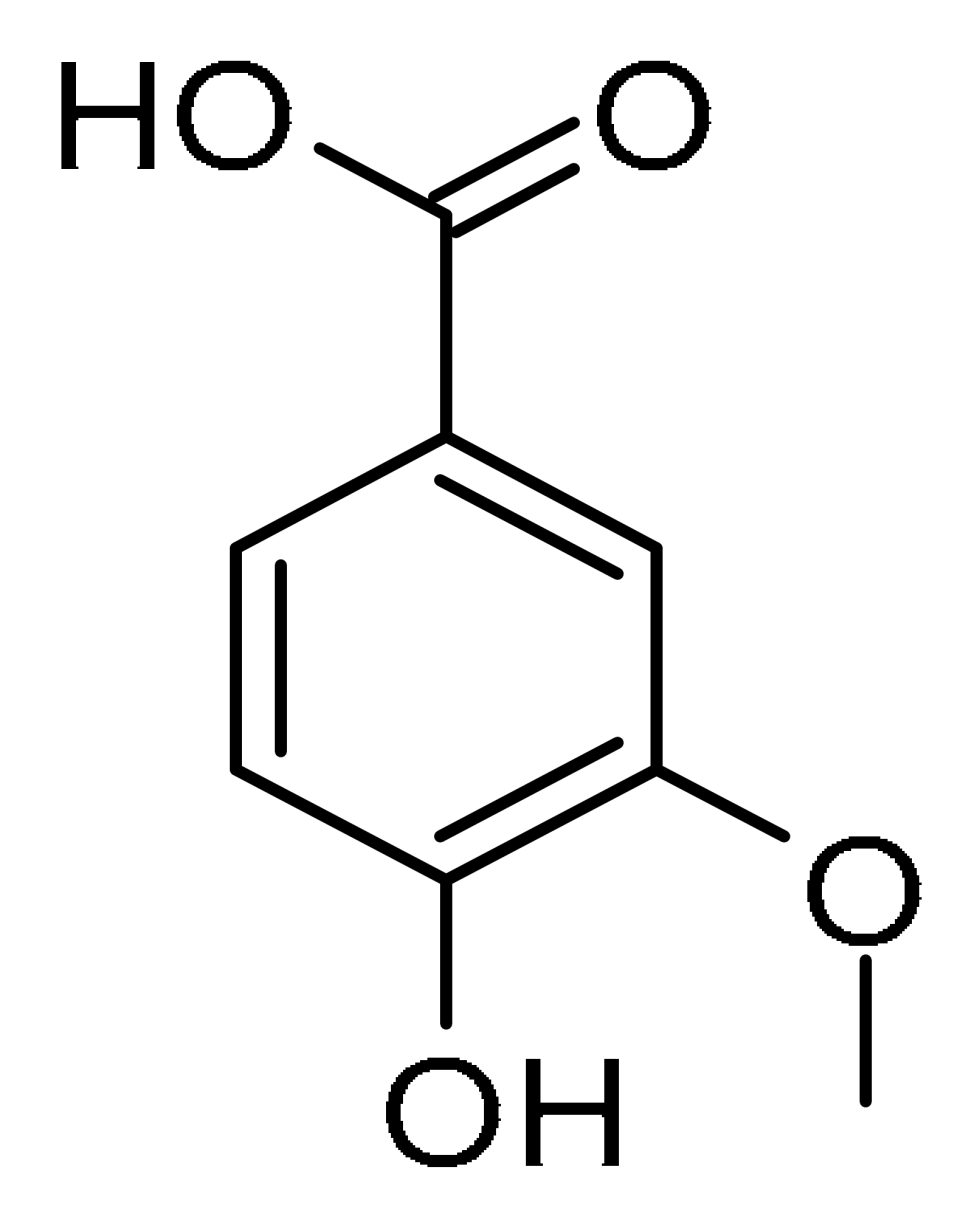	37	Tartaric acid	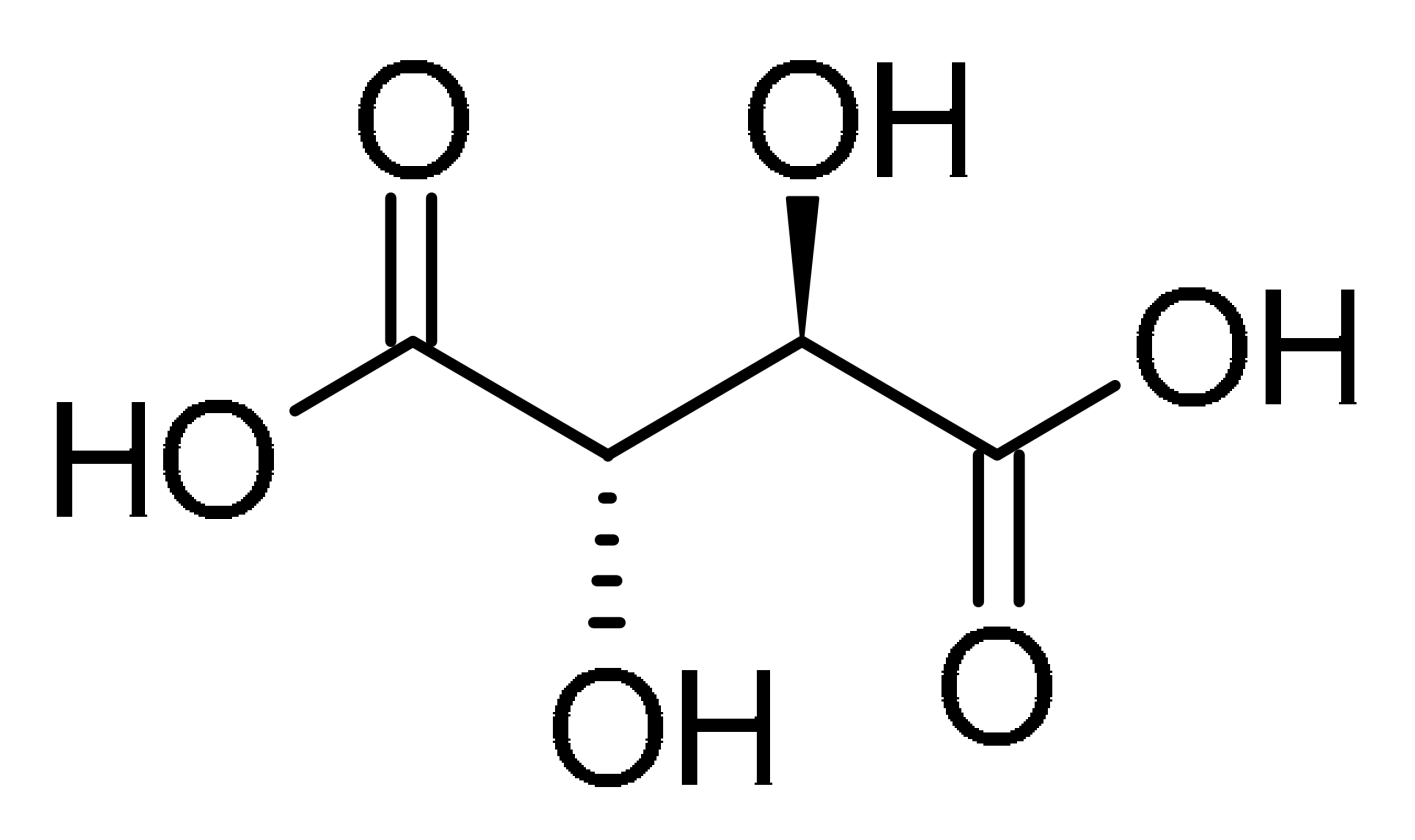
12	Phenylacetic acid	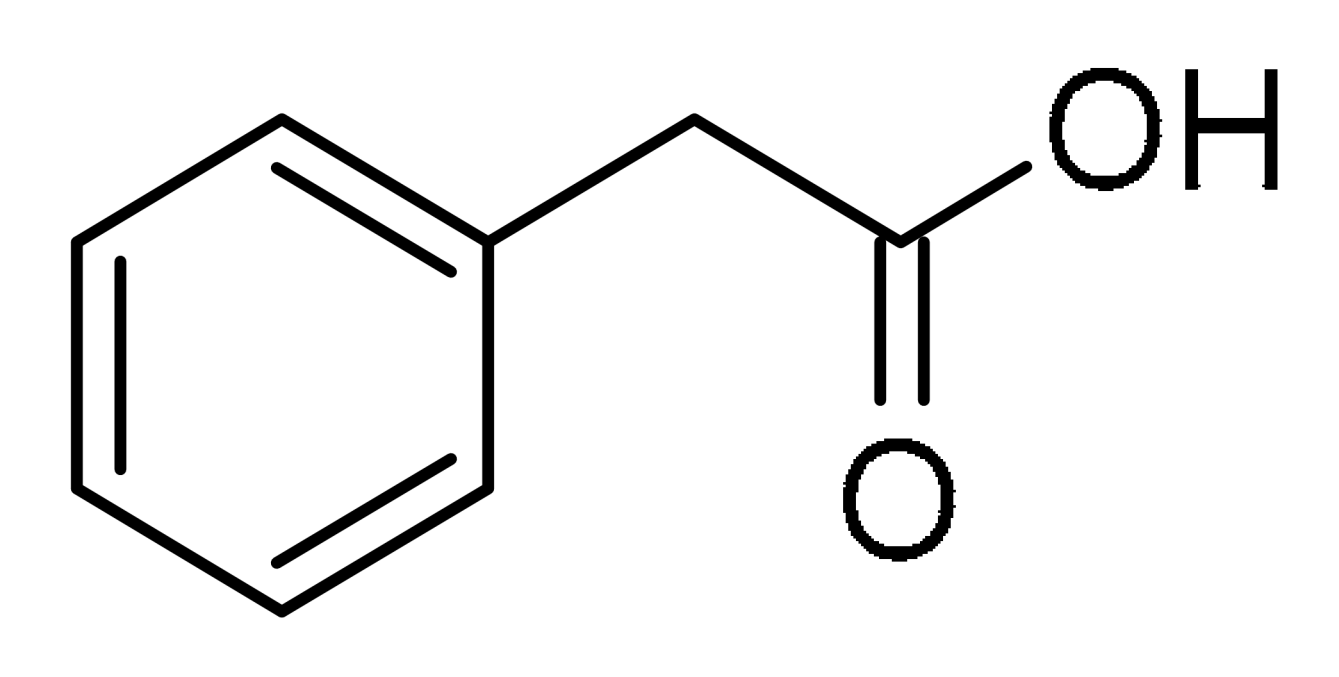	38	Citric acid	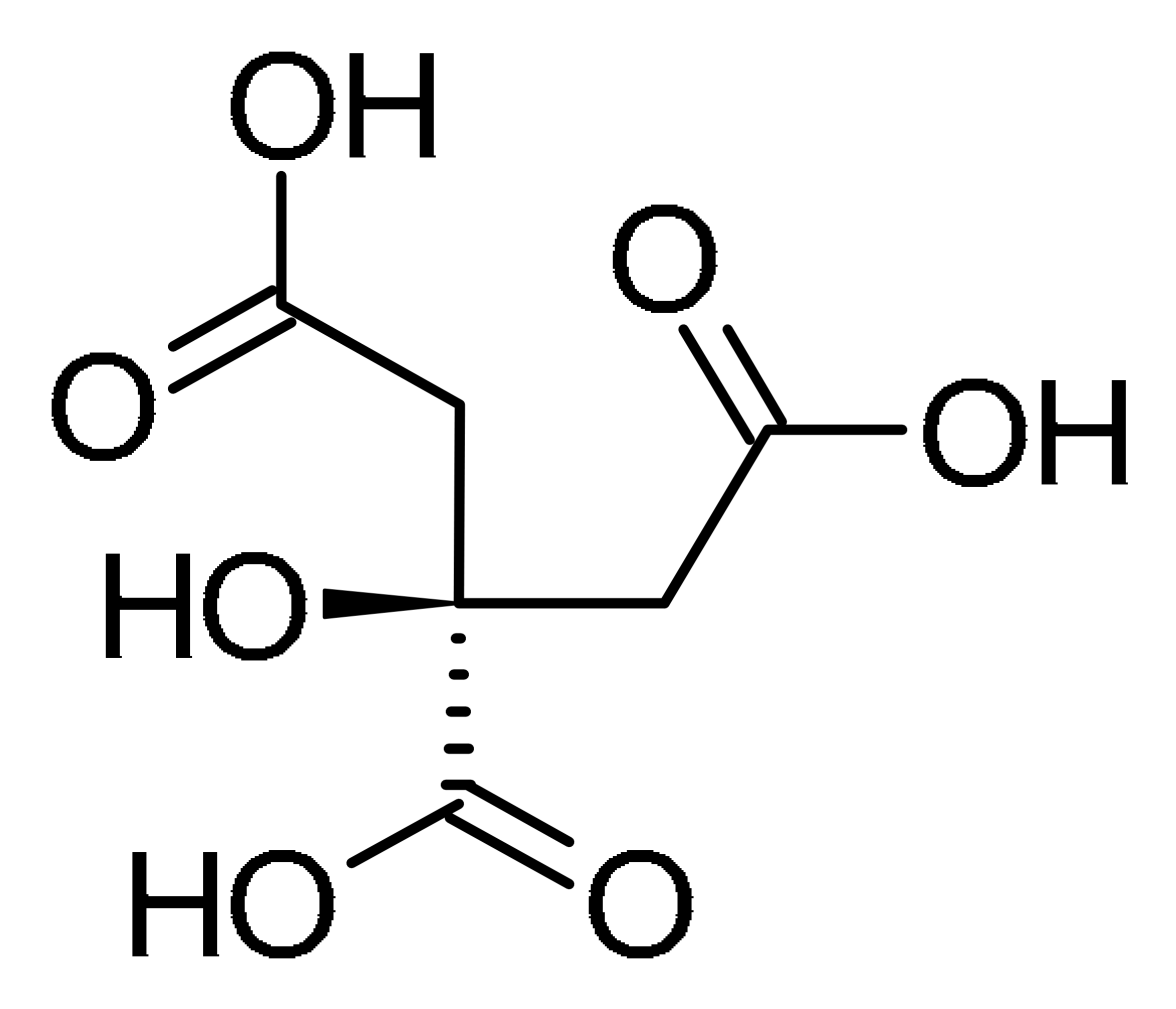
13	3-Nitrophenol	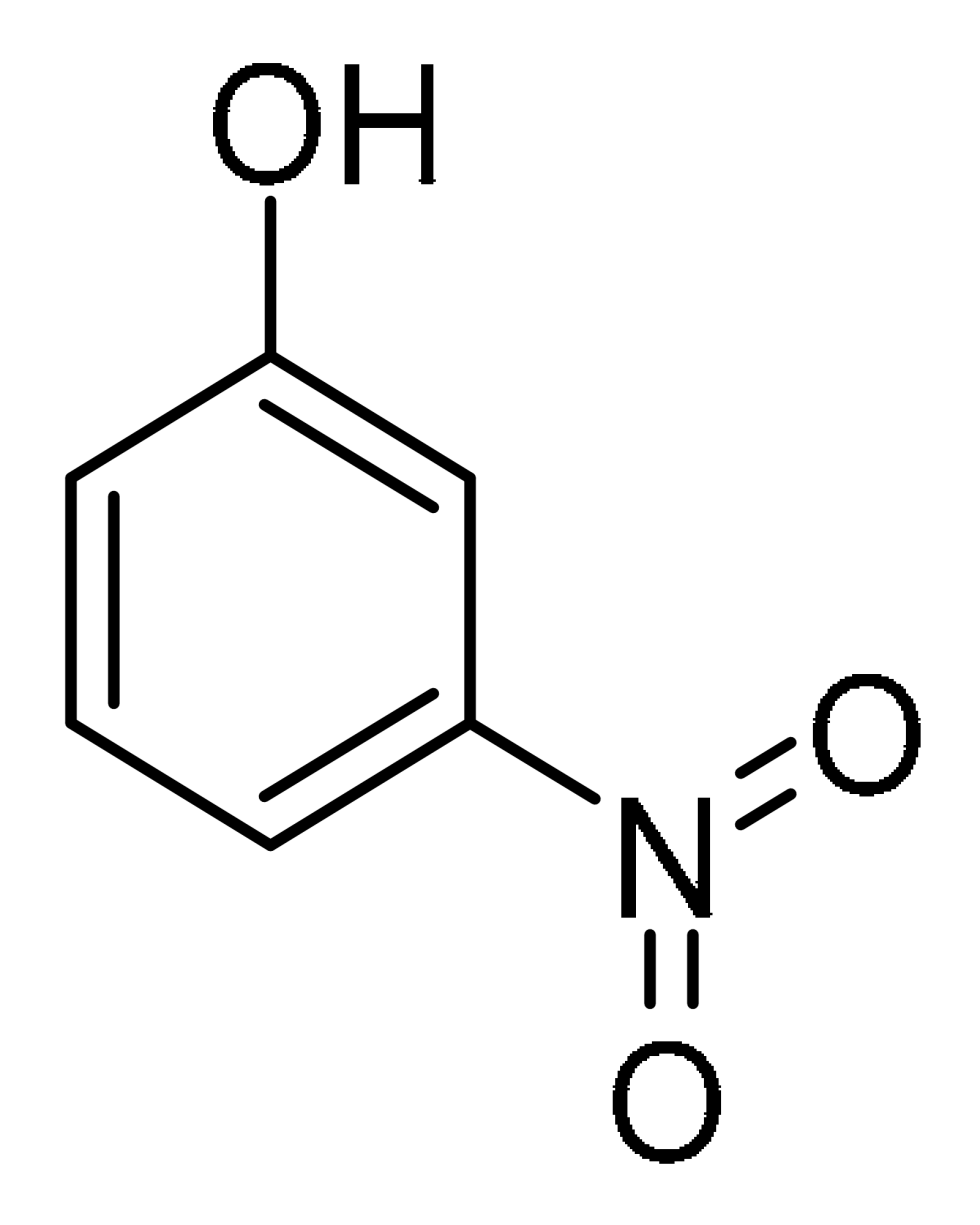	39	p-Toluenesulfonic acid	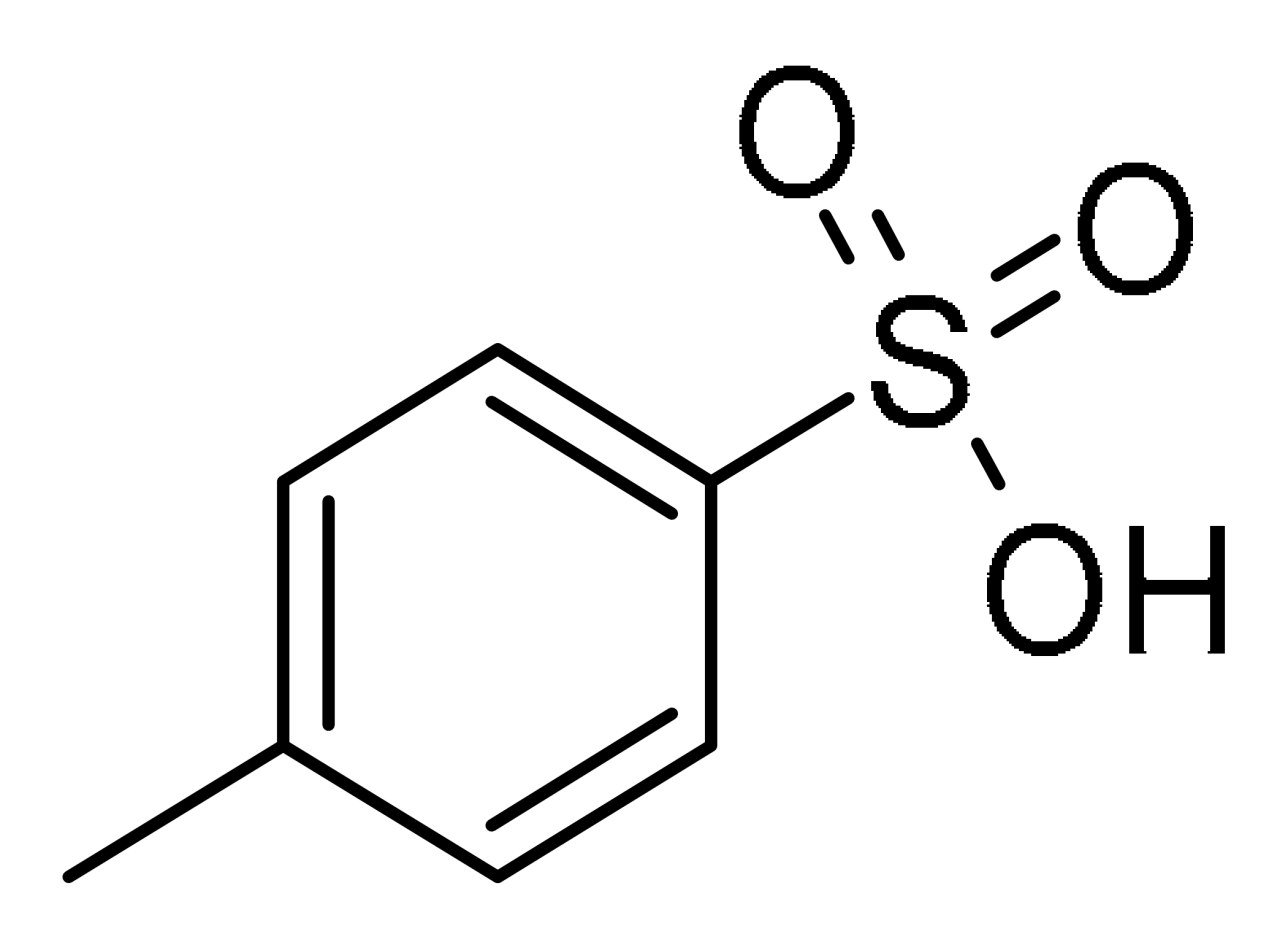
14	3-Chloro-4-hydroxyphenylacetic acid	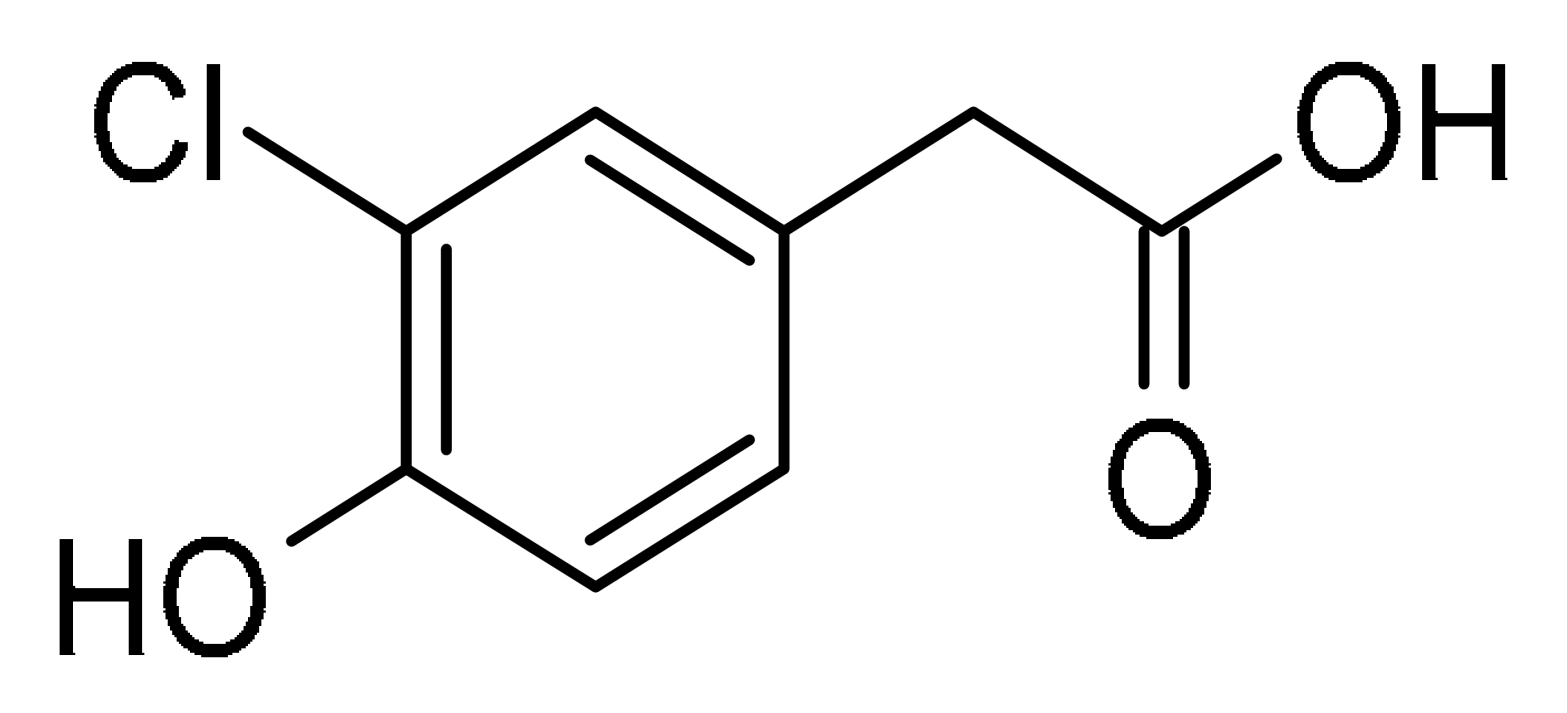	40	Methanesulfonic acid	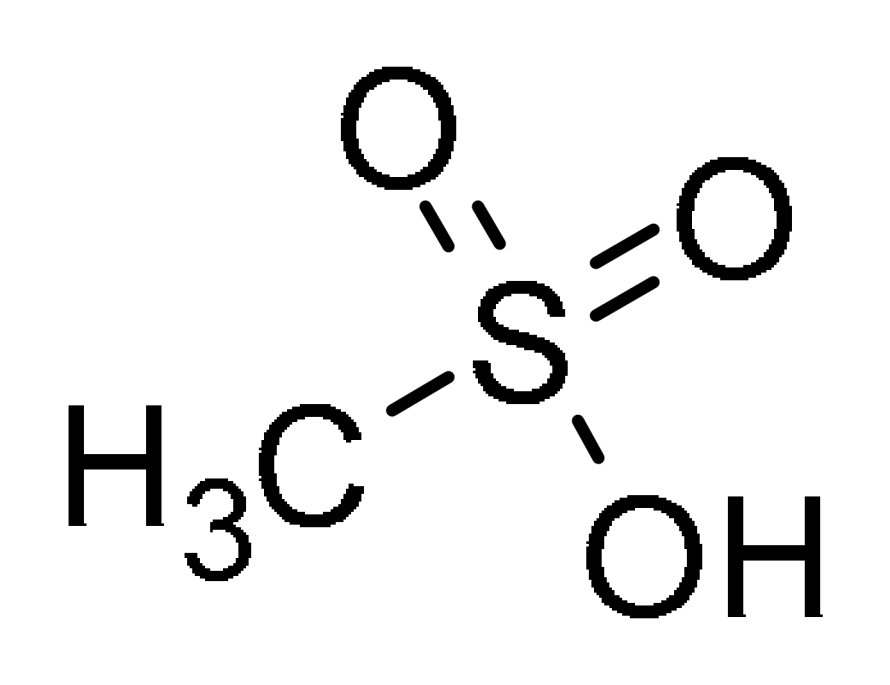
15	p-Coumaric acid	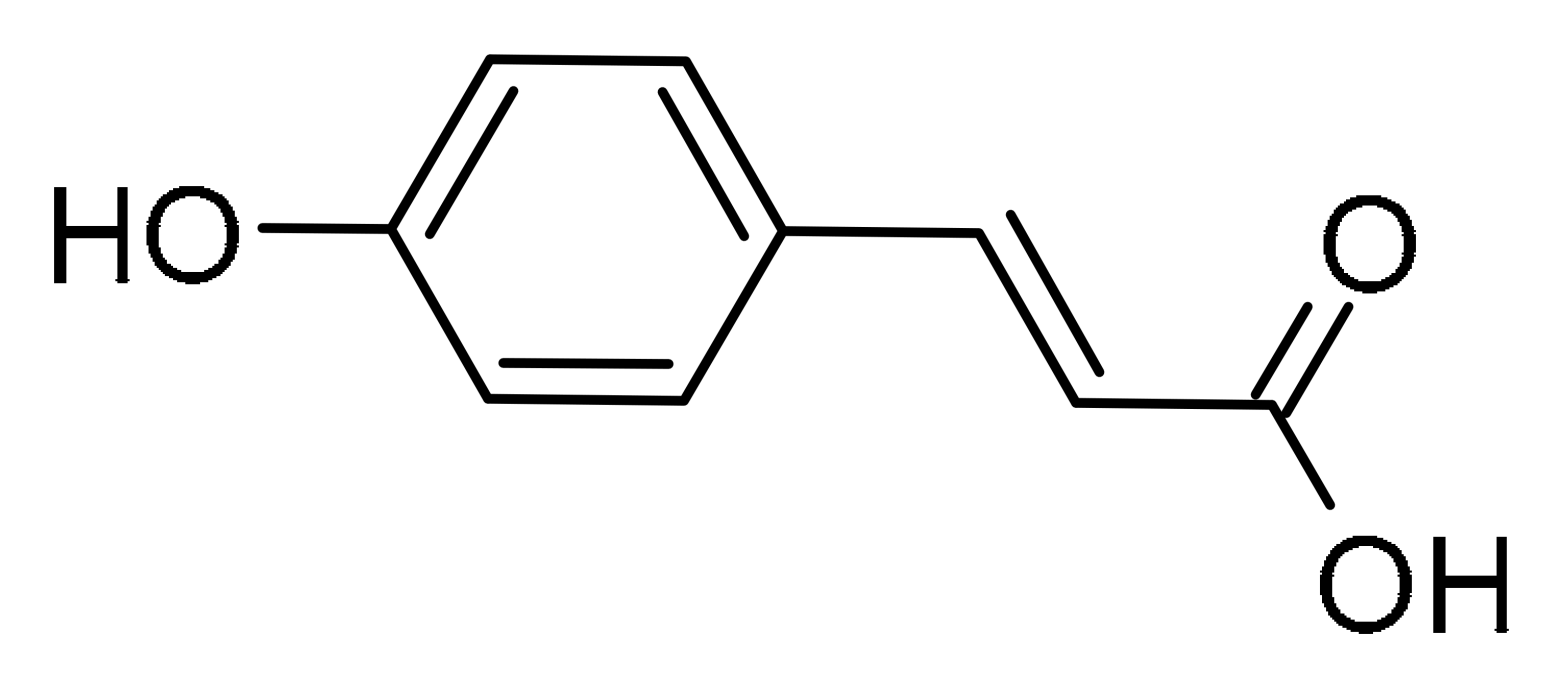	41	Trifluoroacetic acid	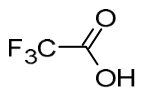
16	Phloretin	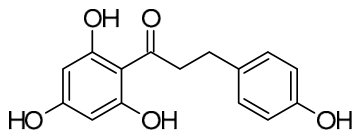	42	Naphthalene	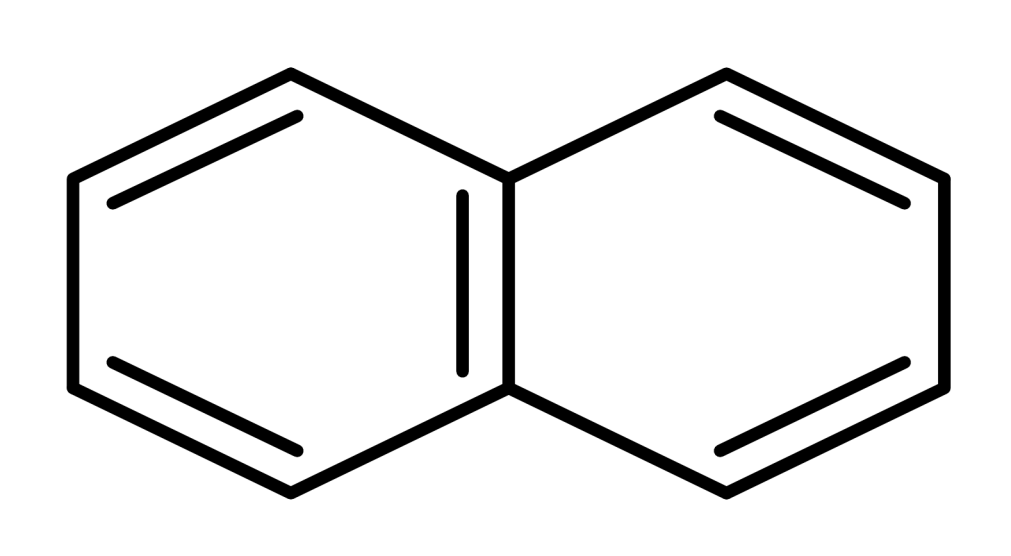
17	Benzoic acid	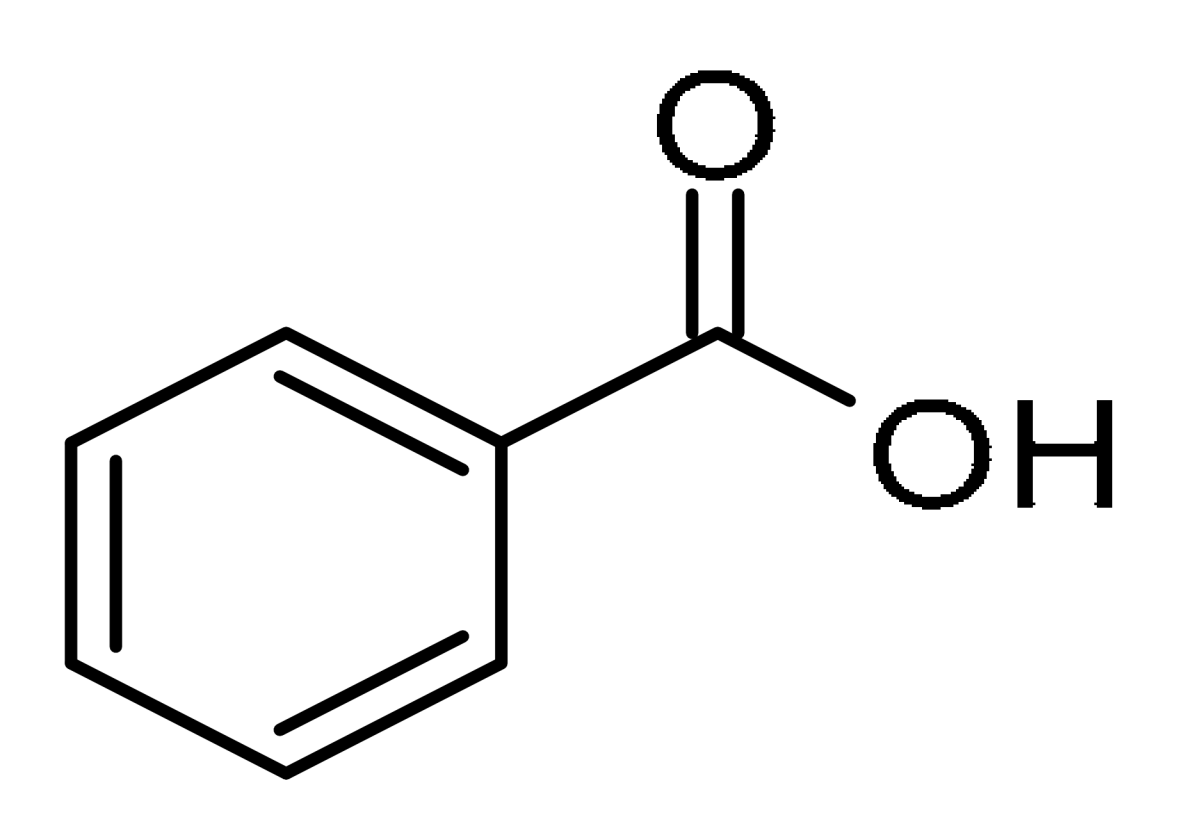	43	Biphenyl	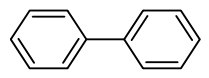
18	4-Fluorobenzoic acid	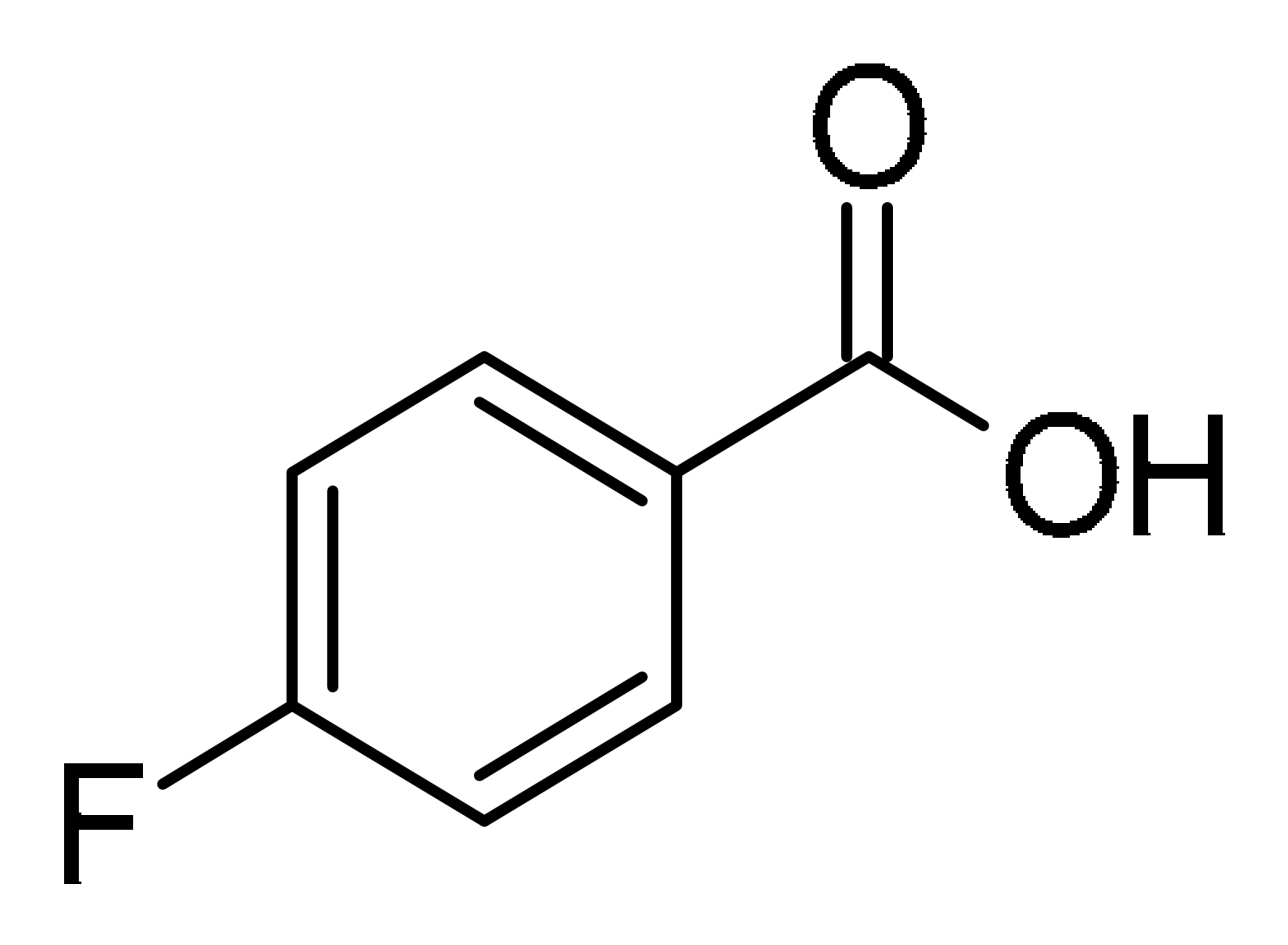	44	Fluorene	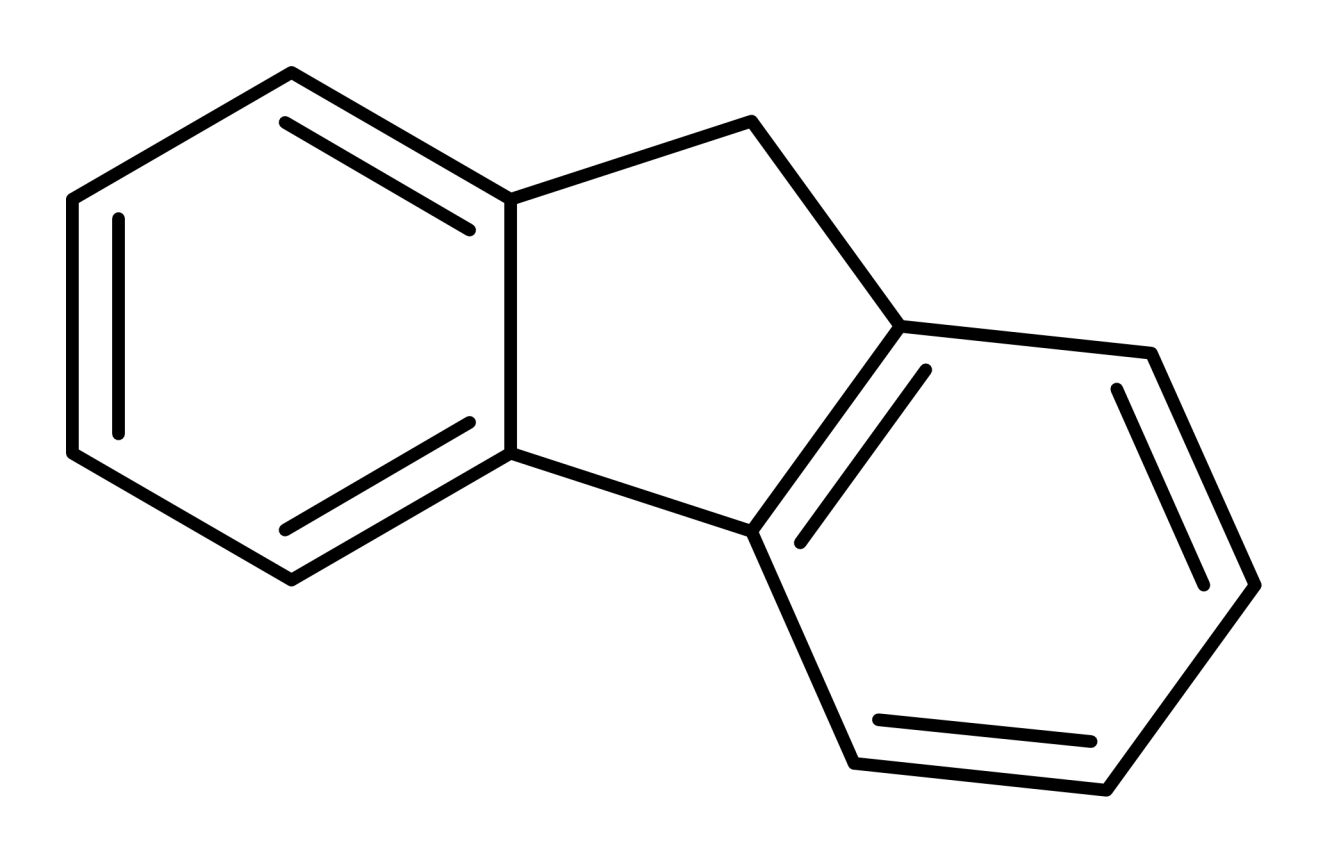
19	2-Fluorobenzoic acid	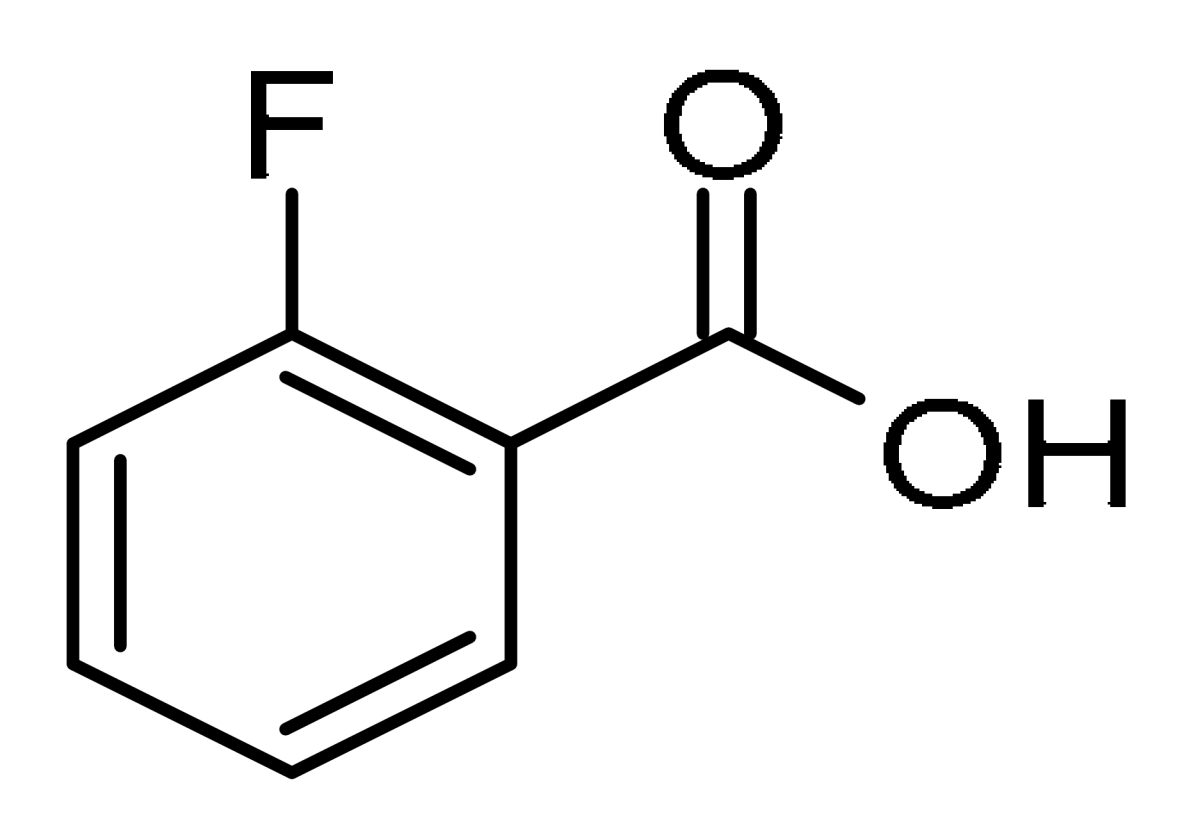	45	Hexamethylbenzene	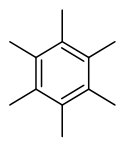
20	Phthalic acid	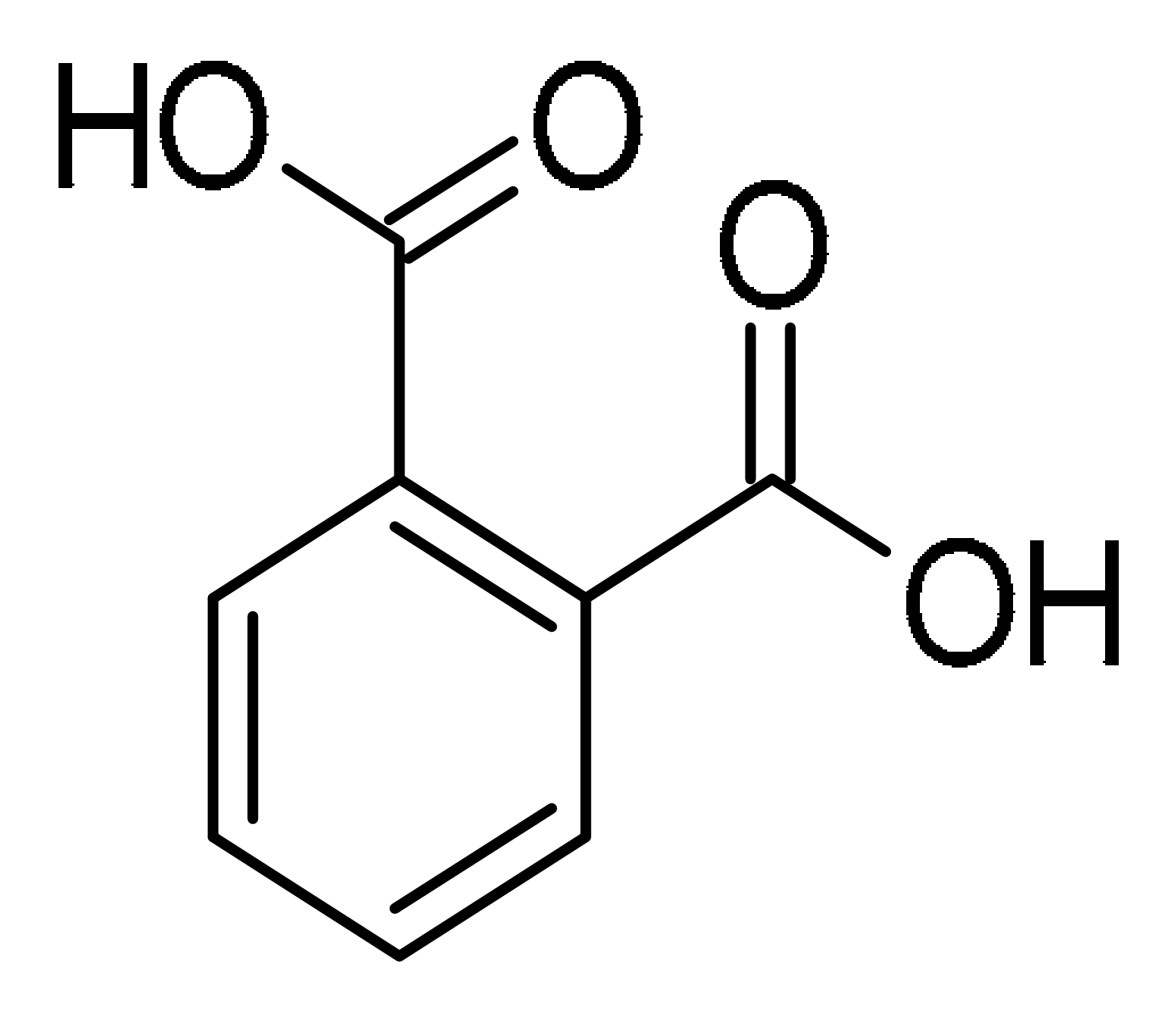	46	1,4-Dioxane	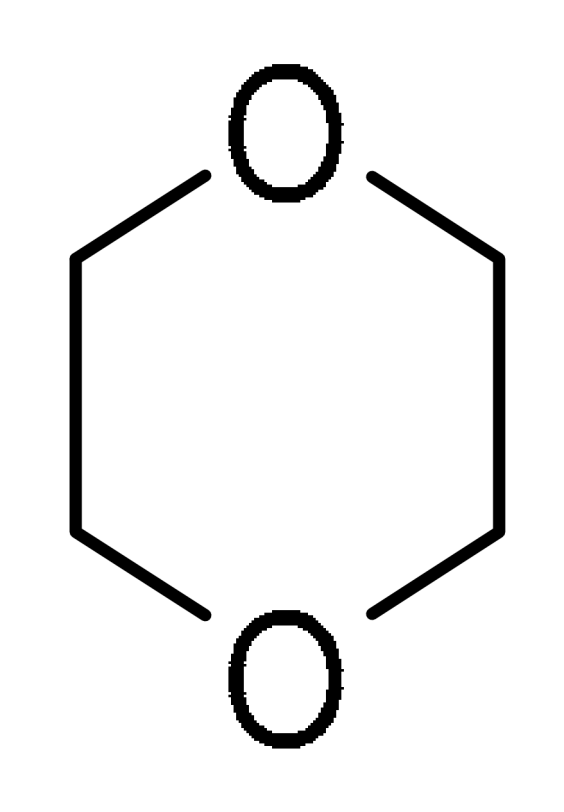
21	Isophthalic acid	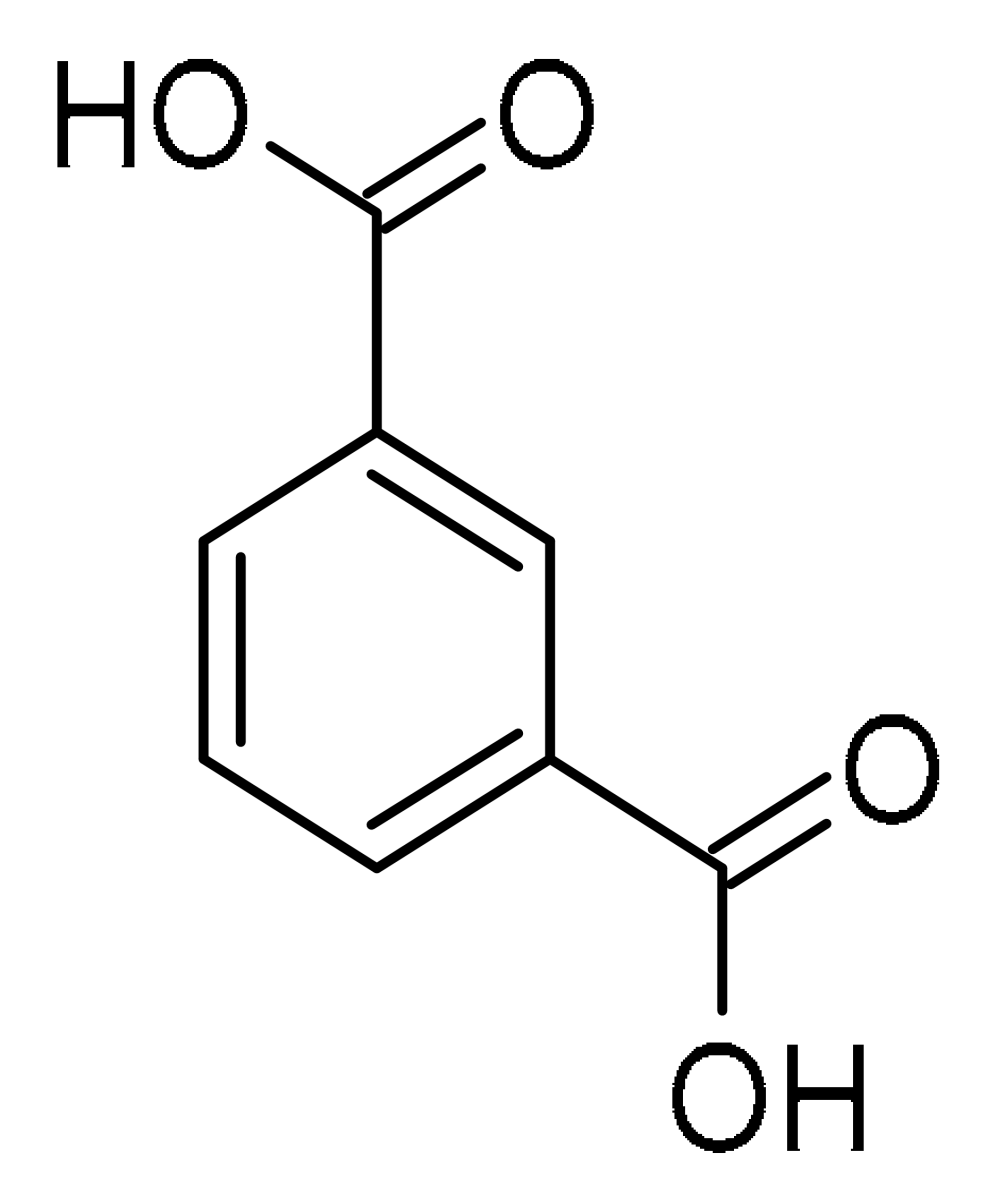	47	Camphor	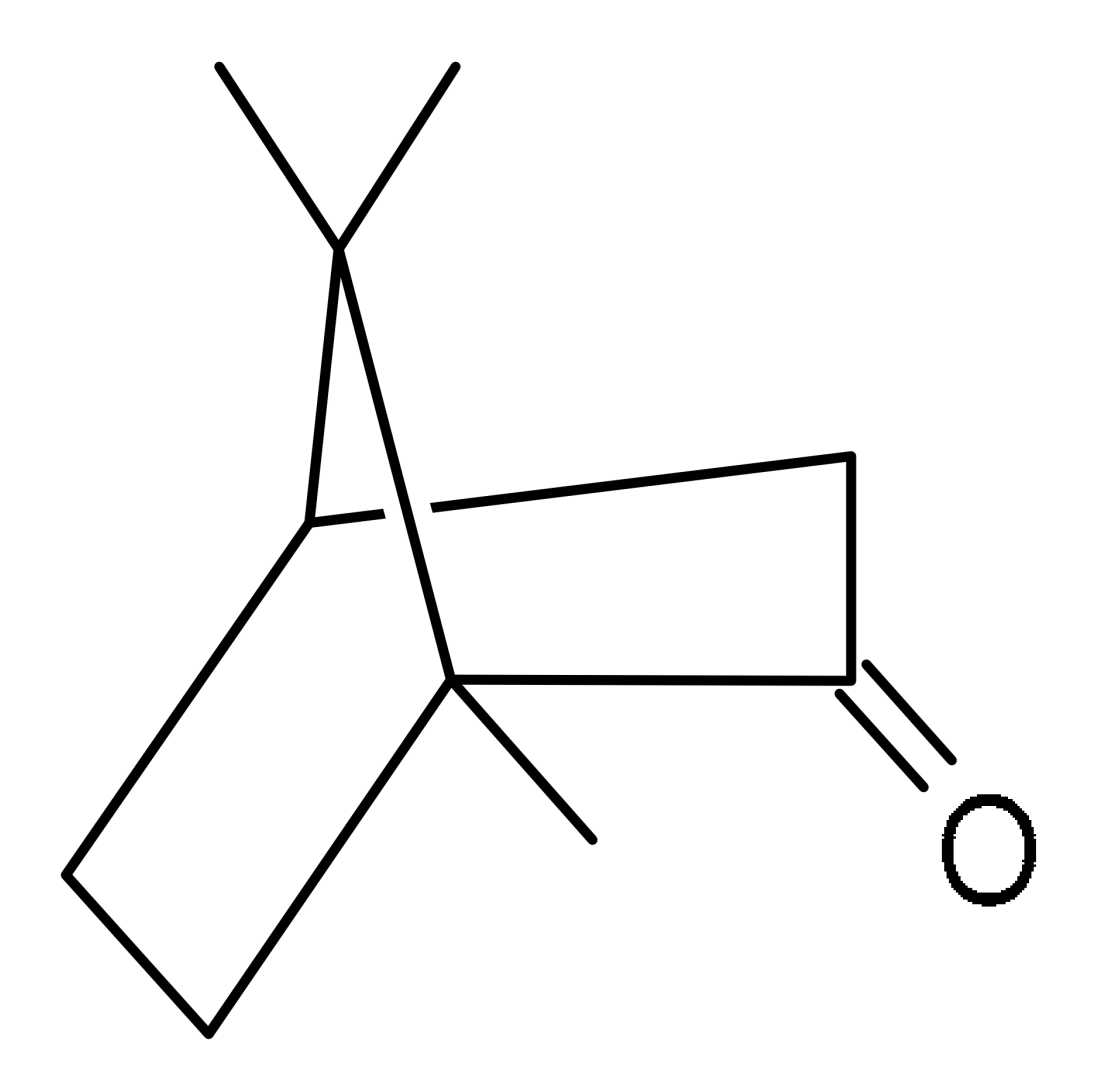
22	Terephthalic acid	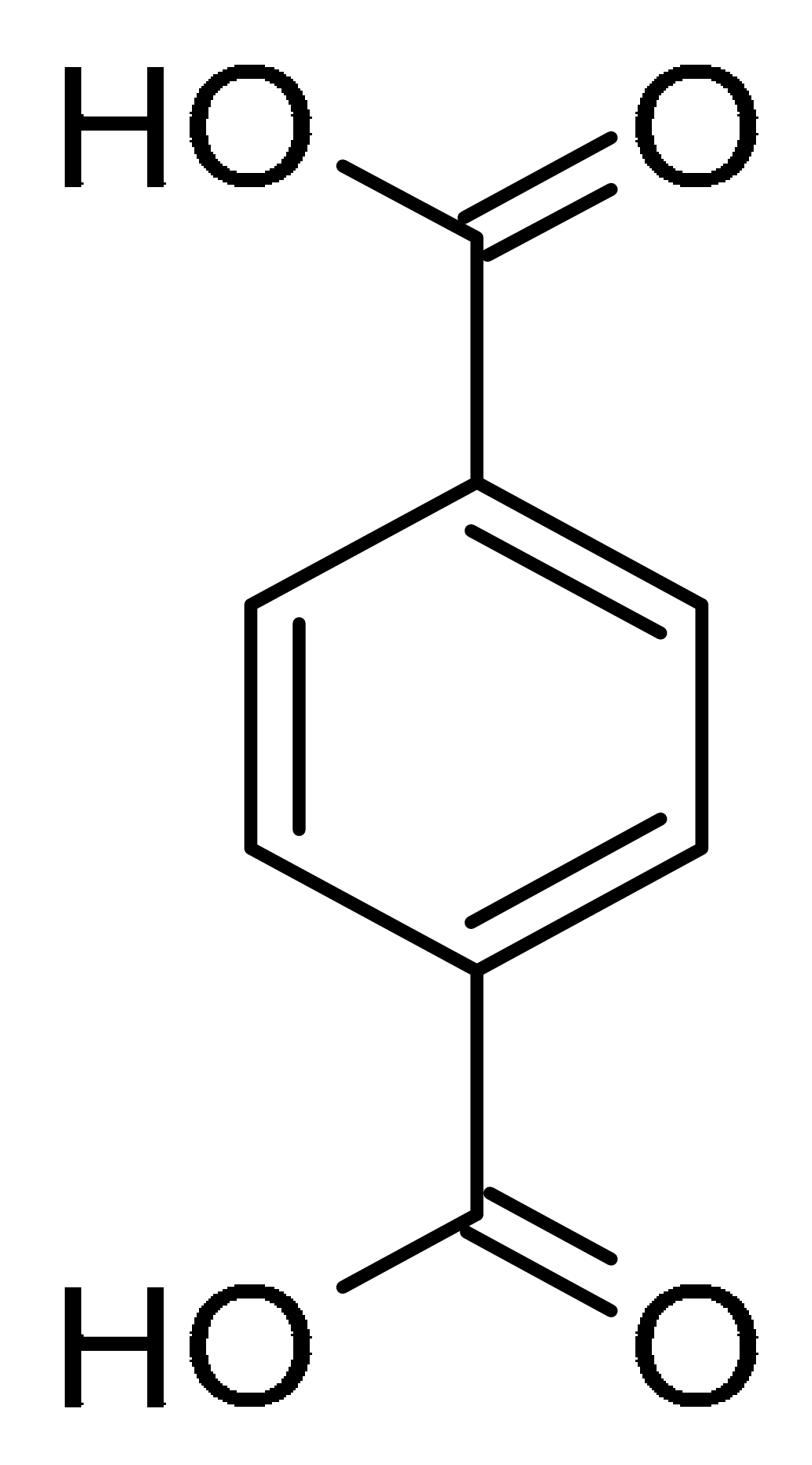	48	Riboflavin	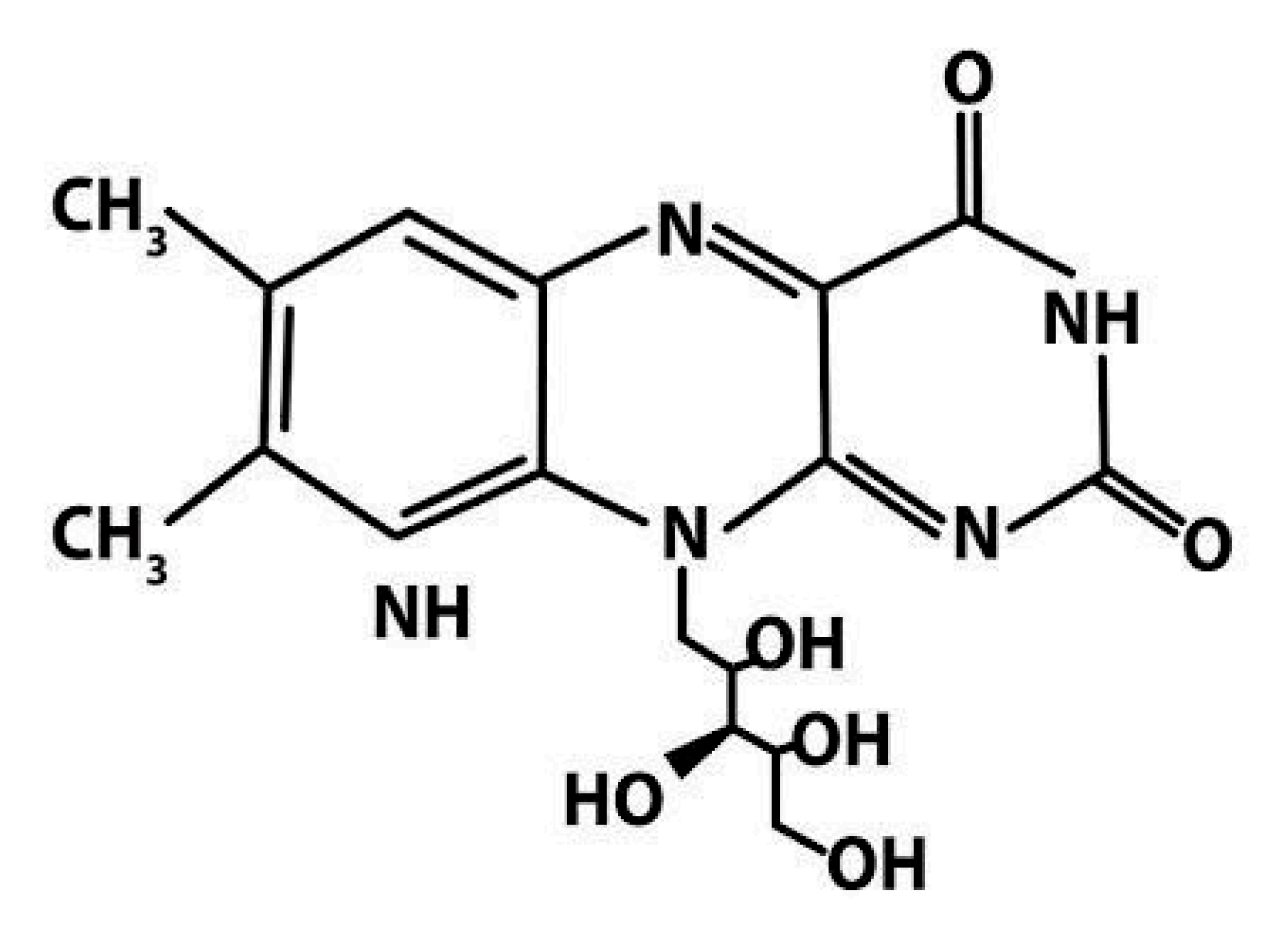
23	Trimesic acid	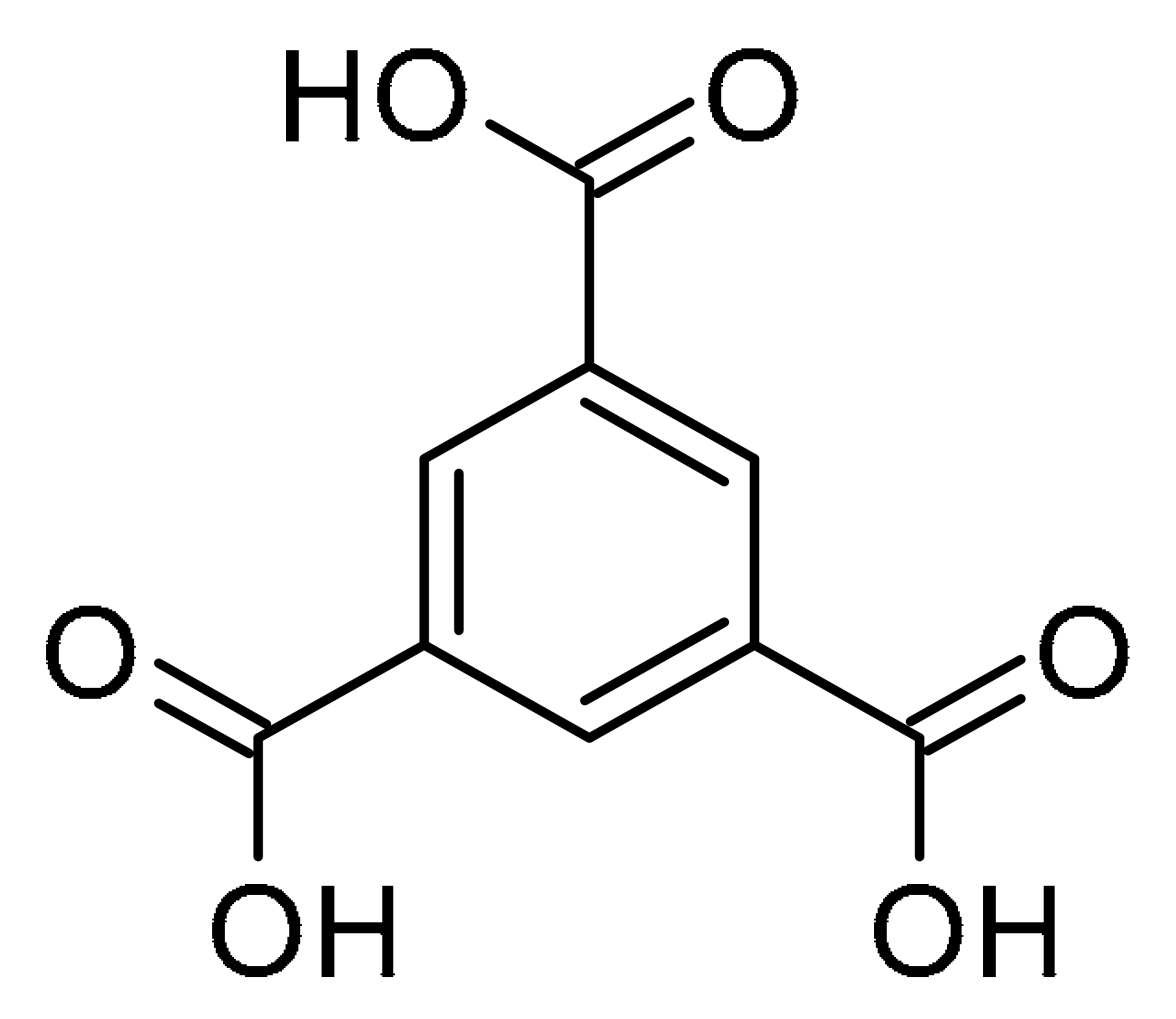	49	Picolinamide	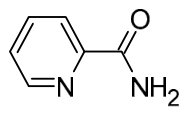
24	Gentisic acid	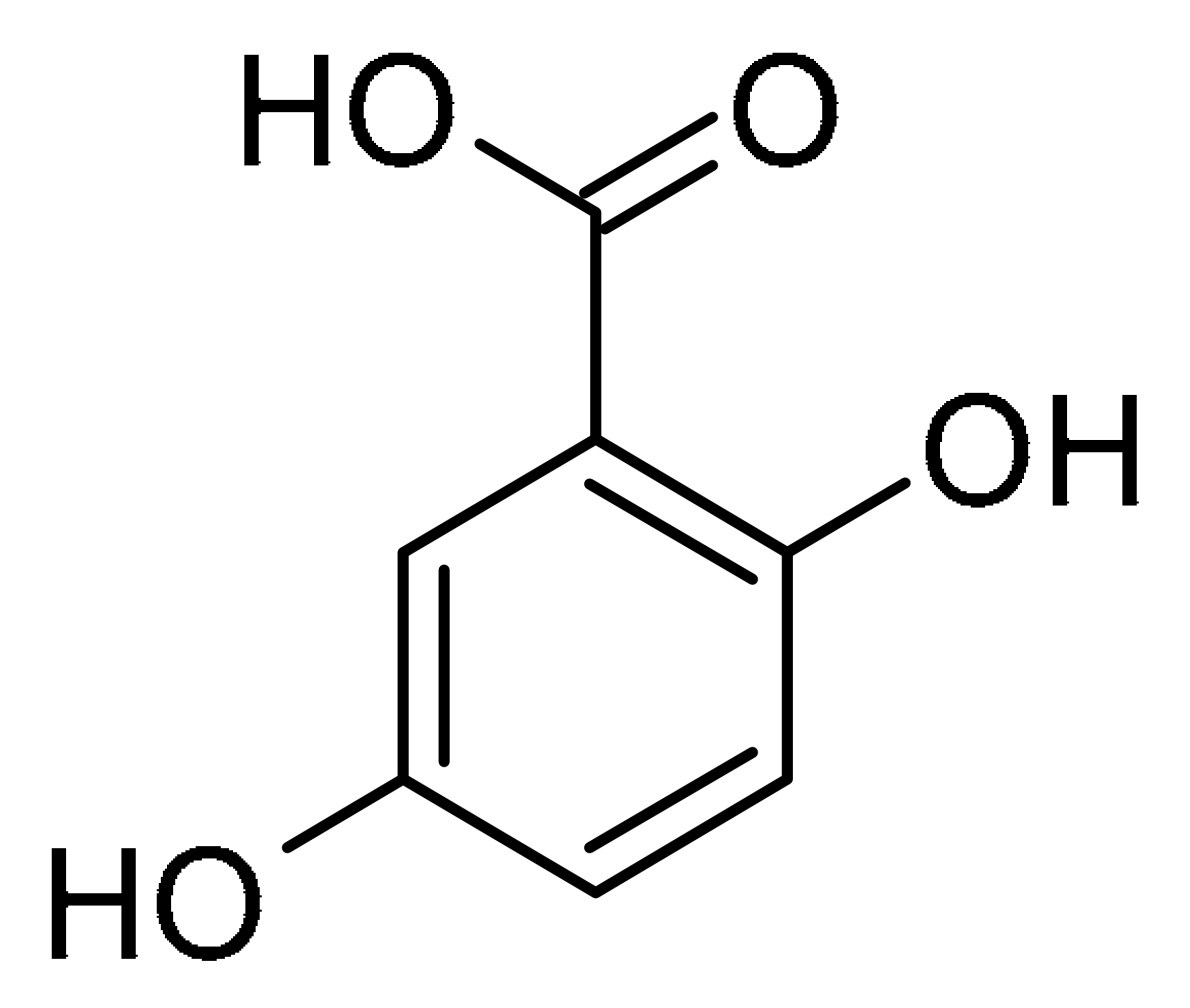	50	Nicotinamide	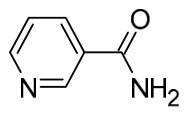
25	Gallic acid	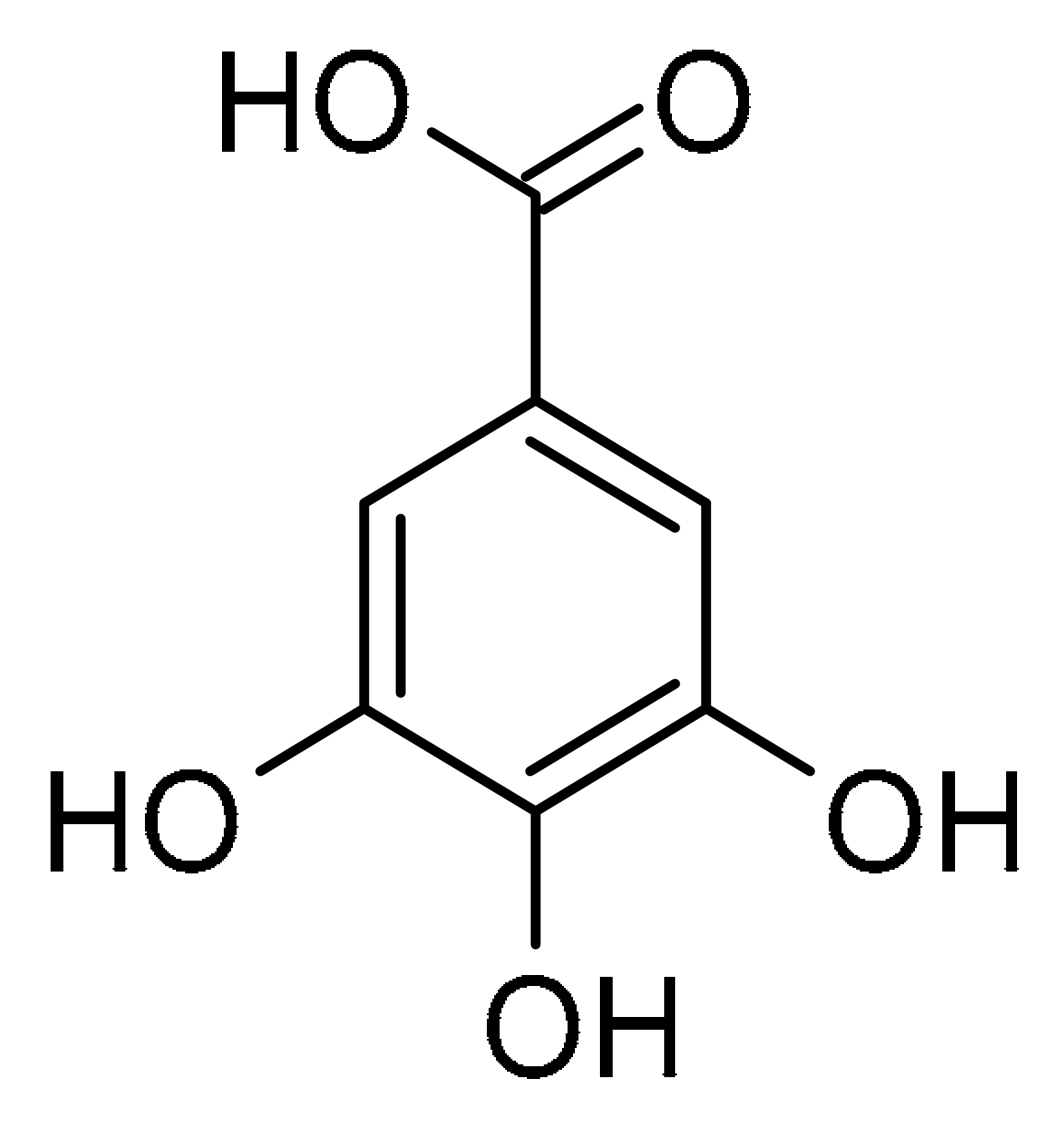	51	Isonicotinamide	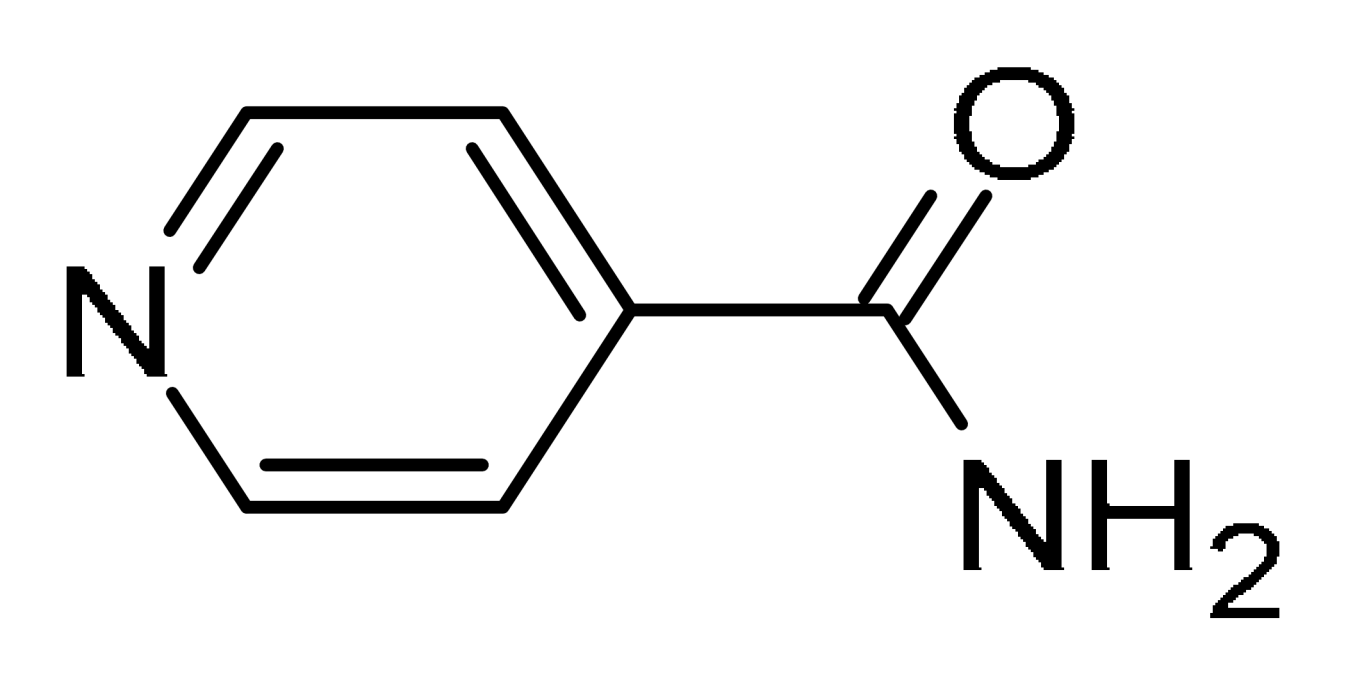
26	Caffeic acid	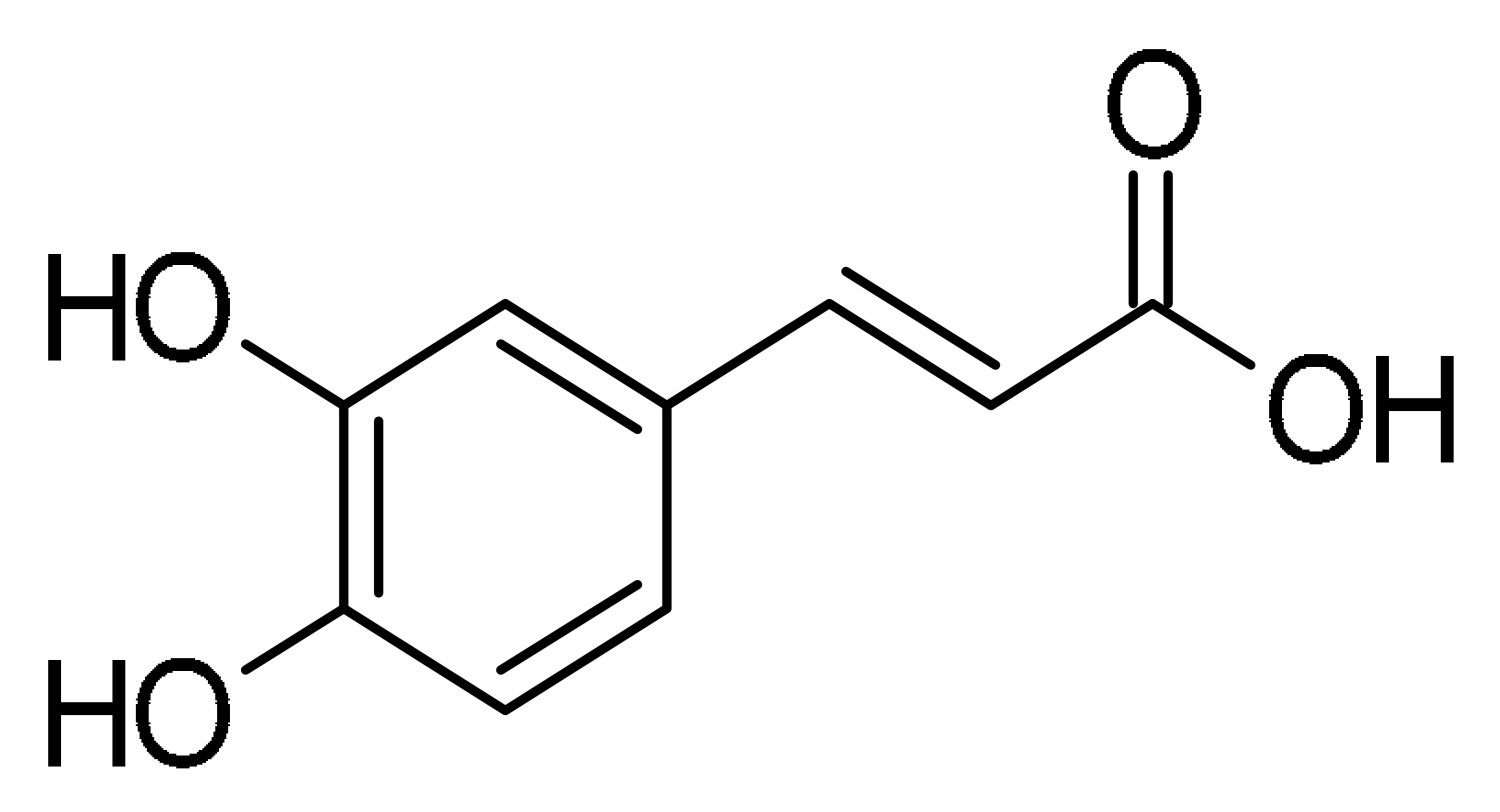			

**Table 2:** *DeepCocrystal* prediction scores (*p*), standard deviations (σ), and coformer classification.

		PIC			NIC			INIC		
N	Coformer	*p*	σ	Class	*p*	σ	Class	*p*	σ	Class
1	Salicylic acid	0.957	0.092	HPC	0.945	0.072	HPC	1.000	0.001	HPC
2	(S)-Mandelic acid	0.895	0.107	LPC1	0.690	0.290	LPC1	0.997	0.003	HPC
3	Malonic acid	0.952	0.074	HPC	0.926	0.135	LPC1	1.000	0.001	HPC
4	Succinic acid	0.817	0.191	LPC1	0.825	0.216	LPC1	0.999	0.002	HPC
5	Fumaric acid	0.981	0.033	HPC	0.979	0.037	HPC	1.000	0.000	HPC
6	Glutaric acid	0.907	0.186	LPC1	0.907	0.094	HPC	0.999	0.003	HPC
7	Adipic acid	0.867	0.169	LPC1	0.882	0.140	LPC1	0.999	0.003	HPC
8	Picolinic acid	0.436	0.414	LPC2	0.190	0.303	LPC2	0.848	0.280	LPC1
9	Naproxen	0.627	0.361	LPC1	0.393	0.415	LPC2	0.858	0.272	LPC1
10	Vanillin	0.989	0.011	HPC	0.892	0.238	LPC1	0.999	0.001	HPC
11	Vanillic acid	0.990	0.026	HPC	0.972	0.043	HPC	1.000	0.000	HPC
12	Phenylacetic acid	0.840	0.182	LPC1	0.638	0.363	LPC1	0.992	0.011	HPC
13	3-Nitrophenol	0.885	0.137	LPC1	0.777	0.207	LPC1	0.998	0.003	HPC
14	3-Chloro-4-hydroxyphenylacetic acid	0.997	0.006	HPC	0.993	0.011	HPC	0.999	0.002	HPC
15	p-Coumaric acid	0.986	0.011	HPC	0.812	0.219	LPC1	0.999	0.001	HPC
16	Phloretin	0.994	0.011	HPC	0.932	0.125	LPC1	0.999	0.002	HPC
17	Benzoic acid	0.715	0.321	LPC1	0.578	0.291	LPC1	0.987	0.014	HPC
18	4-Fluorobenzoic acid	0.901	0.250	LPC1	0.753	0.310	LPC1	0.996	0.008	HPC
19	2-Fluorobenzoic acid	0.866	0.251	LPC1	0.642	0.291	LPC1	0.994	0.017	HPC
20	Phthalic acid	0.947	0.085	HPC	0.855	0.172	LPC1	0.999	0.001	HPC
21	Isophthalic acid	0.941	0.106	LPC1	0.792	0.256	LPC1	0.998	0.002	HPC
22	Terephthalic acid	0.954	0.093	HPC	0.904	0.144	LPC1	0.999	0.001	HPC
23	Trimesic acid	0.999	0.001	HPC	0.993	0.008	HPC	1.000	0.000	HPC
24	Gentisic acid	0.998	0.005	HPC	0.996	0.007	HPC	1.000	0.000	HPC
25	Gallic acid	1.000	0.000	HPC	1.000	0.000	HPC	1.000	0.000	HPC
26	Caffeic acid	0.999	0.001	HPC	0.996	0.005	HPC	1.000	0.000	HPC
27	Ferulic acid	0.990	0.021	HPC	0.872	0.191	LPC1	0.999	0.002	HPC
28	Syringic acid	0.999	0.001	HPC	0.956	0.108	LPC1	0.999	0.003	HPC
29	Protocatechuic acid	0.994	0.016	HPC	0.996	0.007	HPC	1.000	0.000	HPC
30	2,6-Dihydroxybenzoic acid	0.997	0.004	HPC	0.997	0.003	HPC	1.000	0.000	HPC
31	4-Hydroxybenzoic acid	0.989	0.022	HPC	0.994	0.005	HPC	1.000	0.000	HPC
32	Nicotinic acid	0.022	0.046	NC	0.002	0.003	NC	0.327	0.363	LPC2
33	Isonicotinic acid	0.207	0.271	LPC2	0.027	0.046	NC	0.664	0.370	LPC1
34	Maleic acid	0.981	0.033	HPC	0.979	0.037	HPC	1.000	0.000	HPC
35	Malic acid	0.724	0.249	LPC1	0.634	0.352	LPC1	0.970	0.058	HPC
36	Tartaric acid	0.703	0.227	LPC1	0.612	0.337	LPC1	0.954	0.100	HPC
37	Citric acid	0.807	0.167	LPC1	0.630	0.325	LPC1	0.984	0.017	HPC
38	p-Toluenesulfonic acid	0.990	0.022	HPC	0.969	0.036	HPC	0.999	0.001	HPC
39	*Methanesulfonic acid*	0.850	0.221	LPC1	0.677	0.361	LPC1	0.973	0.065	HPC
40	Trifluoroacetic acid	0.993	0.016	HPC	0.980	0.015	HPC	0.998	0.005	HPC
41	Naphthalene	0.500	0.258	LPC1	0.349	0.242	LPC2	0.712	0.161	LPC1
42	Biphenyl	0.594	0.261	LPC1	0.441	0.246	LPC2	0.874	0.134	LPC1
43	Fluorene	0.366	0.343	LPC2	0.164	0.170	LPC2	0.674	0.163	LPC1
44	Hexamethylbenzene	0.800	0.301	LPC1	0.608	0.300	LPC1	0.999	0.003	HPC
45	1,4-Dioxane	0.833	0.239	LPC1	0.584	0.391	LPC1	0.998	0.007	HPC
46	Camphor	0.001	0.000	NC	0.005	0.010	NC	0.083	0.149	LPC2
47	Riboflavin	0.005	0.008	NC	0.001	0.002	NC	0.007	0.008	NC
48	Picolinamide				0.017	0.040	NC	0.142	0.272	LPC2
49	Nicotinamide	0.017	0.040	NC				0.046	0.125	LPC2
50	Isonicotinamide	0.142	0.272	LPC2	0.046	0.125	LPC2			

**Table 3:** Comparison of the predictions resulting solely from *DeepCocrystal* (DC) with those obtained after application of the pK_a_-based post-filtering procedure (DC-pK_a_).

		PIC		NIC		INIC	
N	Coformer	DC	DC-pK_a_	DC	DC-pK_a_	DC	DC-pK_a_
1	Salicylic acid	HPC	HPC	HPC	GZ	HPC	GZ
2	(S)-Mandelic acid	LPC1	LPC1	LPC1	LPC1	HPC	GZ
3	Malonic acid	HPC	HPC	LPC1	GZ	HPC	GZ
4	Succinic acid	LPC1	LPC1	LPC1	LPC1	HPC	HPC
5	Fumaric acid	HPC	HPC	HPC	GZ *	HPC	GZ
6	Glutaric acid	LPC1	LPC1	HPC	HPC	HPC	HPC
7	Adipic acid	LPC1	LPC1	LPC1	LPC1	HPC	HPC
8	Picolinic acid	LPC2	GZ	LPC2	GZ *	LPC1	GZ *
9	Naproxen	LPC1	LPC1	LPC2	LPC2	LPC1	LPC1
10	Vanillin	HPC	HPC	LPC1	LPC1	HPC	HPC
11	Vanillic acid	HPC	HPC	HPC	HPC	HPC	HPC
12	Phenylacetic acid	LPC1	LPC1	LPC1	LPC1	HPC	HPC
13	3-Nitrophenol	LPC1	LPC1	LPC1	LPC1	HPC	HPC
14	3-Chloro-4-hydroxyphenylacetic acid	HPC	HPC	HPC	HPC	HPC	HPC
15	p-Coumaric acid	HPC	HPC	LPC1	LPC1	HPC	HPC
16	Phloretin	HPC	HPC	LPC1	LPC1	HPC	HPC
17	Benzoic acid	LPC1	LPC1	LPC1	LPC1	HPC	HPC
18	4-Fluorobenzoic acid	LPC1	LPC1	LPC1	LPC1	HPC	HPC
19	2-Fluorobenzoic acid	LPC1	LPC1	LPC1	GZ	HPC	GZ *
20	Phthalic acid	HPC	HPC	LPC1	GZ	HPC	GZ
21	Isophthalic acid	LPC1	LPC1	LPC1	LPC1	HPC	GZ
22	Terephthalic acid	HPC	HPC	LPC1	LPC1	HPC	GZ
23	Trimesic acid	HPC	HPC	HPC	GZ	HPC	GZ
24	Gentisic acid	HPC	HPC	HPC	GZ	HPC	GZ
25	Gallic acid	HPC	HPC	HPC	HPC	HPC	HPC
26	Caffeic acid	HPC	HPC	HPC	HPC	HPC	HPC
27	Ferulic acid	HPC	HPC	LPC1	LPC1	HPC	HPC
28	Syringic acid	HPC	HPC	LPC1	LPC1	HPC	HPC
29	Protocatechuic acid	HPC	HPC	HPC	HPC	HPC	HPC
30	2,6-Dihydroxybenzoic acid	HPC	GZ	HPC	GZ	HPC	GZ
31	4-Hydroxybenzoic acid	HPC	HPC	HPC	HPC	HPC	HPC
32	Nicotinic acid	NC	NC	NC	NC	LPC2	GZ
33	Isonicotinic acid	LPC2	LPC2	NC	NC	LPC1	GZ
34	Maleic acid	HPC	HPC	HPC	GZ	HPC	GZ
35	Malic acid	LPC1	LPC1	LPC1	LPC1	HPC	GZ
36	Tartaric acid	LPC1	LPC1	LPC1	GZ	HPC	GZ
37	Citric acid	LPC1	LPC1	LPC1	GZ	HPC	GZ
38	p-Toluenesulfonic acid	HPC	GZ	HPC	SR	HPC	SR
39	Methanesulfonic acid	LPC1	SR	LPC1	SR	HPC	SR
40	Trifluoroacetic acid	HPC	GZ	HPC	SR	HPC	SR
41	Naphthalene	LPC1	LPC1	LPC2	LPC2	LPC1	LPC1
42	Biphenyl	LPC1	LPC1	LPC2	LPC2	LPC1	LPC1
43	Fluorene	LPC2	LPC2	LPC2	LPC2	LPC1	LPC1
44	Hexamethylbenzene	LPC1	LPC1	LPC1	LPC1	HPC	HPC
45	1,4-Dioxane	LPC1	LPC1	LPC1	LPC1	HPC	HPC
46	Camphor	NC	NC	NC	NC	LPC2	LPC2
47	Riboflavin	NC	NC	NC	NC	NC	NC
48	Picolinamide			NC	NC	LPC2	LPC2
49	Nicotinamide	NC	NC			LPC2	LPC2
50	Isonicotinamide	LPC2	LPC2	LPC2	LPC2		

* API–coformer cocrystals observed experimentally [16,17,18] and gray-zone cocrystal/salt. Labels reassigned by the pK_a_ rule are shown in red on a yellow ground.

**Table 4:** Per-API summary of the outcome of *DeepCocrystal* (DC) and of the pK_a_-based post-filtering (DC-pK_a_).

	Total Pairs	DC HPC	DC LPC1	DC LPC2	DC NC	DC-pK_a_ GZ	DC-pK_a_ SR	Unchanged	Reclassified	pK_a_ Not Applied	False Negatives
PIC	49	22	19	4	4	4	1	38	5	6	1
NIC	49	15	23	6	5	12	3	28	15	6	1
INIC	49	38	6	4	1	18	3	20	21	8	0
TOTAL	147	75	48	14	10	34	7	86	41	20	2

**Table 5:** Molecular descriptors used in the PCA.

N	Code	General Descriptors	N	Code	General (D13–D15) and Pyridinecarboxamide-Specific (D16–D24) Descriptors
1	D01_HBD	Hydrogen-bond donor count	13	D13_DAcompl	Donor–acceptor complementarity
2	D02_HBA	Hydrogen-bond acceptor count	14	D14_Flex	Molecular flexibility
3	D03_Hetero	Total heteroatom count	15	D15_SizeComp	Shape/size compatibility
4	D04_MW	Molecular weight	16	D16_RelPos	Relative pyridine-N/amide position
5	D05_TPSA	Topological polar surface area	17	D17_pyrNavail	Pyridine-N acceptor availability
6	D06_RotB	Rotatable-bond count	18	D18_amNHacc	Amide N–H donor accessibility
7	D07_ArRings	Aromatic-ring count	19	D19_amCOacc	Amide carbonyl acceptor accessibility
8	D08_fSP2	Fraction of sp2 atoms	20	D20_AcidPyr	Acid–pyridine heterosynthon likelihood
9	D09_logP	Calculated lipophilicity (logP)	21	D21_AcidAmide	Acid–amide interaction likelihood
10	D10_PolIdx	Molecular polarity index	22	D22_AmideAmide	Amide–amide homosynthon possibility
11	D11_FGclass	Functional-group class	23	D23_ArSurf	Aromatic-surface compatibility
12	D12_KeyGroups	Key cocrystal-group presence	24	D24_Compete	Homo/heteromolecular H-bond competition

**Table 6:** Eigenvalues, individual and cumulative explained variance of the global PCA (first ten components). Eigenvalues above 1 exceed the Kaiser threshold.

Component	Eigenvalue	Variance (%)	Cumulative (%)
PC1	9.274	38.64	38.64
PC2	4.510	18.79	57.43
PC3	3.471	14.47	71.90
PC4	2.425	10.10	82.00
PC5	1.638	6.82	88.82
PC6	0.998	4.16	92.98
PC7	0.519	2.16	95.15
PC8	0.308	1.28	96.43
PC9	0.276	1.15	97.58
PC10	0.186	0.77	98.36

**Table 7:** Horn’s parallel analysis: observed eigenvalues of the global PCA versus the mean and 95th-percentile eigenvalues obtained from 1000 independent column permutations of the autoscaled descriptor matrix. A component is retained where the observed eigenvalue exceeds the 95th-percentile null value.

Component	Observed Eigenvalue	Mean Null Eigenvalue	95th Pct Null Eigenvalue	Retain?
PC1	9.274	1.822	1.954	Yes
PC2	4.510	1.679	1.769	Yes
PC3	3.471	1.575	1.655	Yes
PC4	2.425	1.483	1.557	Yes
PC5	1.638	1.403	1.467	Yes
PC6	0.998	1.332	1.389	No
PC7	0.519	1.267	1.322	No
PC8	0.308	1.202	1.253	No

**Table 8:** Descriptor pairs with |Pearson r| >= 0.80 over the 147 API–coformer pairs, ranked by |r|. The 20 most strongly correlated of the 27 pairs above the threshold are listed; 12 pairs exceed |r| = 0.94 and 2 pairs are numerically collinear at |r| = 1.000 because the pyridine-N-availability and amide-donor/acceptor descriptors take only three distinct values (one per API scaffold) across the dataset.

Descriptor A	Descriptor B	r
D18_amNHacc	D19_amCOacc	1.000
D16_RelPos	D17_pyrNavail	1.000
D01_HBD	D13_DAcompl	0.988
D02_HBA	D05_TPSA	0.983
D07_ArRings	D23_ArSurf	0.981
D05_TPSA	D13_DAcompl	0.976
D13_DAcompl	D24_Compete	0.974
D01_HBD	D24_Compete	0.971
D02_HBA	D03_Hetero	0.958
D05_TPSA	D24_Compete	0.949
D02_HBA	D13_DAcompl	0.949
D03_Hetero	D05_TPSA	0.944
D20_AcidPyr	D21_AcidAmide	0.938
D01_HBD	D05_TPSA	0.935
D02_HBA	D24_Compete	0.907
D03_Hetero	D13_DAcompl	0.907
D04_MW	D15_SizeComp	−0.891
D01_HBD	D02_HBA	0.888
D03_Hetero	D24_Compete	0.885
D06_RotB	D14_Flex	0.870

**Table 9:** Variance inflation factor (VIF) of each descriptor, computed as the diagonal of the Moore–Penrose pseudo-inverse of the correlation matrix of the autoscaled 147 × 24 matrix; the four API-scaffold descriptors, which form two exactly collinear pairs, have an undefined VIF and are labeled accordingly. VIF > 10 is the conventional threshold for severe multicollinearity; VIF > 5 is a more conservative screening threshold. All 24 descriptors are reported.

Descriptor	VIF
D17_pyrNavail	collinear
D18_amNHacc	collinear
D19_amCOacc	collinear
D16_RelPos	collinear
D05_TPSA	398.0
D02_HBA	162.3
D24_Compete	132.5
D07_ArRings	115.6
D23_ArSurf	96.7
D06_RotB	89.9
D21_AcidAmide	69.5
D14_Flex	64.7
D13_DAcompl	52.8
D01_HBD	52.8
D10_PolIdx	51.2
D04_MW	48.6
D20_AcidPyr	42.9
D03_Hetero	28.9
D11_FGclass	27.8
D09_logP	16.2
D15_SizeComp	11.2
D08_fSP2	8.5
D12_KeyGroups	6.1
D22_AmideAmide	5.8

**Table 10:** Centroids (mean PC1–PC3 scores) and sizes of the predicted-outcome groups.

*DeepCocrystal* Prediction	n	PC1	PC2	PC3
Cocrystal-likely	123	+0.33	+0.14	−0.09
No-cocrystal	24	−1.69	−0.74	+0.45

**Table 11:** Centroids of the four *DeepCocrystal* classes in the global PC space.

*DeepCocrystal* Class	n	PC1	PC2	PC3
HPC	75	+0.92	+0.50	+0.06
LPC1	48	−0.59	−0.42	−0.32
LPC2	14	−3.22	−0.12	+0.42
NC	10	+0.45	−1.59	+0.50

**Table 12:** Centroids of the literature-evidence categories (A directly reported, B analogue-supported, C rule-probable, D rule-low-probability).

Evidence Category	n	PC1	PC2	PC3
A	35	−0.27	+0.13	−0.29
B	28	+0.21	+0.15	−0.01
C	54	+1.76	+0.02	+0.15
D	30	−3.06	−0.33	+0.07

**Table 13:** Centroids of the pK_a_-rule groups (CC, cocrystal-favored; GZ, salt–cocrystal continuum; SR, salt-favored).

pK_a_ Rule	n	PC1	PC2	PC3
CC	80	+0.94	−0.60	+0.27
GZ	36	+1.30	+1.28	−0.30
SR	7	−0.26	+2.08	−1.93

**Table 14:** Between-group share of the variance (percent) along PC1, PC2 and PC3 for each labeling. Higher values indicate cleaner separation along that axis.

Grouping	PC1 (%)	PC2 (%)	PC3 (%)
*DeepCocrystal prediction*	6.0	2.3	1.2
*DeepCocrystal class*	16.7	8.0	2.0
Evidence category	33.1	0.7	0.8
API scaffold	0.7	83.7	6.8
pK_a_ rule	2.7	19.7	8.3

**Table 15:** Quantitative cluster-separation metrics (silhouette score and Davies–Bouldin index) for each labeling, computed on the joint PC1–PC3 score space of the 147 API–coformer pairs.

Grouping	Groups	Pairs Used	Silhouette Score	Davies–Bouldin Index
*DeepCocrystal prediction*	2	147	0.145	3.378
*DeepCocrystal class*	4	147	0.012	4.254
Evidence category	4	147	0.005	7.137
API scaffold	3	147	0.128	3.569
pK_a_ rule	3	123	−0.020	2.678

**Table 16:** Centroids of the three API scaffolds in the global PC space.

API Scaffold	n	PC1	PC2	PC3
INIC	49	+0.27	+1.96	+0.49
NIC	49	+0.09	+0.69	+0.17
PIC	49	−0.36	−2.65	−0.66

**Table 17:** Correlation loadings of selected descriptors on the first three principal components (leading contributors). Sign indicates the direction of correlation; magnitude indicates the strength of association.

Descriptor	PC1	PC2	PC3
Topological polar surface area	+0.965	−0.155	+0.109
Donor–acceptor complementarity	+0.941	−0.173	+0.204
Hydrogen-bond acceptor count	+0.940	−0.177	+0.132
Total heteroatom count	+0.926	−0.142	+0.068
Hydrogen-bond donor count	+0.905	−0.164	+0.233
Molecular polarity index	+0.839	+0.051	−0.414
Calculated lipophilicity (logP)	−0.750	+0.013	+0.479
Pyridine-N acceptor availability	+0.084	+0.886	+0.253
Relative pyridine-N/amide position	+0.084	+0.886	+0.253
Amide N–H donor accessibility	+0.083	+0.882	+0.252
Amide carbonyl acceptor accessibility	+0.083	+0.882	+0.252
Acid–pyridine heterosynthon likelihood	+0.452	+0.736	−0.038
Acid–amide interaction likelihood	+0.495	+0.645	−0.096
Aromatic-surface compatibility	−0.418	−0.091	+0.778
Aromatic-ring count	−0.412	−0.119	+0.829
Key cocrystal-group presence	+0.439	+0.076	+0.079
Molecular weight	+0.401	−0.314	+0.767

**Table 18:** Explained variance of the three independent per-API PCA models, computed on the 20 coformer-driven descriptors that vary within a scaffold.

API	PC1 (%)	PC2 (%)	PC3 (%)	PC1+2 (%)	PC1+2+3 (%)
PIC	47.2	18.4	13.8	65.6	79.4
NIC	47.2	18.4	13.8	65.6	79.4
INIC	47.2	18.4	13.8	65.6	79.4

## Data Availability

All data supporting the reported results are contained in the article and in the Supplementary Materials. The complete coformer library, the DeepCocrystal prediction scores, the 24-descriptor matrix and the analysis script that regenerates every table and figure reported here are provided as Supplementary Materials; no new experimental data were created.

## Supplementary Materials

The following supporting information can be downloaded at: https://www.mdpi.com/article/10.3390/molecules31183354/s1, Tables S1 to S3, with evidence classification of coformers for PIC, NIC and INIC, respectively; Table S4, with pK_a_ and ΔpK_a_ values used for PIC, NIC and INIC post-filtering; Table S5, with coformer general-descriptor matrix; Table S6, with the numerical definition of the 24 descriptors used in the PCA; Table S7, with named exceptions to the cocrystal-likely/no-cocrystal group centroids in PC1–PC3 (2-SD criterion); Figures S1–S7, with the two- and three-dimensional PCA score plots and the biplot. The literature evidence compiled in Tables S1–S3 and the ionization constants listed in Table S4 are taken from references [7,12,13,14,15,16,17,18,19,20,21,22,23,24,25,26,27,28,29,30,31].
