# Supplementary material for: Interpretable Deep Supramolecular Language Modeling for Cocrystal Design: PCA-Augmented DeepCocrystal Screening of Picolinamide, Nicotinamide, and Isonicotinamide Systems
Source: Molecules. 2026 Sep 21;31(18):3354. doi: 10.3390/molecules31183354 (PMC13610686; doi:10.3390/molecules31183354)
Supplement: Supplementary file 1 [file molecules-31-03354-s001.zip › molecules-4454099-supplementary.pdf]

Supporting Information for

Interpretable Deep Supramolecular Language Modeling for Cocrystal Design: PCA-Augmented DeepCocrystal Screening of Picolinamide, Nicotinamide, and Isonicotinamide Systems

Bahadır Bozkurt[1,*], Ayberk Yilmaz[1,2], Gulce Ogruc Ildiz[1,3] and Rui Fausto[1,3,4]

1 Spectroscopy@IKU, IKU-SPECTRA Molecular Sciences and Spectroscopy Applied Research Center, Istanbul Kultur University, Atakoy Campus, Bakirkoy 34156, Istanbul, Türkiye

2 Faculty of Science, Department of Physics, Istanbul University, Vezneciler, Fatih, Istanbul 34134, Türkiye

3 Department of Physics, Faculty of Sciences and Letters, Istanbul Kultur University, Atakoy Campus, Bakirkoy 34156, Istanbul, Türkiye

4 CQC-IMS, Department of Chemistry, University of Coimbra, 3004-535 Coimbra, Portugal

* Corresponding author: b.bozkurt@iku.edu.tr

Index

Table S1. Evidence classification for PIC coformer candidates
Table S2. Evidence classification for NIC coformer candidates
Table S3. Evidence classification for INIC coformer candidates
Table S4. pK_a and ΔpK_a values used for PIC, NIC and INIC post-validation
Table S5. Coformer general-descriptor matrix
Table S6. Numerical definition of the 24 descriptors used in the PCA
Table S7. Named exceptions to the cocrystal-likely / no-cocrystal group centroids in PC1–PC3 (2-SD criterion)
Figure S1. PC1–PC2 score plot colored by DeepCocrystal binary outcome
Figure S2. PC1–PC2 score plot colored by the four DeepCocrystal certainty classes
Figure S3. Biplot overlaying the PC1–PC2 scores with the descriptors of largest combined loading
Figure S4. Three-dimensional PCA score plot colored by predicted cocrystal class
Figure S5. Three-dimensional PCA score plot colored by model API
Figure S6. Three-dimensional PCA score plot colored by evidence category
Figure S7. The three model APIs shown separately within the common PC1–PC2 space
References

Table S1. Evidence classification for PIC coformer candidates.[a]

No	API	Coformer	Evidence status	Comment	Ref.
1	PIC	Salicylic acid	A: Direct	Direct cocrystal reported.	[1]
2	PIC	(S)-Mandelic acid	A: Direct	Direct cocrystal reported.	[1]
3	PIC	Malonic acid	A: Direct	Direct cocrystal reported.	[1]
4	PIC	Succinic acid	A: Direct	Direct cocrystal reported.	[1]
5	PIC	Fumaric acid	A: Direct	Direct cocrystal reported.	[1]
6	PIC	Glutaric acid	A: Direct	Direct cocrystal reported.	[1]
7	PIC	Adipic acid	A: Direct	Direct cocrystal reported.	[1]
8	PIC	Picolinic acid	A: Direct	Direct cocrystal reported.	[2]
9	PIC	Naproxen	A: Direct	Direct cocrystal reported.	[3]
10	PIC	Vanillin	B: Analog	Analog cocrystal reported.	[4], [5]
11	PIC	Vanillic acid	B: Analog	Analog cocrystal reported.	[6], [7]
12	PIC	Phenylacetic acid	B: Analog	Analog cocrystal reported.	[8], [7]
13	PIC	3-Nitrophenol	B: Analog	Analog cocrystal reported.	[9], [5]
14	PIC	3-Chloro-4-hydroxyphenylacetic acid	B: Analog	Analog cocrystal reported.	[10], [7]
15	PIC	p-Coumaric acid	B: Analog	Analog cocrystal reported.	[11], [7]
16	PIC	Phloretin	B: Analog	Analog cocrystal reported.	[12], [5]
17	PIC	Benzoic acid	B: Analog	Analog cocrystal reported.	[13], [7]
18	PIC	4-Fluorobenzoic acid	B: Analog	Analog cocrystal reported.	[13], [7]
19	PIC	2-Fluorobenzoic acid	B: Analog	Analog cocrystal reported.	[13], [7]
20	PIC	Phthalic acid	C: Rule probable	Rule-based probable.	[7], [5]
21	PIC	Isophthalic acid	C: Rule probable	Rule-based probable.	[7], [5]
22	PIC	Terephthalic acid	C: Rule probable	Rule-based probable.	[7], [5]
23	PIC	Trimesic acid	C: Rule probable	Rule-based probable.	[7], [5]
24	PIC	Gentisic acid	C: Rule probable	Rule-based probable.	[7], [5]
25	PIC	Gallic acid	C: Rule probable	Rule-based probable.	[7], [5]
26	PIC	Caffeic acid	C: Rule probable	Rule-based probable.	[7], [5]
27	PIC	Ferulic acid	C: Rule probable	Rule-based probable.	[7], [5]
28	PIC	Syringic acid	C: Rule probable	Rule-based probable.	[7], [5]
29	PIC	Protocatechuic acid	C: Rule probable	Rule-based probable.	[7], [5]
30	PIC	2,6-Dihydroxybenzoic acid	C: Rule probable	Rule-based probable.	[7], [5]
31	PIC	4-Hydroxybenzoic acid	C: Rule probable	Rule-based probable.	[7], [5]
32	PIC	Nicotinic acid	C: Rule probable	Rule-based probable.	[7], [5]
33	PIC	Isonicotinic acid	C: Rule probable	Rule-based probable.	[7], [5]
34	PIC	Maleic acid	C: Rule probable	Rule-based probable.	[7], [5]
35	PIC	Malic acid	C: Rule probable	Rule-based probable.	[7], [5]
36	PIC	Tartaric acid	C: Rule probable	Rule-based probable.	[7], [5]
37	PIC	Citric acid	C: Rule probable	Rule-based probable.	[7], [5]
38	PIC	p-Toluenesulfonic acid	D: Low-probability	Rule-based low-probability.	[5], [14], [15], [16]
39	PIC	Methanesulfonic acid	D: Low-probability	Rule-based low-probability.	[5], [14], [15], [16]
40	PIC	Trifluoroacetic acid	D: Low-probability	Rule-based low-probability.	[5], [14], [15], [16]
41	PIC	Naphthalene	D: Low-probability	Rule-based low-probability.	[5], [14], [15], [16]
42	PIC	Biphenyl	D: Low-probability	Rule-based low-probability.	[5], [14], [15], [16]
43	PIC	Fluorene	D: Low-probability	Rule-based low-probability.	[5], [14], [15], [16]
44	PIC	Hexamethylbenzene	D: Low-probability	Rule-based low-probability.	[5], [14], [15], [16]
45	PIC	1,4-Dioxane	D: Low-probability	Rule-based low-probability.	[5], [14], [15], [16]
46	PIC	Camphor	D: Low-probability	Rule-based low-probability.	[5], [14], [15], [16]
47	PIC	Riboflavin	D: Low-probability	Rule-based low-probability.	[5], [14], [15], [16]
48	PIC	Picolinamide	N/A: Self-pair	Self-pair; not evaluated.	-
49	PIC	Nicotinamide	A: Direct	Direct cocrystal reported.	[2]
50	PIC	Isonicotinamide	A: Direct	Direct cocrystal reported.	[2]

[a] A, directly reported API-coformer cocrystal; B, analogue-supported pyridinecarboxamide cocrystal; C, rule-based probable candidate; D, rule-based low-probability candidate.

Table S2. Evidence classification for NIC coformer candidates.[a]

No	API	Coformer	Evidence status	Comment	Ref.
1	NIC	Salicylic acid	B: Analog	Analog cocrystal reported.	[1], [7]
2	NIC	(S)-Mandelic acid	A: Direct	Direct cocrystal reported.	[17]
3	NIC	Malonic acid	B: Analog	Analog cocrystal reported.	[1], [7]
4	NIC	Succinic acid	A: Direct	Direct cocrystal reported.	[18]
5	NIC	Fumaric acid	A: Direct	Direct cocrystal reported.	[19]
6	NIC	Glutaric acid	B: Analog	Analog cocrystal reported.	[1], [7]
7	NIC	Adipic acid	B: Analog	Analog cocrystal reported.	[1], [7]
8	NIC	Picolinic acid	A: Direct	Direct cocrystal reported.	[2]
9	NIC	Naproxen	B: Analog	Analog cocrystal reported.	[3], [7]
10	NIC	Vanillin	A: Direct	Direct cocrystal reported.	[4]
11	NIC	Vanillic acid	A: Direct	Direct cocrystal reported.	[6]
12	NIC	Phenylacetic acid	A: Direct	Direct cocrystal reported.	[8]
13	NIC	3-Nitrophenol	A: Direct	Direct cocrystal reported.	[9]
14	NIC	3-Chloro-4-hydroxyphenylacetic acid	A: Direct	Direct cocrystal reported.	[10]
15	NIC	p-Coumaric acid	A: Direct	Direct cocrystal reported.	[11]
16	NIC	Phloretin	A: Direct	Direct cocrystal reported.	[12]
17	NIC	Benzoic acid	B: Analog	Analog cocrystal reported.	[13], [7]
18	NIC	4-Fluorobenzoic acid	B: Analog	Analog cocrystal reported.	[13], [7]
19	NIC	2-Fluorobenzoic acid	B: Analog	Analog cocrystal reported.	[13], [7]
20	NIC	Phthalic acid	C: Rule probable	Rule-based probable.	[7], [5]
21	NIC	Isophthalic acid	C: Rule probable	Rule-based probable.	[7], [5]
22	NIC	Terephthalic acid	C: Rule probable	Rule-based probable.	[7], [5]
23	NIC	Trimesic acid	C: Rule probable	Rule-based probable.	[7], [5]
24	NIC	Gentisic acid	C: Rule probable	Rule-based probable.	[7], [5]
25	NIC	Gallic acid	C: Rule probable	Rule-based probable.	[7], [5]
26	NIC	Caffeic acid	C: Rule probable	Rule-based probable.	[7], [5]
27	NIC	Ferulic acid	C: Rule probable	Rule-based probable.	[7], [5]
28	NIC	Syringic acid	C: Rule probable	Rule-based probable.	[7], [5]
29	NIC	Protocatechuic acid	C: Rule probable	Rule-based probable.	[7], [5]
30	NIC	2,6-Dihydroxybenzoic acid	C: Rule probable	Rule-based probable.	[7], [5]
31	NIC	4-Hydroxybenzoic acid	C: Rule probable	Rule-based probable.	[7], [5]
32	NIC	Nicotinic acid	C: Rule probable	Rule-based probable.	[7], [5], [16]
33	NIC	Isonicotinic acid	C: Rule probable	Rule-based probable.	[7], [5], [16]
34	NIC	Maleic acid	C: Rule probable	Rule-based probable.	[7], [5]
35	NIC	Malic acid	C: Rule probable	Rule-based probable.	[7], [5]
36	NIC	Tartaric acid	C: Rule probable	Rule-based probable.	[7], [5]
37	NIC	Citric acid	C: Rule probable	Rule-based probable.	[7], [5]
38	NIC	p-Toluenesulfonic acid	D: Low-probability	Rule-based low-probability.	[5], [14], [15], [16]
39	NIC	Methanesulfonic acid	D: Low-probability	Rule-based low-probability.	[5], [14], [15], [16]
40	NIC	Trifluoroacetic acid	D: Low-probability	Rule-based low-probability.	[5], [14], [15], [16]
41	NIC	Naphthalene	D: Low-probability	Rule-based low-probability.	[5], [14], [15], [16]
42	NIC	Biphenyl	D: Low-probability	Rule-based low-probability.	[5], [14], [15], [16]
43	NIC	Fluorene	D: Low-probability	Rule-based low-probability.	[5], [14], [15], [16]
44	NIC	Hexamethylbenzene	D: Low-probability	Rule-based low-probability.	[5], [14], [15], [16]
45	NIC	1,4-Dioxane	D: Low-probability	Rule-based low-probability.	[5], [14], [15], [16]
46	NIC	Camphor	D: Low-probability	Rule-based low-probability.	[5], [14], [15], [16]
47	NIC	Riboflavin	D: Low-probability	Rule-based low-probability.	[5], [14], [15], [16]
48	NIC	Picolinamide	A: Direct	Direct cocrystal reported.	[2]
49	NIC	Nicotinamide	N/A: Self-pair	Self-pair; not evaluated.	-
50	NIC	Isonicotinamide	A: Direct	Direct cocrystal reported.	[2]

[a] A, directly reported API-coformer cocrystal; B, analogue-supported pyridinecarboxamide cocrystal; C, rule-based probable candidate; D, rule-based low-probability candidate.

Table S3. Evidence classification for INIC coformer candidates.[a]

No	API	Coformer	Evidence status	Comment	Ref.
1	INIC	Salicylic acid	B: Analog	Analog cocrystal reported.	[1], [7]
2	INIC	(S)-Mandelic acid	B: Analog	Analog cocrystal reported.	[1], [7]
3	INIC	Malonic acid	B: Analog	Analog cocrystal reported.	[1], [7]
4	INIC	Succinic acid	B: Analog	Analog cocrystal reported.	[1], [7]
5	INIC	Fumaric acid	B: Analog	Analog cocrystal reported.	[1], [7]
6	INIC	Glutaric acid	B: Analog	Analog cocrystal reported.	[1], [7]
7	INIC	Adipic acid	B: Analog	Analog cocrystal reported.	[1], [7]
8	INIC	Picolinic acid	A: Direct	Direct cocrystal reported.	[2]
9	INIC	Naproxen	B: Analog	Analog cocrystal reported.	[3], [5]
10	INIC	Vanillin	A: Direct	Direct cocrystal reported.	[4]
11	INIC	Vanillic acid	A: Direct	Direct cocrystal reported.	[6]
12	INIC	Phenylacetic acid	A: Direct	Direct cocrystal reported.	[8]
13	INIC	3-Nitrophenol	A: Direct	Direct cocrystal reported.	[9]
14	INIC	3-Chloro-4-hydroxyphenylacetic acid	A: Direct	Direct cocrystal reported.	[10]
15	INIC	p-Coumaric acid	B: Analog	Analog cocrystal reported.	[11], [7]
16	INIC	Phloretin	B: Analog	Analog cocrystal reported.	[12], [5]
17	INIC	Benzoic acid	A: Direct	Direct cocrystal reported.	[13]
18	INIC	4-Fluorobenzoic acid	A: Direct	Direct cocrystal reported.	[13]
19	INIC	2-Fluorobenzoic acid	A: Direct	Direct cocrystal reported.	[13]
20	INIC	Phthalic acid	C: Rule probable	Rule-based probable.	[7], [5]
21	INIC	Isophthalic acid	C: Rule probable	Rule-based probable.	[7], [5]
22	INIC	Terephthalic acid	C: Rule probable	Rule-based probable.	[7], [5]
23	INIC	Trimesic acid	C: Rule probable	Rule-based probable.	[7], [5]
24	INIC	Gentisic acid	C: Rule probable	Rule-based probable.	[7], [5]
25	INIC	Gallic acid	C: Rule probable	Rule-based probable.	[7], [5]
26	INIC	Caffeic acid	C: Rule probable	Rule-based probable.	[7], [5]
27	INIC	Ferulic acid	C: Rule probable	Rule-based probable.	[7], [5]
28	INIC	Syringic acid	C: Rule probable	Rule-based probable.	[7], [5]
29	INIC	Protocatechuic acid	C: Rule probable	Rule-based probable.	[7], [5]
30	INIC	2,6-Dihydroxybenzoic acid	C: Rule probable	Rule-based probable.	[7], [5]
31	INIC	4-Hydroxybenzoic acid	C: Rule probable	Rule-based probable.	[7], [5]
32	INIC	Nicotinic acid	C: Rule probable	Rule-based probable.	[7], [5], [16]
33	INIC	Isonicotinic acid	C: Rule probable	Rule-based probable.	[7], [5], [16]
34	INIC	Maleic acid	C: Rule probable	Rule-based probable.	[7], [5]
35	INIC	Malic acid	C: Rule probable	Rule-based probable.	[7], [5]
36	INIC	Tartaric acid	C: Rule probable	Rule-based probable.	[7], [5]
37	INIC	Citric acid	C: Rule probable	Rule-based probable.	[7], [5]
38	INIC	p-Toluenesulfonic acid	D: Low-probability	Rule-based low-probability.	[5], [14], [15], [16]
39	INIC	Methanesulfonic acid	D: Low-probability	Rule-based low-probability.	[5], [14], [15], [16]
40	INIC	Trifluoroacetic acid	D: Low-probability	Rule-based low-probability.	[5], [14], [15], [16]
41	INIC	Naphthalene	D: Low-probability	Rule-based low-probability.	[5], [14], [15], [16]
42	INIC	Biphenyl	D: Low-probability	Rule-based low-probability.	[5], [14], [15], [16]
43	INIC	Fluorene	D: Low-probability	Rule-based low-probability.	[5], [14], [15], [16]
44	INIC	Hexamethylbenzene	D: Low-probability	Rule-based low-probability.	[5], [14], [15], [16]
45	INIC	1,4-Dioxane	D: Low-probability	Rule-based low-probability.	[5], [14], [15], [16]
46	INIC	Camphor	D: Low-probability	Rule-based low-probability.	[5], [14], [15], [16]
47	INIC	Riboflavin	D: Low-probability	Rule-based low-probability.	[5], [14], [15], [16]
48	INIC	Picolinamide	A: Direct	Direct cocrystal reported.	[2]
49	INIC	Nicotinamide	A: Direct	Direct cocrystal reported.	[2]
50	INIC	Isonicotinamide	N/A: Self-pair	Self-pair; not evaluated.	-

[a] A, directly reported API-coformer cocrystal; B, analogue-supported pyridinecarboxamide cocrystal; C, rule-based probable candidate; D, rule-based low-probability candidate.

Table S4. pK_a and ΔpK_a values used for PIC, NIC and INIC post-validation.[a]

No	Coformer	pK_a	Type	PIC ΔpK_a	PIC rule	NIC ΔpK_a	NIC rule	INIC ΔpK_a	INIC rule
1	Salicylic acid	2.97	acid	−1.47	CC	0.38	GZ	0.64	GZ
2	(S)-Mandelic acid	3.41	acid	−1.91	CC	−0.06	CC	0.20	GZ
3	Malonic acid	2.83	acid	−1.33	CC	0.52	GZ	0.78	GZ
4	Succinic acid	4.21	acid	−2.71	CC	−0.86	CC	−0.60	CC
5	Fumaric acid	3.03	acid	−1.53	CC	0.32	GZ	0.58	GZ
6	Glutaric acid	4.34	acid	−2.84	CC	−0.99	CC	−0.73	CC
7	Adipic acid	4.43	acid	−2.93	CC	−1.08	CC	−0.82	CC
8	Picolinic acid	1.01	acid	0.49	GZ	2.34	GZ	2.60	GZ
9	Naproxen	4.15	acid	−2.65	CC	−0.80	CC	−0.54	CC
10	Vanillin	7.40	acid	−5.90	CC	−4.05	CC	−3.79	CC
11	Vanillic acid	4.50	acid	−3.00	CC	−1.15	CC	−0.89	CC
12	Phenylacetic acid	4.31	acid	−2.81	CC	−0.96	CC	−0.70	CC
13	3-Nitrophenol	8.36	acid	−6.86	CC	−5.01	CC	−4.75	CC
14	3-Chloro-4-hydroxyphenylacetic acid	4.30	acid	−2.80	CC	−0.95	CC	−0.69	CC
15	p-Coumaric acid	4.64	acid	−3.14	CC	−1.29	CC	−1.03	CC
16	Phloretin	7.05	acid	−5.55	CC	−3.70	CC	−3.44	CC
17	Benzoic acid	4.20	acid	−2.70	CC	−0.85	CC	−0.59	CC
18	4-Fluorobenzoic acid	4.14	acid	−2.64	CC	−0.79	CC	−0.53	CC
19	2-Fluorobenzoic acid	3.27	acid	−1.77	CC	0.08	GZ	0.34	GZ
20	Phthalic acid	2.95	acid	−1.45	CC	0.40	GZ	0.66	GZ
21	Isophthalic acid	3.54	acid	−2.04	CC	−0.19	CC	0.07	GZ
22	Terephthalic acid	3.51	acid	−2.01	CC	−0.16	CC	0.10	GZ
23	Trimesic acid	3.12	acid	−1.62	CC	0.23	GZ	0.49	GZ
24	Gentisic acid	2.43	acid	−0.93	CC	0.92	GZ	1.18	GZ
25	Gallic acid	4.40	acid	−2.90	CC	−1.05	CC	−0.79	CC
26	Caffeic acid	4.62	acid	−3.12	CC	−1.27	CC	−1.01	CC
27	Ferulic acid	4.58	acid	−3.08	CC	−1.23	CC	−0.97	CC
28	Syringic acid	4.50	acid	−3.00	CC	−1.15	CC	−0.89	CC
29	Protocatechuic acid	4.48	acid	−2.98	CC	−1.13	CC	−0.87	CC
30	2,6-Dihydroxybenzoic acid	1.23	acid	0.27	GZ	2.12	GZ	2.38	GZ
31	4-Hydroxybenzoic acid	4.54	acid	−3.04	CC	−1.19	CC	−0.93	CC
32	Nicotinic acid	2.07	acid	−0.57	CC	1.28	GZ	1.54	GZ
33	Isonicotinic acid	1.84	acid	−0.34	CC	1.51	GZ	1.77	GZ
34	Maleic acid	1.92	acid	−0.42	CC	1.43	GZ	1.69	GZ
35	Malic acid	3.40	acid	−1.90	CC	−0.05	CC	0.21	GZ
36	Tartaric acid	2.98	acid	−1.48	CC	0.37	GZ	0.63	GZ
37	Citric acid	3.13	acid	−1.63	CC	0.22	GZ	0.48	GZ
38	p-Toluenesulfonic acid	−0.70	acid	2.20	GZ	4.05	SR	4.31	SR
39	Methanesulfonic acid	−1.90	acid	3.40	SR	5.25	SR	5.51	SR
40	Trifluoroacetic acid	0.23	acid	1.27	GZ	3.12	SR	3.38	SR
41	Naphthalene	n.a.	neutral	n.a.	n.a.	n.a.	n.a.	n.a.	n.a.
42	Biphenyl	n.a.	neutral	n.a.	n.a.	n.a.	n.a.	n.a.	n.a.
43	Fluorene	n.a.	neutral	n.a.	n.a.	n.a.	n.a.	n.a.	n.a.
44	Hexamethylbenzene	n.a.	neutral	n.a.	n.a.	n.a.	n.a.	n.a.	n.a.
45	1,4-Dioxane	n.a.	neutral	n.a.	n.a.	n.a.	n.a.	n.a.	n.a.
46	Camphor	n.a.	neutral	n.a.	n.a.	n.a.	n.a.	n.a.	n.a.
47	Riboflavin	6.70	acid	−5.20	CC	−3.35	CC	−3.09	CC
48	Picolinamide	n.a.	neutral	n.a.	n.a.	n.a.	n.a.	n.a.	n.a.
49	Nicotinamide	n.a.	neutral	n.a.	n.a.	n.a.	n.a.	n.a.	n.a.
50	Isonicotinamide	n.a.	neutral	n.a.	n.a.	n.a.	n.a.	n.a.	n.a.

[a] PIC, picolinamide; NIC, nicotinamide; INIC, isonicotinamide. pK_a (API-H^+) values used were PIC = 1.50, NIC = 3.35 and INIC = 3.61 [20]. Coformer pK_a values are from [20], except that of 2,6-dihydroxybenzoic acid (1.23), which is taken from the redetermination of Lowe and Smith [21]. $\Delta pK_a = pK_a(\text{API-H}^+) - pK_a(\text{coformer})$. CC, cocrystal-compatible ($\Delta pK_a < 0$); GZ, salt/cocrystal gray-zone ($0 \leq \Delta pK_a \leq 3$); SR, salt-risk ($\Delta pK_a > 3$); n.a., not applicable. The pK_a rule is based on acid-base cocrystal/salt literature [14,16].

Table S5. Coformer general-descriptor matrix.[a]

Complete general molecular descriptor matrix for the 50 coformers screened against picolinamide, nicotinamide and isonicotinamide, computed directly from structure (SMILES) with RDKit. Abbreviations: MW, molecular weight (g mol^{-1}); HBD, hydrogen-bond donors; HBA, hydrogen-bond acceptors (Lipinski N+O count); Het, total heteroatom count (N+O+S+halogens); TPSA, topological polar surface area ($Å^2$); RotB, number of rotatable bonds; ArR, number of aromatic rings; fSP2, fraction of heavy atoms with sp^2 hybridization or aromatic character; logP, calculated Crippen lipophilicity; FG class, functional-group class assigned by the substructure-matching scheme described in the manuscript; PolIdx, molecular polarity index; DAcompl, donor–acceptor complementarity; Flex, molecular flexibility; SizeComp, shape/size compatibility with the API scaffold.

No	Coformer	MW	HBD	HBA	Het	TPSA	RotB	ArR	fSP2	logP	PolIdx	DAcompl	Flex	SizeComp	FG class	KeyGrp
1	Salicylic acid	138.12	2	3	3	57.5	1	1	1.00	+1.09	41.7	9	0.100	0.884	carboxylic	2
2	(S)-Mandelic acid	152.15	2	3	3	57.5	2	1	0.82	+0.81	37.8	9	0.182	0.803	carboxylic	1
3	Malonic acid	104.06	2	4	4	74.6	2	0	0.86	−0.45	71.7	10	0.286	0.871	carboxylic	1
4	Succinic acid	118.09	2	4	4	74.6	3	0	0.75	−0.06	63.2	10	0.375	0.968	carboxylic	1
5	Fumaric acid	116.07	2	4	4	74.6	2	0	1.00	−0.29	64.3	10	0.250	0.953	carboxylic	1
6	Glutaric acid	132.12	2	4	4	74.6	4	0	0.67	+0.33	56.5	10	0.444	0.924	carboxylic	1
7	Adipic acid	146.14	2	4	4	74.6	5	0	0.60	+0.72	51.0	10	0.500	0.836	carboxylic	1
8	Picolinic acid	123.11	1	3	3	50.2	1	1	1.00	+0.78	40.8	6	0.111	0.992	carboxylic	2
9	Naproxen	230.26	1	3	3	46.5	3	2	0.82	+3.04	20.2	6	0.176	0.530	carboxylic	1
10	Vanillin	152.15	1	3	3	46.5	2	1	0.91	+1.21	30.6	6	0.182	0.803	phenolic	1
11	Vanillic acid	168.15	2	4	4	66.8	2	1	0.92	+1.10	39.7	10	0.167	0.726	carboxylic	2
12	Phenylacetic acid	136.15	1	2	2	37.3	2	1	0.90	+1.31	27.4	5	0.200	0.897	carboxylic	1
13	3-Nitrophenol	139.11	1	4	4	63.4	1	1	1.00	+1.30	45.6	7	0.100	0.878	phenolic	1
14	3-Chloro-4-hydroxyphenylacetic acid	186.59	2	3	4	57.5	2	1	0.83	+1.67	30.8	9	0.167	0.655	carboxylic	2
15	p-Coumaric acid	164.16	2	3	3	57.5	2	1	1.00	+1.49	35.0	9	0.167	0.744	carboxylic	2
16	Phloretin	274.27	4	5	5	98.0	4	2	0.90	+2.33	35.7	17	0.200	0.445	phenolic	1
17	Benzoic acid	122.12	1	2	2	37.3	1	1	1.00	+1.39	30.5	5	0.111	1.000	carboxylic	1
18	4-Fluorobenzoic acid	140.11	1	2	3	37.3	1	1	0.90	+1.52	26.6	5	0.100	0.872	carboxylic	1
19	2-Fluorobenzoic acid	140.11	1	2	3	37.3	1	1	0.90	+1.52	26.6	5	0.100	0.872	carboxylic	1
20	Phthalic acid	166.13	2	4	4	74.6	2	1	1.00	+1.08	44.9	10	0.167	0.735	carboxylic	1
21	Isophthalic acid	166.13	2	4	4	74.6	2	1	1.00	+1.08	44.9	10	0.167	0.735	carboxylic	1
22	Terephthalic acid	166.13	2	4	4	74.6	2	1	1.00	+1.08	44.9	10	0.167	0.735	carboxylic	1
23	Trimesic acid	210.14	3	6	6	111.9	3	1	1.00	+0.78	53.3	15	0.200	0.581	carboxylic	1
24	Gentisic acid	154.12	3	4	4	77.8	1	1	1.00	+0.80	50.5	13	0.091	0.792	carboxylic	2
25	Gallic acid	170.12	4	5	5	98.0	1	1	1.00	+0.50	57.6	17	0.083	0.718	carboxylic	2
26	Caffeic acid	180.16	3	4	4	77.8	2	1	1.00	+1.20	43.2	13	0.154	0.678	carboxylic	2
27	Ferulic acid	194.19	2	4	4	66.8	3	1	0.93	+1.50	34.4	10	0.214	0.629	carboxylic	2
28	Syringic acid	198.17	2	5	5	76.0	3	1	0.86	+1.11	38.3	11	0.214	0.616	carboxylic	2
29	Protocatechuic acid	154.12	3	4	4	77.8	1	1	1.00	+0.80	50.5	13	0.091	0.792	carboxylic	2
30	2,6-Dihydroxybenzoic acid	154.12	3	4	4	77.8	1	1	1.00	+0.80	50.5	13	0.091	0.792	carboxylic	2
31	4-Hydroxybenzoic acid	138.12	2	3	3	57.5	1	1	1.00	+1.09	41.7	9	0.100	0.884	carboxylic	2
32	Nicotinic acid	123.11	1	3	3	50.2	1	1	1.00	+0.78	40.8	6	0.111	0.992	carboxylic	2
33	Isonicotinic acid	123.11	1	3	3	50.2	1	1	1.00	+0.78	40.8	6	0.111	0.992	carboxylic	2
34	Maleic acid	116.07	2	4	4	74.6	2	0	1.00	−0.29	64.3	10	0.250	0.953	carboxylic	1
35	Malic acid	134.09	3	5	5	94.8	3	0	0.67	−1.09	70.7	14	0.333	0.911	carboxylic	1
36	Tartaric acid	150.09	4	6	6	115.1	3	0	0.60	−2.12	76.7	18	0.300	0.814	carboxylic	1
37	Citric acid	192.12	4	7	7	132.1	5	0	0.69	−1.25	68.8	19	0.385	0.636	carboxylic	1
38	p-Toluenesulfonic acid	172.21	1	3	4	54.4	1	1	0.73	+1.24	31.6	6	0.091	0.709	strong_acid	1
39	Methanesulfonic acid	96.11	1	3	4	54.4	0	0	0.40	−0.50	56.6	6	0.000	0.824	strong_acid	1
40	Trifluoroacetic acid	114.02	1	2	5	37.3	0	0	0.43	+0.63	32.7	5	0.000	0.938	carboxylic	1
41	Naphthalene	128.17	0	0	0	0.0	0	2	1.00	+2.84	0.0	0	0.000	0.953	neutral	0
42	Biphenyl	154.21	0	0	0	0.0	1	2	1.00	+3.35	0.0	0	0.083	0.792	neutral	0

No	Coformer	MW	HBD	HBA	Het	TPSA	RotB	ArR	fSP2	logP	PolIdx	DAcompl	Flex	SizeComp	FG class	KeyGrp
43	Fluorene	166.22	0	0	0	0.0	0	2	0.92	+3.26	0.0	0	0.000	0.735	neutral	0
44	Hexamethylbenzene	162.28	0	0	0	0.0	0	1	0.50	+3.54	0.0	0	0.000	0.753	neutral	0
45	1,4-Dioxane	88.11	0	2	2	18.5	0	0	0.00	+0.03	21.0	2	0.000	0.782	neutral	0
46	Camphor	152.24	0	1	1	17.1	0	0	0.18	+2.40	11.2	1	0.000	0.802	neutral	0
47	Riboflavin	376.37	5	10	10	161.6	5	1	0.59	−1.72	42.9	25	0.185	0.324	base	1
48	Picolinamide	122.13	1	3	3	56.0	1	1	1.00	+0.18	45.8	6	0.111	1.000	amide	2
49	Nicotinamide	122.13	1	3	3	56.0	1	1	1.00	+0.18	45.8	6	0.111	1.000	amide	2
50	Isonicotinamide	122.13	1	3	3	56.0	1	1	1.00	+0.18	45.8	6	0.111	1.000	amide	2

a Descriptors were computed programmatically from the coformer SMILES using RDKit. The derived numerical columns (D10, D12 and D13–D15) complete the general-descriptor block; their definitions are given in Table S6.

Table S6. Numerical definition of the 24 descriptors used in the PCA.

Code	Descriptor	Definition
D01_HBD	Hydrogen-bond donor count	rdkit.Chem.Lipinski.NumHDonors
D02_HBA	Hydrogen-bond acceptor count	rdMolDescriptors.CalcNumLipinskiHBA (N + O count)
D03_Hetero	Total heteroatom count	number of N, O, S, F, Cl, Br and I atoms
D04_MW	Molecular weight	rdkit.Chem.Descriptors.MolWt
D05_TPSA	Topological polar surface area	rdkit.Chem.Descriptors.TPSA
D06_RotB	Rotatable-bond count	rdkit.Chem.Descriptors.NumRotatableBonds
D07_ArRings	Aromatic-ring count	rdMolDescriptors.CalcNumAromaticRings
D08_fSP2	Fraction of sp2 atoms	(aromatic or sp2 heavy atoms) / (heavy atoms)
D09_logP	Calculated lipophilicity	rdkit.Chem.Crippen.MolLogP
D10_PolIdx	Molecular polarity index	100 × TPSA / MW
D11_FGclass	Functional-group class	ordinal SMARTS-based code: neutral 0, amide or base 1, phenolic 2, carboxylic 3, sulfonic 4
D12_KeyGroups	Key cocrystal-group presence	number of distinct groups matched among carboxylic acid, phenol, amide, aromatic N and sulfonic acid (0–5)
D13_DAcompl	Donor–acceptor complementarity	HBD(coformer) × HBA(API) + HBA(coformer) × HBD(API), with HBA(API) = 3 and HBD(API) = 1
D14_Flex	Molecular flexibility	rotatable bonds / heavy atoms
D15_SizeComp	Shape/size compatibility	1 / (1 + |MW(coformer) − MW(API)| / MW(API)), MW(API) = 122.126
D16_RelPos	Relative pyridine-N/amide position	ordinal geometry code: ortho 1, meta 2, para 3
D17_pyrNavail	Pyridine-N acceptor availability	ordinal code: PIC 1, NIC 2, INIC 3
D18_amNHacc	Amide N–H donor accessibility	ordinal code: PIC 1, NIC 3, INIC 3
D19_amCOacc	Amide carbonyl acceptor accessibility	ordinal code: PIC 2, NIC 3, INIC 3
D20_AcidPyr	Acid–pyridine heterosynthon likelihood	acid strength score × D17; acid score 3 (sulfonic), 2 (carboxylic), 1 (phenolic), 0 otherwise
D21_AcidAmide	Acid–amide interaction likelihood	acid strength score × (D18 + D19) / 2
D22_AmideAmide	Amide–amide homosynthon possibility	amide flag of the coformer × (D18 + D19) / 2
D23_ArSurf	Aromatic-surface compatibility	D07 × D08
D24_Compete	Homo/heteromolecular H-bond competition	min(HBD, HBA) / (1 + min(HBD, 3) + min(HBA, 1))

Table S7. Named exceptions to the cocrystal-likely / no-cocrystal group centroids in PC1–PC3 (2-SD criterion).[a]

For each predicted class (cocrystal-likely, $p \geq 0.50$; no-cocrystal, $p < 0.50$), the Euclidean distance of every API-coformer pair from its own group's centroid in PC1–PC3 space was normalized by the within-group standard deviation along each axis (a per-axis z-score). A pair was flagged as a named exception when this normalized distance exceeded 2.0. Applying this criterion identified 30 cocrystal-likely and 3 no-cocrystal pairs as named exceptions, each annotated with the single original 24-descriptor variable contributing most to its deviation and that descriptor's own z-score. Rows are sorted by decreasing distance from the group centroid.

Group	API	Coformer	Dist. from centroid (SD)	PC1	PC2	PC3	Dominant deviating descriptor	Descriptor z-score
No-cocrystal	PIC	Riboflavin	3.64	+7.11	−6.09	+3.57	Hydrogen-bond donor count	+2.48
Cocrystal-likely	PIC	Phloretin	3.32	+2.08	−4.50	+4.27	Molecular weight	+3.45
Cocrystal-likely	INIC	Phloretin	3.17	+2.56	−0.20	+5.46	Molecular weight	+3.45
No-cocrystal	NIC	Riboflavin	3.15	+7.29	−3.31	+4.47	Hydrogen-bond donor count	+2.48
Cocrystal-likely	PIC	Biphenyl	3.14	−6.76	−2.87	+1.69	Functional-group class	−2.94
Cocrystal-likely	INIC	Fluorene	3.13	−6.74	+0.92	+2.98	Functional-group class	−2.94
Cocrystal-likely	PIC	Naphthalene	3.12	−7.21	−2.58	+0.95	Functional-group class	−2.94
No-cocrystal	INIC	Riboflavin	3.11	+7.36	−2.29	+4.81	Hydrogen-bond donor count	+2.48
Cocrystal-likely	NIC	Phloretin	3.07	+2.43	−1.38	+5.14	Molecular weight	+3.45
Cocrystal-likely	INIC	Biphenyl	3.05	−6.51	+0.93	+2.93	Functional-group class	−2.94
Cocrystal-likely	INIC	Naphthalene	3.01	−6.96	+1.22	+2.19	Functional-group class	−2.94
Cocrystal-likely	PIC	1,4-Dioxane	2.92	−4.43	−2.57	−3.62	Fraction of sp2 atoms	−3.91
Cocrystal-likely	PIC	Hexamethylbenzene	2.88	−6.55	−2.69	−0.46	Functional-group class	−2.94
Cocrystal-likely	PIC	Citric acid	2.80	+5.89	−3.67	−1.20	Rotatable-bond count	+2.50
Cocrystal-likely	PIC	Tartaric acid	2.64	+4.80	−3.11	−2.58	Calculated lipophilicity (logP)	−2.71
Cocrystal-likely	INIC	Hexamethylbenzene	2.54	−6.30	+1.12	+0.78	Functional-group class	−2.94
Cocrystal-likely	NIC	Hexamethylbenzene	2.49	−6.36	+0.10	+0.44	Functional-group class	−2.94
Cocrystal-likely	INIC	Citric acid	2.36	+6.61	+1.14	−0.06	Rotatable-bond count	+2.50
Cocrystal-likely	PIC	Malic acid	2.33	+3.12	−2.66	−3.02	Calculated lipophilicity (logP)	−1.79
Cocrystal-likely	INIC	Naproxen	2.32	−0.91	+1.78	+3.80	Molecular weight	+2.19
Cocrystal-likely	INIC	Methanesulfonic acid	2.28	+0.52	+3.86	−2.73	Acid–pyridine heterosynthon likelihood	+2.59
Cocrystal-likely	NIC	Citric acid	2.26	+6.40	−0.20	−0.38	Rotatable-bond count	+2.50
Cocrystal-likely	PIC	Naproxen	2.25	−1.63	−3.02	+2.66	Molecular weight	+2.19
Cocrystal-likely	NIC	1,4-Dioxane	2.22	−4.25	+0.22	−2.72	Fraction of sp2 atoms	−3.91
Cocrystal-likely	PIC	Methanesulfonic acid	2.20	−0.43	−1.44	−3.82	Fraction of sp2 atoms	−2.06
Cocrystal-likely	INIC	Tartaric acid	2.18	+5.52	+1.69	−1.44	Calculated lipophilicity (logP)	−2.71
Cocrystal-likely	PIC	Succinic acid	2.15	+1.61	−2.28	−3.28	Molecular flexibility	+1.74
Cocrystal-likely	INIC	1,4-Dioxane	2.15	−4.18	+1.24	−2.38	Fraction of sp2 atoms	−3.91
Cocrystal-likely	PIC	Trifluoroacetic acid	2.13	−1.64	−1.80	−3.31	Fraction of sp2 atoms	−1.92
Cocrystal-likely	PIC	Malonic acid	2.11	+1.50	−2.23	−3.24	Molecular polarity index	+1.64
Cocrystal-likely	PIC	Glutaric acid	2.07	+1.82	−2.45	−2.95	Molecular flexibility	+2.33
Cocrystal-likely	NIC	Tartaric acid	2.06	+5.32	+0.36	−1.76	Calculated lipophilicity (logP)	−2.71
Cocrystal-likely	PIC	Trimesic acid	2.06	+2.82	−3.47	+1.18	Hydrogen-bond acceptor count	+1.73

[a] "Group" is the DeepCocrystal-predicted class; "Dist. from centroid (SD)" is the joint PC1–PC3 distance from the pair's own group centroid, expressed in within-group standard deviations; "Descriptor z-score" is the standardized deviation of the dominant descriptor relative to its own group's mean. All descriptor values were computed from RDKit-derived molecular structures as tabulated in the complete 24-descriptor matrix.

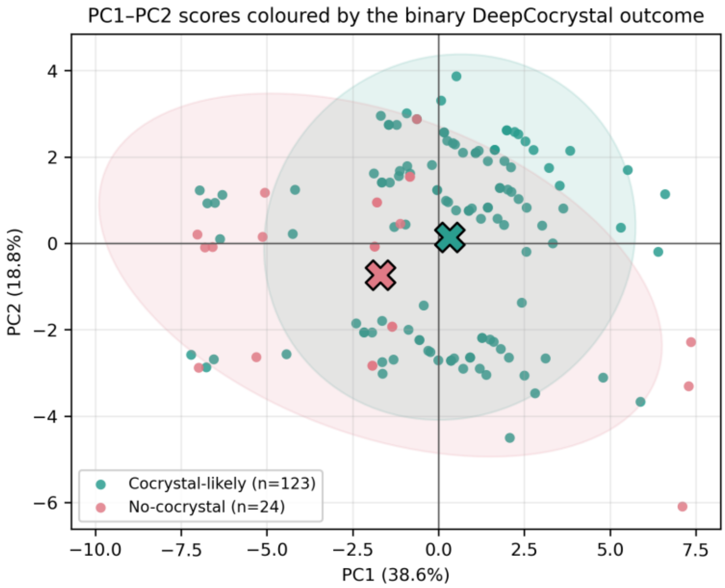

Figure S1. PC1–PC2 score plot colored by DeepCocrystal binary outcome ($p \geq 0.5$ cocrystal-likely vs $p < 0.5$ no-cocrystal). Bold crosses mark the class centroids and the shaded ellipses the 2-sigma covariance of each class. No-cocrystal pairs are displaced toward the negative end of PC1.

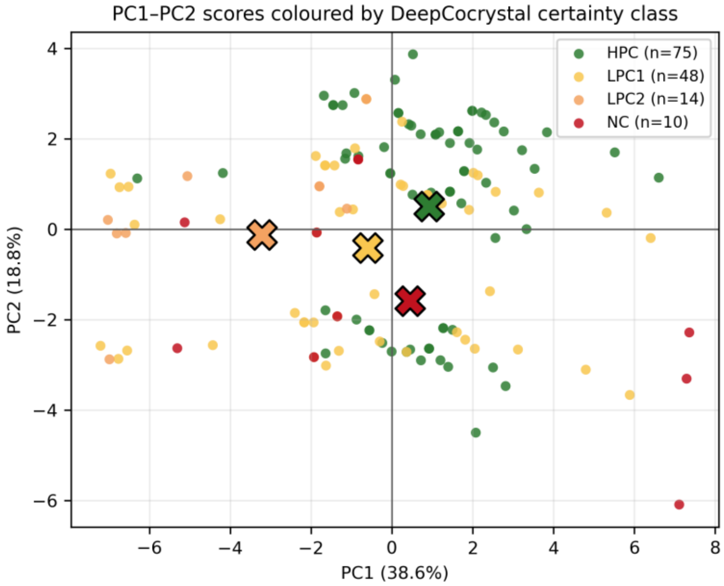

Figure S2. PC1–PC2 score plot colored by the four DeepCocrystal certainty classes (HPC, LPC1, LPC2, NC). The HPC centroid lies at the positive end of PC1 and the LPC2 centroid at the opposite end, so the certainty of the prediction follows the same axis as the polarity and donor–acceptor complementarity of the coformer.

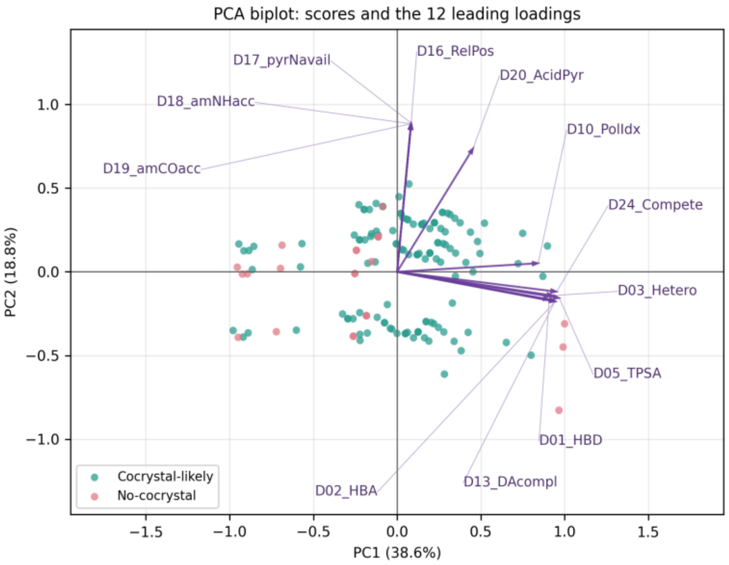

Figure S3. Biplot overlaying the PC1–PC2 scores (colored by predicted class) with the twelve descriptors of largest combined loading on PC1 and PC2. Arrow tips give the correlation loadings; the scores are shown on a common scale for the two components and are therefore directly comparable with Figures S1 and S2. Leader lines connect each arrow to its label.

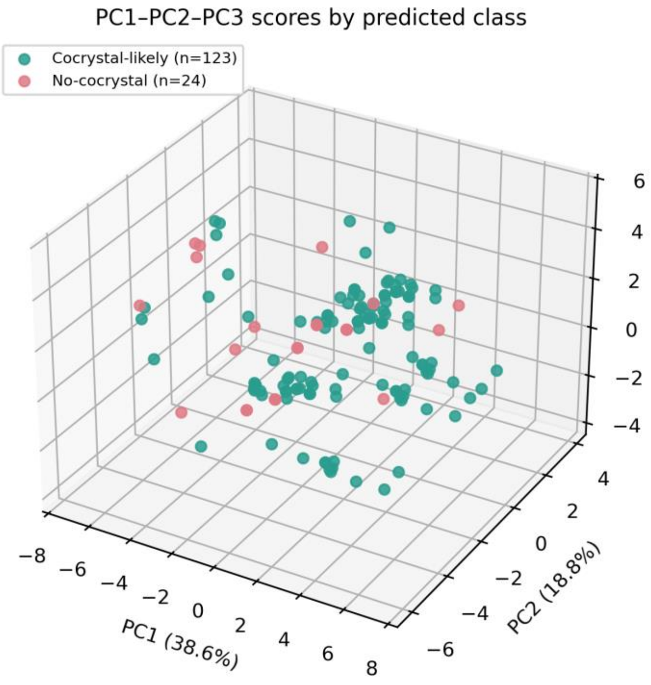

Figure S4. Three-dimensional PCA score plot (PC1–PC2–PC3) colored by predicted cocrystal class.

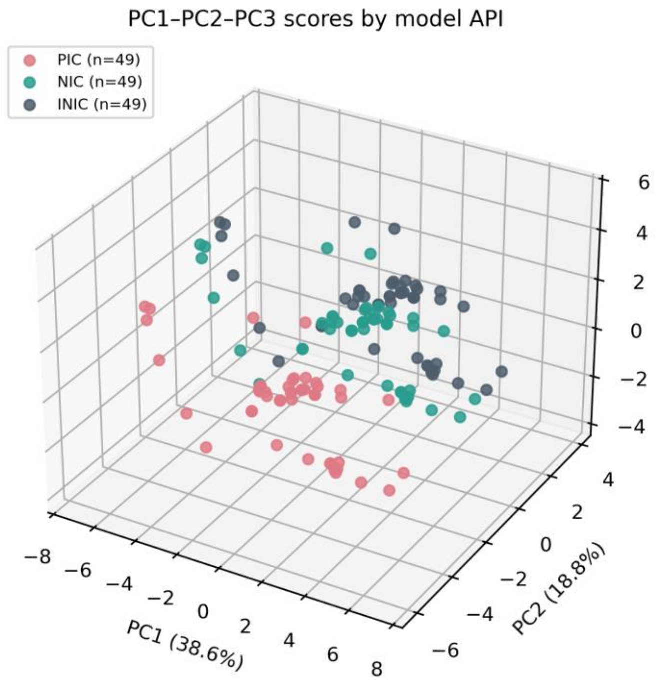

Figure S5. Three-dimensional PCA score plot colored by model API, showing the systematic but small API offset superimposed on the shared coformer-driven distribution.

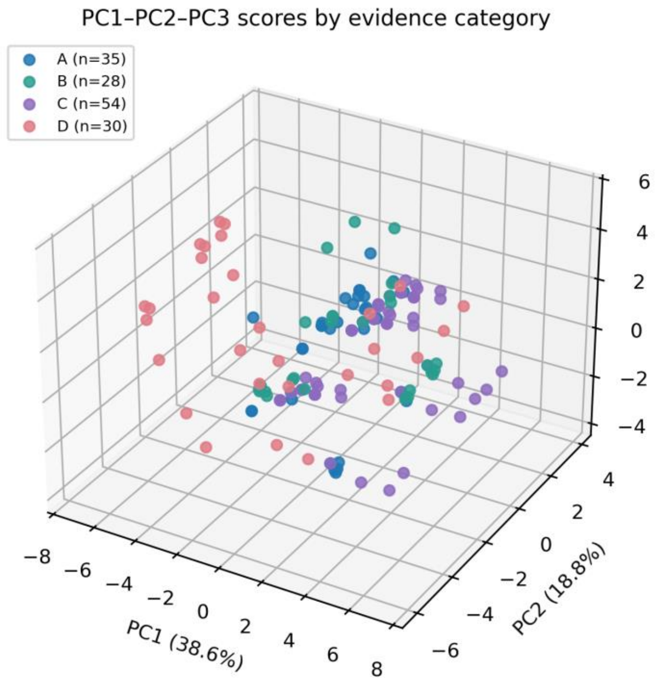

Figure S6. Three-dimensional PCA score plot colored by evidence category. Group D is displaced toward the negative end of PC1 (centroid −3.06) and group C toward the positive end (+1.76), while groups A and B occupy the central region.

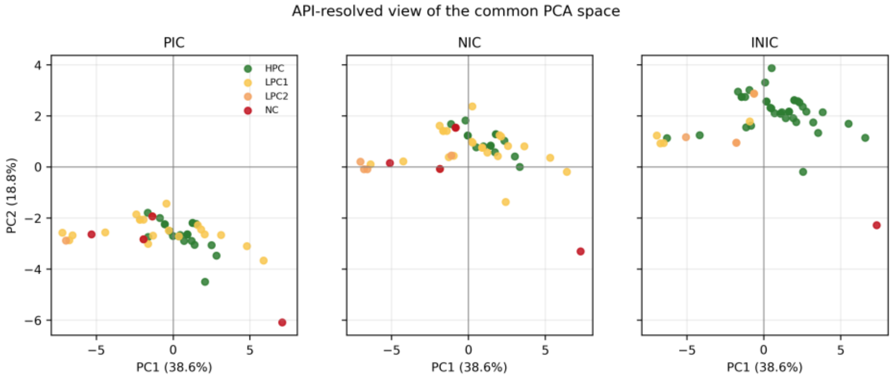

Figure S7. The three model APIs shown separately within the common PC1–PC2 space, colored by DeepCocrystal class. The coformer cloud is shared; the panels differ only in how each scaffold's predictions populate it (note the near-absence of negative classes for isonicotinamide).

References

1 Chan, H.C.S.; Woollam, G.R.; Wagner, T.; Schmidt, M.U.; Lewis, R.A. Can Picolinamide Be a Promising Cocrystal Former? *CrystEngComm* 2014, 16, 4365–4368.

2 Ganduri, R.; Cherukuvada, S.; Guru Row, T.N. Pyridinecarboxamide Cocrystals: Picolinamide, Nicotinamide and Isonicotinamide with Pyridinecarboxylic Acids. *CrystEngComm* 2017, 19, 1123–1132.

3 Kerr, H.E.; Softley, L.K.; Suresh, K.; Hodgkinson, P.; Evans, I.R. Structure Determination of Naproxen–Picolinamide Molecular Complexes. *Acta Crystallogr. Sect. C* 2017, 73, 168–175.

4 Ouyang, J.; Hu, J.; Sun, C. Crystal Engineering of Vanillin Cocrystals with Pyridinecarboxamide Coformers. *CrystEngComm* 2023, 25, 2038–2051.

5 Sarkar, N.; Gonnella, N.C.; Krawiec, M.; Xin, D.; Aakeröy, C.B. Evaluating the Predictive Abilities of Protocols Based on Hydrogen-Bond Propensity, Molecular Complementarity, and Hydrogen-Bond Energy for Cocrystal Screening. *Cryst. Growth Des.* 2020, 20, 7320–7327.

6 Jacobs, A.; Amombo Noa, F.M. Vanillic Acid Cocrystals with Nicotinamide and Isonicotinamide. *CrystEngComm* 2015, 17, 98–106.

7 Bhogala, B.R.; Nangia, A. Cocrystals of 1,3,5-Benzenetricarboxylic Acid with 4,4′-Bipyridines and Isonicotinamide: Crystal Engineering Using O–H···N and N–H···O Hydrogen Bonds. *J. Mol. Struct.* 2008, 885, 45–49.

8 Noa, F.M.M.A.; Bourne, S.A.; Caira, M.R. Phenylacetic Acid Cocrystals with Nicotinamide and Isonicotinamide. *J. Mol. Struct.* 2017, 1139, 60–66.

9 Komorowska, M.; Szafran, M.; Dega-Szafran, Z.; Katrusiak, A. Molecular Complexes of 3-Nitrophenol with Nicotinamide and Isonicotinamide. *J. Mol. Struct.* 2013, 1047, 310–316.

10 Lemmerer, A.; Bernstein, J.; Kahlenberg, V. 3-Chloro-4-hydroxyphenylacetic Acid Cocrystals with Nicotinamide and Isonicotinamide. *J. Mol. Struct.* 2020, 1201, 127297.

11 Bevill, M.J.; Vlahova, P.I.; Smit, J.P. Polymorphic Cocrystals of p-Coumaric Acid and Nicotinamide. *Cryst. Growth Des.* 2014, 14, 1438–1448.

12 Aitipamula, S.; Chow, P.S.; Tan, R.B.H. Phloretin–Nicotinamide Cocrystal Polymorphs. *CrystEngComm* 2022, 24, 560–570.

13 Dubey, R.; Desiraju, G.R. Structural Landscape of the 1:1 Benzoic Acid:Isonicotinamide Cocrystal. *Chem. Commun.* 2014, 50, 1181–1184.

14 Childs, S.L.; Stahly, G.P.; Park, A. The Salt–Cocrystal Continuum: The Influence of Crystal Structure on Ionization State. *Mol. Pharm.* 2007, 4, 323–338.

15 Desiraju, G.R. Supramolecular Synthons in Crystal Engineering—a New Organic Synthesis. *Angew. Chem. Int. Ed.* 1995, 34, 2311–2327.

16 Cruz-Cabeza, A.J. Acid–Base Crystalline Complexes and the pK_a Rule. *CrystEngComm* 2012, 14, 6362–6365.

17 Zhang, G.G.Z.; Henry, R.F.; Borchardt, T.B.; Lou, X. Efficient Co-crystal Screening Using Solution-Mediated Phase Transformation. *J. Pharm. Sci.* 2007, 96, 990–1005.

18 Thompson, H.P.; Day, G.M.; McCabe, J.F.; Pidcock, E.; Motherwell, W.D.S.; Shields, G.P. Nicotinamide–Succinic Acid Cocrystal. *Acta Crystallogr. Sect. C* 2010, 66, o421–o424.

19 Orola, L.; Veidis, M.V. Nicotinamide Fumaric Acid Supramolecular Cocrystals: Diversity of Stoichiometry. *CrystEngComm* 2009, 11, 415–417.

20 Serjeant, E.P.; Dempsey, B. Ionisation Constants of Organic Acids in Aqueous Solution; IUPAC Chemical Data Series No. 23; Pergamon Press: Oxford, 1979.

21 Lowe, B.M.; Smith, D.G. Dissociation Constant of 2,6-Dihydroxybenzoic Acid in Water and in Deuterium Oxide at 25 °C. J. Chem. Soc., Faraday Trans. 1 1973, 69, 1934–1944.
